# Diastereomeric dinickel(ii) complexes with non-innocent bis(octaazamacrocyclic) ligands: isomerization, spectroelectrochemistry, DFT calculations and use in catalytic oxidation of cyclohexane†

**DOI:** 10.1039/d2dt00154c

**Published:** 2022-02-28

**Authors:** Anatolie Dobrov, Denisa Darvasiová, Michal Zalibera, Lukáš Bučinský, Ingrid Jelemenská, Peter Rapta, Sergiu Shova, Dan G. Dumitrescu, Marta A. Andrade, Luísa M. D. R. S. Martins, Armando J. L. Pombeiro, Vladimir B. Arion

**Affiliations:** University of Vienna, Institute of Inorganic Chemistry Währinger Strasse 42 A-1090 Vienna Austria vladimir.arion@univie.ac.at; Universität Wien, Fakultät für Chemie, Institut für Biophysikalische Chemie 1090 Wien Austria; Institute of Physical Chemistry and Chemical Physics, Faculty of Chemical and Food Technology, Slovak University of Technology in Bratislava Radlinského 9 SK-81237 Bratislava Slovak Republic michal.zalibera@stuba.sk; Department of Chemistry, Faculty of Natural Sciences, Constantine the Philosopher University in Nitra 949 74 Nitra Slovak Republic; Inorganic Polymers Department, “Petru Poni” Institute of Macromolecular Chemistry Aleea Gr. Ghica Voda 41 A Iasi 700487 Romania; Elettra – Sincrotrone Trieste S.C.p.A. Strada Statale 14 – km 163 5 in AREA Science Park 34149 Basovizza Trieste Italy; Centro de Química Estrutural, Institute of Molecular Sciences, Universidade de Lisboa Av. Rovisco Pais 1049-001 Lisboa Portugal luisamargaridamartins@tecnico.ulisboa.pt; Peoples’ Friendship University of Russia (RUDN University), Research Institute of Chemistry 6 Miklukho-Maklaya Street Moscow 117198 Russian Federation

## Abstract

Diastereomeric dinickel(ii) complexes with bis-octaazamacrocyclic 15-membered ligands [Ni(L^1–3^–L^1–3^)Ni] (4–6) have been prepared by oxidative dehydrogenation of nickel(ii) complexes NiL^1–3^ (1–3) derived from 1,2- and 1,3-diketones and *S*-methylisothiocarbohydrazide. The compounds were characterized by elemental analysis, ESI mass spectrometry, and IR, UV–vis, ^1^H NMR, and ^13^C NMR spectroscopy. Single crystal X-ray diffraction (SC-XRD) confirmed the isolation of the *anti* and *syn* isomers of bis-octaazamacrocyclic dinickel(ii) complexes 4a and 4s, the *syn*-configuration of 5s and the *anti*-configuration of the dinickel(ii) complex 6a. Dimerization of prochiral nickel(ii) complexes 1–3 generates two chiral centers at the bridging carbon atoms. The *anti*-complexes were isolated as *meso*-isomers (4a and 6a) and the *syn*-compounds as racemic mixtures of *R*,*R*/*S*,*S*-enantiomers (4s and 5s). The *syn-anti* isomerization (epimerization) of the isolated complexes in chloroform was disclosed. The isomerization kinetics of 5a was monitored at five different temperatures ranging from 20 °C to 50 °C by ^1^H NMR spectroscopy indicating the clean conversion of 5a into 5s. The activation barrier determined from the temperature dependence of the rate constants *via* the Eyring equation was found to be Δ*H*^‡^ = 114 ± 1 kJ mol^−1^ with activation entropy Δ*S*^‡^ = 13 ± 3 J K^−1^ mol^−1^. The complexes contain two low-spin nickel(ii) ions in a square-planar coordination environment. The electrochemical behavior of 4a, 4s, 5s and 6a and the electronic structure of the oxidized species were studied by UV–vis–NIR-spectroelectrochemistry (SEC) and DFT calculations indicating the redox non-innocent behavior of the complexes. The dinickel(ii) complexes 4a, 4s, 5s and 6a/6s were investigated as catalysts for microwave-assisted solvent-free oxidation of cyclohexane by *tert*-butyl hydroperoxide to produce a mixture of cyclohexanone and cyclohexanol (KA oil). The best value for KA oil yield (16%) was obtained with a mixture of 6a/6s after 2 h of microwave irradiation at 100 °C.

## Abstract 2

Dinickel(ii) complexes with non-innocent bis(octaazamacrocyclic) ligands were isolated as *R*,*S*/*S*,*R* and *R*,*R*/*S*,*S*-enantiomers.

## Graphical abstract

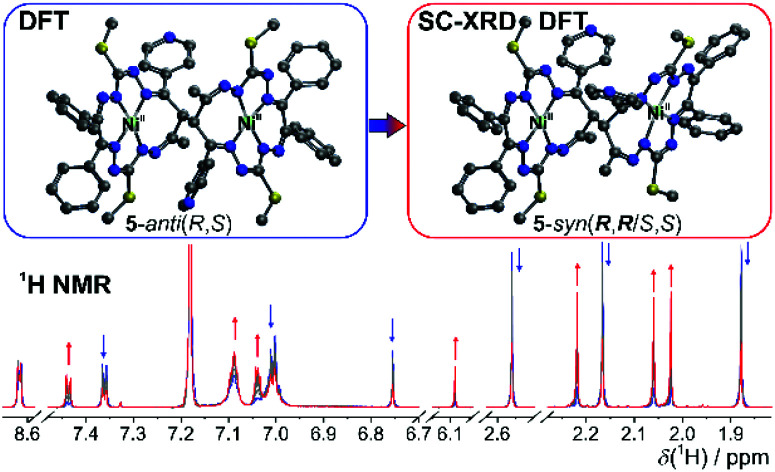

## Introduction

Transition metal complexes with redox non-innocent ligands are extensively studied in coordination chemistry to understand their electronic structure and bonding,^1–6^ as well as to exploit their potential applicability in stoichiometric reactions^7,8^ and catalytic transformations^9–11^ inspired by metalloenzymes, *e.g.*, galactose oxidase.^12,13^ While the number of mononuclear complexes with redox non-innocent ligands is large and permanently increases, the known examples of metal complexes with non-innocent dinucleating ligands are still scarce.^14–18^ However, such complexes might be of interest in multielectron catalysis,^19–21^ in which cooperation of several redox-active centers (metals and ligands) is required.

**Chart 1:**
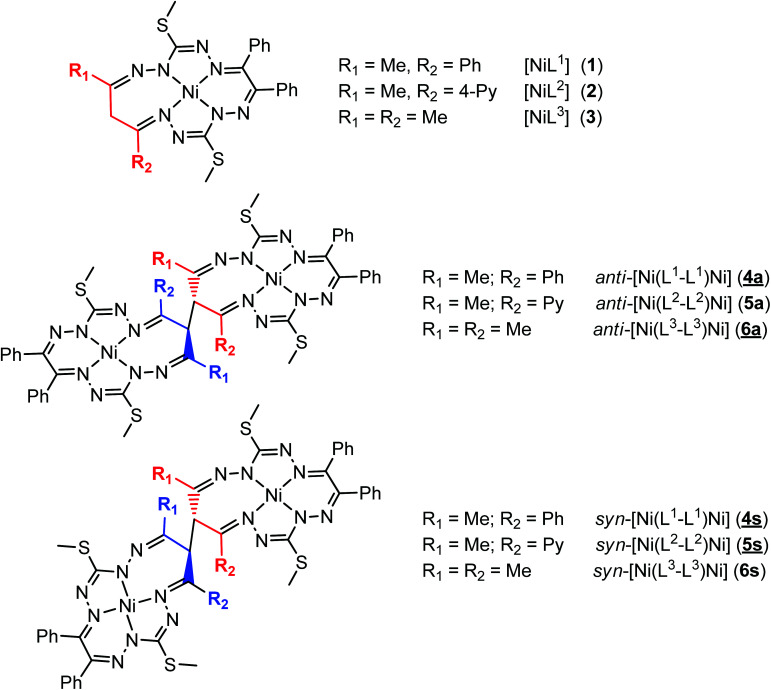
Starting materials and complexes reported in this work. Underlined numbers indicate compounds studied by SC-XRD.

Quite recently, we described the template synthesis of nickel(ii) complexes with 15-membered octaazamacrocyclic ligands 1–3 (Chart 1) and their structures, as well as the spectroscopic properties of the 1e-oxidized and 1e-reduced species.^22,23^ We also explored their ability to catalyze the microwave-assisted solvent-free oxidation of cyclohexane by *tert*-butyl hydroperoxide (TBHP) to the industrially significant KA oil (a cyclohexanone and cyclohexanol mixture). One issue that remained unaddressed in the reported works is whether these coordinated macrocycles can be joined into dimers *via* C–C bond formation between the γ-carbon atoms of the former Hacac moieties, even though these are now constituent atoms of a seven-membered chelate ring (Ni1NCCCNN1, see Chart 1). All oxidative dehydrogenation reactions reported so far occurred at the central carbon atom of the six-membered 1,3-diketone-β-diiminato rings leading to either C–C bond formation^14,24–33^ or the generation of new ketone products.^34–36^ The incorporation of six-membered 1,3-diketone-diiminato moieties in monomacrocyclic metal complexes predetermined the formation of dimetallic species of two-fold or even higher molecular symmetry *via* C–C bond coupling. In the present case, the use of 1,3-diketones in template condensation reaction results in the formation of a low symmetry seven-membered folded chelate ring containing one N–N unit (Scheme S1 in the ESI†). This feature may allow for geometric isomerism in dinickel(ii) bis(octaazamacrocyclic) complexes. Moreover, the dimerization would lead to the appearance of two chiral centers. In this context, it should be noted that enantiomeric isomerization plays a great role not only in biology^37–39^ and food chemistry^40,41^ but also in materials science.^42,43^ Examples of diastereomeric isomerization for metal complexes are scarce but well-documented in the literature. For instance, a thiolato-bridged Ru^II^Ag^I^Ru^II^ complex with d-penicillamine (d-H_2_pen), prepared from [RuCl_2_(bpy)_2_] (bpy = 2,2′-bipyridine), d-H_2_pen and Ag^+^, afforded three isomers Δ_D_Δ_D_, Λ_D_Λ_D_ and Δ_D_Λ_D_ that were isolated by fractional crystallization. Interestingly, the first isomer, in contrast to the other two, exhibited thermal linkage isomerism, consisting of the change in the coordination mode of d-H_2_pen from bidentate O, S to bidentate N, S.^44^ Chiral-at-metal (η^6^-*p*-cymene)ruthenium(ii) and (η^6^-*p*-cymene)osmium(ii) half-sandwich complexes with chiral bidentate Schiff bases were reported to crystallize as diastereomers with opposite configurations at the metal centers in a 1 : 1 ratio or as pure enantiomers.^45^ The complexes were used in the enantioselective isomerization of substrates for the synthesis of natural products.

Herein, we report on the synthesis of diastereomeric dinickel(ii) complexes 4–6 with new bis(octaazamacrocyclic) ligands from 1–3 (Chart 1) by oxidative dehydrogenation. In all cases, geometrical *anti* and *syn* isomers or *meso* (*S*,*R*/*R*,*S*) and racemic mixtures of *S*,*S* and *R*,*R* enantiomers were separated chromatographically, isolated, and characterized by analytical and spectroscopic techniques, as well as single-crystal X-ray diffraction (SC-XRD) (4a and 4s, 5s and 6a). In addition, their isomerization (epimerization) in chloroform was investigated by ^1^H NMR spectroscopy. Moreover, the redox behavior of the dimeric complexes was studied by electrochemical (EC) and/or spectroelectrochemical (SEC) techniques, while the electronic structure was studied by theoretical DFT methods. The catalytic performance of these complexes towards the microwave-assisted solvent-free oxidation of cyclohexane by TBHP was also explored and compared to those of the mononuclear nickel(ii) complexes reported previously, under the same reaction conditions. In fact, selective functionalization of non-activated aliphatic C–H bonds is a very powerful approach as inert, widely available compounds are transformed into high value added functionally diverse products.^46,47^ KA oil, *i.e.*, cyclohexanone (K, ketone) and cyclohexanol (A, alcohol), is used in the manufacturing process of adipic acid, a relevant commodity, with a production of over 3.5 million metric tons per year, growing at *ca*. 5% annually.^48^ The current industrial process for homogeneous cyclohexane oxidation involves a Co-catalyzed two-step oxidation process at high temperatures (150–180 °C) to KA oil followed by nitric acid oxidation to adipic acid. This process bears major drawbacks for being able to generate no more than 5–12% of KA oil yields (to maintain a reasonable KA oil selectivity of 70–80%) and requiring a huge excess of nitric acid for KA oil oxidation. This inert C–H bond oxyfunctionalization has long been dominated by transition metal catalysis.^49–53^

## Results and discussion

### Synthesis of complexes

The oxidation of chloroform solutions of 1–3 with FeCl_3_·6H_2_O in ethanol in the presence of pyridine resulted in dinuclear geometric isomers 4a, 4s, 5a (minor) and 5s and complexes 6a and 6s with different *R*_f_ values, which were separated by column chromatography (Chart 1) and isolated in good yields in all cases but 5a, where a means *anti* and s means *syn*. Pyridine was used for the stabilization of the reduction product iron(ii) as [Fe(py)_6_]Cl_2_.^54^ The formation of dimeric species *via* C–C coupling of the two macrocycles at the central carbon atom of the 1,3-diketone moiety is evidenced by positive ion ESI mass spectra, where the peaks at *m*/*z* 1193 and 1215 for 4a and 4s and 1195 and 1217 for 5a and 5s were found and were attributed to the [M + H]^+^ and [M + Na]^+^ ions and that at *m*/*z* 1069 (for 6a and 6s) was assigned to [M + H]^+^. The ^1^H and ^13^C NMR spectra provided further evidence for the formation of two geometric isomers, *anti* and *syn*, for complexes 4–6. It should be also stressed that other isomers were not isolated, even though two geometric isomers of the ligands in compounds 1 and 2 by interchanging R_1_ and R_2_ were actually expected (see Scheme S1 in the ESI†).^22^ A likely reason is the steric hindrance created by one of the two rotating thiomethyl groups at the terminal non-coordinated NH_2_ group. The formation and isolation of isomers 4a and 4s, as well as 5s along with 6a and C–C bond coupling between two octaazamacrocyclic moieties, was finally confirmed by SC-XRD studies.

### X-ray crystallography

**Fig. 1:**
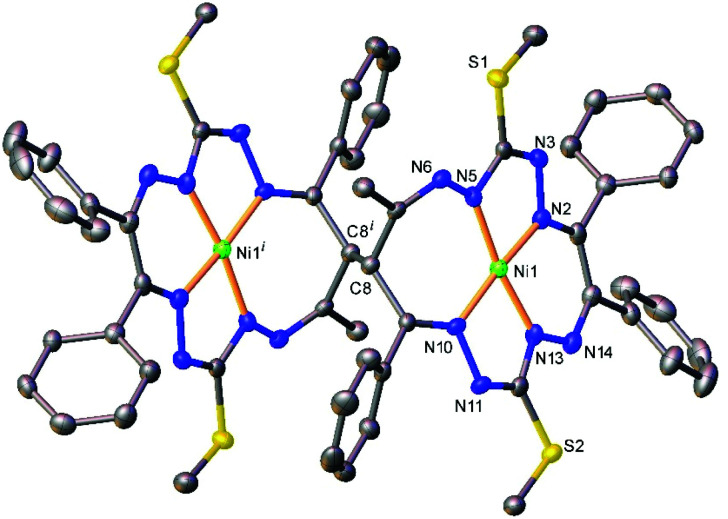
ORTEP view of the dimeric molecule of 4a (or *meso-R*,*S*-isomer, where C8 has absolute configuration *R*) with atom labeling and thermal ellipsoids at the 50% probability level. H-Atoms are not shown. Symmetry code: (*i*) 1 − *x*, 2 − *y*, − *z.* Selected bond distances (Å) and angles (°): Ni1–N2 = 1.832(3), Ni1–N5 = 1.864(3), Ni1–N10 = 1.889(3), Ni1–N13 = 1.809(3), C8–C8^*i*^ = 1.573(6); N2–Ni1–N5 = 83.52(12), N2–Ni1–N10 = 173.73(12), N5–Ni1–N10 = 102.25(12), N13–Ni1–N2 = 91.77(12), N13–Ni1–N5 = 171.46(13), N13–Ni1–N10 = 82.40(12).

**Fig. 2:**
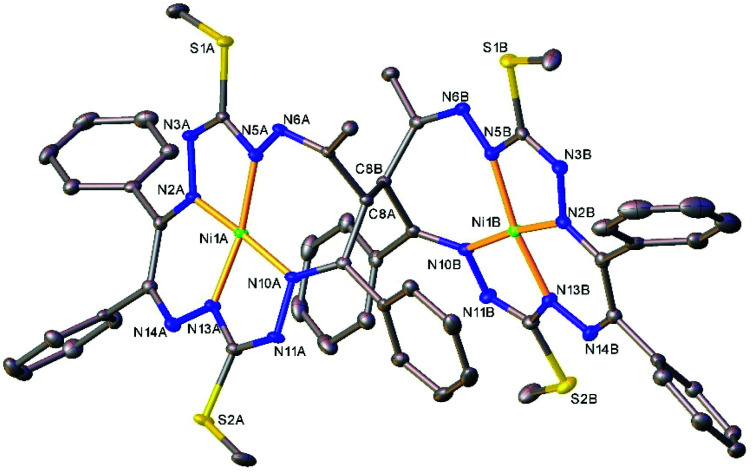
ORTEP view of the molecular structure of 4s (or *S*,*S*-enantiomer) with atom labeling and thermal ellipsoids at the 50% probability level. H-Atoms are not shown. Selected bond distances (Å) and angles (°) for A and (B), respectively: Ni1–N2 = 1.850(2) (1.853(2)), Ni1–N5 = 1.868(2) (1.868(2)), Ni1–N10 = 1.906(2) (1.903(2), Ni1–N13 = 1.806(2) (1.806(2)), C8A–C8B = 1.573(2); N2–Ni1–N5 = 83.16(7) (83.65(7), N2–Ni1–N10 = 172.35(7) (173.13(7)), N5–Ni1–N10 = 102.88(7) (102.44(7)), N13–Ni1–N2 = 92.30(7) (91.84(7)), N13–Ni1–N5 = 169.01(8) (170.96(8), N13–Ni1–N10 = 82.50(7) (82.50(7)).

**Fig. 3:**
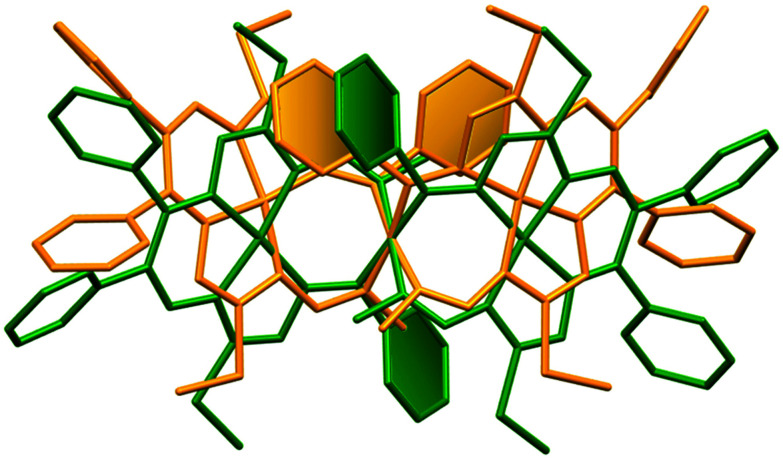
Overlay of 4a or *S*,*R*-isomer (green trace) and 4s or *S*,*S*-isomer (yellow) along the C–C bond.

**Fig. 4:**
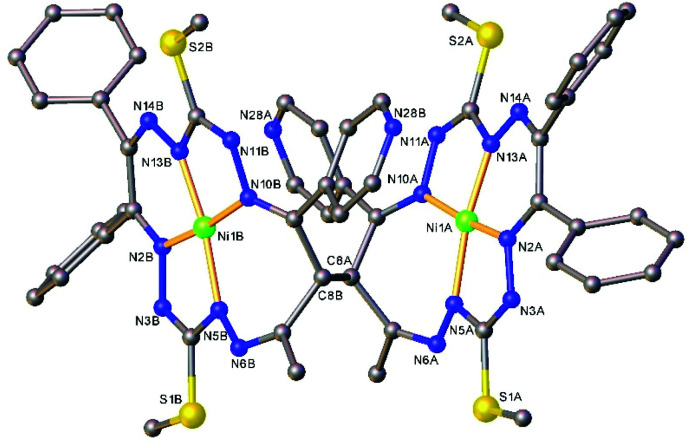
Ball-and-stick representation of the molecular structure of 5s (or *R*,*R*-enantiomer). H-Atoms are not shown. Selected bond distances (Å) and angles (°) for A and (B), respectively: Ni1–N2 = 1.844(3) (1.839(3)), Ni1–N5 = 1.875(3) (1.882(4)), Ni1–N10 = 1.896(3) (1.892(3), Ni1–N13 = 1.808(3) (1.793(4)), C8A–C8B = 1.570(5); N2–Ni1–N5 = 83.42(14) (83.05(16), N2–Ni1–N10 = 173.19(13) (173.28(14)), N5–Ni1–N10 = 102.37(14) (103.16(15)), N13–Ni1–N2 = 91.98(13) (91.32(16)), N13–Ni1–N5 = 171.17(14) (171.65(14), N13–Ni1–N10 = 82.74(13) (82.78(15)).

**Fig. 5:**
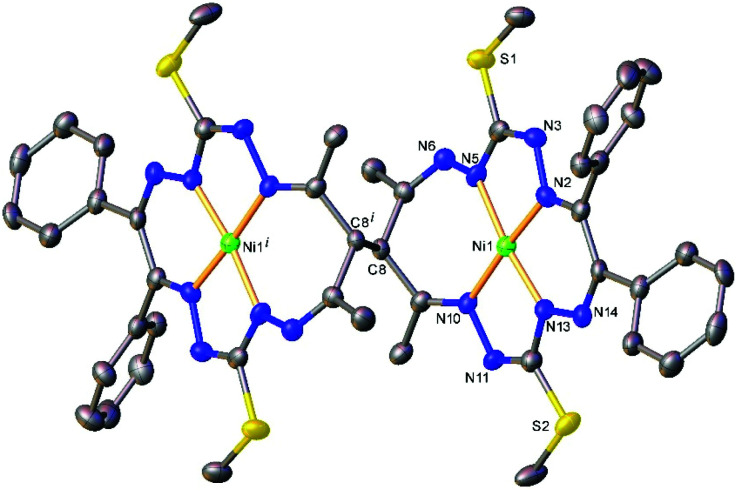
ORTEP view of the molecular structure of 6a (or *meso-S*,*R*-isomer) with atom labeling and thermal ellipsoids at the 50% probability level. H-Atoms are not shown. Selected bond distances (Å) and angles (°): Ni1–N2 = 1.848(5), Ni1–N5 = 1.884(4), Ni1–N10 = 1.882(5), Ni1–N13 = 1.818(4), C8–C8^*i*^ = 1.570(11); N2–Ni1–N5 = 83.0(2), N2–Ni1–N10 = 174.1(2), N5–Ni1–N10 = 102.7(2), N13–Ni1–N2 = 91.3(2), N13–Ni1–N5 = 172.6(2), N13–Ni1–N10 = 83.2(2). Symmetry code: (*i*) 1 − *x*, −*y*, 2 − *z*.

The results of the X-ray diffraction studies of compounds 4a, 4s, 5s and 6a are shown in Fig. 1, 2, 4 and 5, respectively. The selected bond lengths and angles are quoted in the legends of Fig. 1, 2, 4 and 5, while a full list of the metric parameters is collected in Table S1.† The overlay of isomers 4a and 4s is depicted in Fig. 3. All the compounds exhibit molecular crystal structures that are built from the closely related dinuclear complex molecules, [Ni(L^1^–L^1^)Ni] (for 4a and 4s), [Ni(L^2^–L^2^)Ni] (for 5s) and [Ni(L^3^–L^3^)Ni] (for 6a), formed from two chemically identical halves connected through a single C8–C8^*i*^ bond in 4a and 6a, and *via* C8A–C8B bonds in 4s and 5s, respectively. Due to the lack of the symmetry of the seven-membered ring (strictly it has the *C*_1_ local point group symmetry), the dimerization generates two chiral centers at the dimer bonding carbon atoms C8 and C8^*i*^ in *anti*-complexes and C8A and C8B in *syn*-isomers. An inspection of the absolute configurations of the SC-XRD structures of 4a and 4s reveals that they correspond to the diastereomers. 4a represents the (*R*,*S*) or (*S*,*R*)-*meso*-isomer, which forms the same structure due to the binding of two identical monomeric complexes 1. On the other hand, complex 4s crystallized in the centrosymmetric triclinic space group *P*1̄ as a racemic mixture of (*R*,*R*)- and (*S*,*S*)-enantiomers. The complexes 4a and 6a possess crystallographically imposed inversion symmetry, where the center of symmetry is located at the midpoint of the C8–C8^*i*^ bridge, being the reason for their optical inactivity.

The Ni(ii) atom adopts a square-planar coordination geometry being located within the plane formed by four nitrogen atoms of the tetradentate 15-membered octaazamacrocyclic ligand (see, for instance Fig. S1† for 5s). The Ni–N bond distances are very similar for 4a and 4s complexes (see legends to Fig. 1 and 2). Likewise, the other interatomic distances do not differ significantly. Upon the coordination of the macrocyclic ligand, three kinds of chelate rings are formed, namely two five-, one six- and one seven-membered cycle. An increase in the bite angle of up to 20 deg is of note when passing from five- to six- and to seven-membered chelate rings in 4a, 4s, 5s and 6a as it was also recently noticed for starting compounds 1–3.^22^

**Fig. 6:**
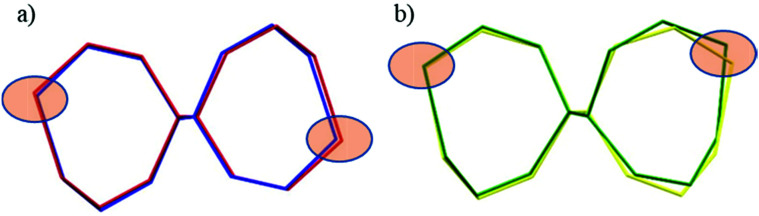
Overlay of the 7-membered chelate rings in dinickel(ii) complexes in capped stick representations: (a) *anti* isomers (*R*,*S*)—blue trace denotes 4a and red trace denotes 6a; (b) *syn* isomers (*S*,*S*)—yellow trace denotes 4s and green trace denotes 5s. Filled ellipsoids indicate the positions of the nickel atoms.

The seven-membered ring is buckled in all complexes studied leading to a characteristic out-of-plane distortion that is facilitated by the non-symmetrical coordination that reduces the strain within the C–C8–C chain. The deviations of atom C8 (or the symmetry-related atom) from the mean planes through the other six atoms in the ring are summarized in Table S2.† It should be noted that an almost perfect overlay of the 7-membered ring conformations in *anti*-isomers (4a and 6a) is observed, while it differs only slightly for one of the two rings in *syn*-isomers (4s and 5s) (*cf.*Fig. 6a and b). The formation of this kind of seven-membered rings is well-documented in the literature,^36,55–57^ even though the number of such examples is scarce. However, their C–C coupling discovered in this work has no literature precedence.

Nevertheless, it is worth noting that pentane-2,4-dione bis(thiosemicarbazones) and pentane-2,4-dione bis(dithiocarbazates) as potential N_2_S_2_ tetradentate ligands form square-planar nickel(ii) complexes and adopt a *C*_2_-symmetric coordination mode with a 5–6–5 chelate ring sequence or a low-symmetric (*C*_1_-symmetric) 4–7–5 chelate ring sequence.^36,56,57^ The linkage isomers with low symmetry are the kinetic products of nickel(ii) complex formation with both types of ligands.^57^ Pentane-2,4-dione bis(dithiocarbazates) were reported to undergo rapid conversion into symmetric species, while pentane-2,4-dione bis(thiosemicarbazonates) were found to be quite inert to linkage isomerization. Therefore, *C*_1_-symmetric nickel(ii) complexes with pentane-2,4-dione bis(thiosemicarbazones) containing a central seven-membered chelate ring are likely to be suitable for the discovery of geometric isomerism in dinickel(ii) complexes with open-chain dinucleating ligands being produced from the nickel(ii) complexes by oxidation reactions *via* the formation of C–C bond between mononuclear species.

The main premise for the appearance of geometrical isomerism in complexes 4–6 is the lack of symmetry of the equal 7-membered chelate rings. The two N–N backbones in the two C–C coupled rings are either in the *anti*-positions to each other as shown in Fig. S2a and S2d† for isomers 4a and 6a or in the *syn*-positions as shown in Fig. S2b and S2c† for isomers 4s and 5s. So, the isomerism in 6 is due to the different relative positions of the N–N units in the two C–C coupled 7-membered chelate rings (Fig. S2d†).

To further confirm the identity of the compounds that have not formed crystals of sufficient quality for the SC-XRD study, ^1^H and ^13^C NMR spectroscopy were used.

### NMR spectroscopy

**Fig. 7:**
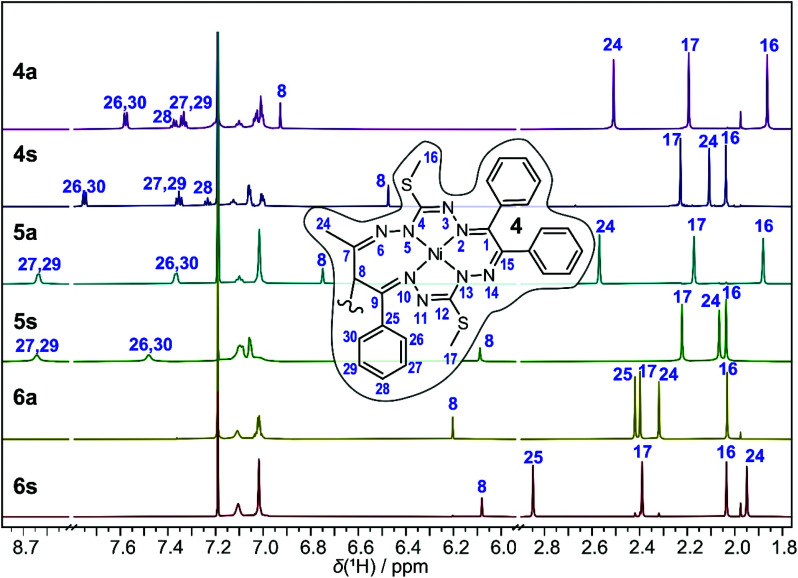
^1^H NMR spectra of the *anti* and *syn* complexes of 4–6 in CDCl_3_. Inset: Single macrocycle of complex 4 with a numbering scheme. Selected proton resonances are assigned with labels of the directly bound carbon. See Tables S3 and S4 (ESI†) for the complete assignment.

The ^1^H NMR spectra of the *anti* and *syn* complexes 4–6 are shown in Fig. 7. As can be seen, the pairs of isomers provide distinctively different resonances. NMR spectroscopy cannot differentiate the enantiomers without the use of chiral reagents but the diastereomers are readily distinguishable. The number of NMR resonances observed in the spectra of 4a and 4s equals the number of resonances for complex 1, and, therefore, agrees with the *C*_i_ point group symmetry of 4a and the two-fold symmetry of 4s in solution, implying that the two macrocycles form magnetically equivalent units.^22^

The ^1^H NMR spectrum of 4a shows three CH_3_ singlets at 1.87, 2.19 and 2.51 ppm that can be assigned to the CH_3_S, CH_3_S and CH_3_C (H24, see the inset in Fig. 7 or Fig. 1, S1† for the numbering scheme) groups, respectively, based on the correlations found in the 2D ^1^H, ^13^C HSQC and HMBC spectra. Due to the presence of intermittent N–N units, the standard sets of ^1^H and ^13^C 2D experiments are insufficient to trace the whole bonding chain of the octaazamacrocycles. We have therefore used DFT calculations at the B3LYP/6-311G* level of theory to aid the assignment of the CH_3_S signals. The DFT calculations predict a lower chemical shift for the H16 thiomethyl protons; thus, the singlet at 1.87 ppm is ascribed to H16 and the resonance signal at 2.19 ppm to H17. The CH protons at the dimeric C–C bond (H8) generate a singlet at 6.93 ppm. The ten protons of the terminal phenyl groups provide a set of overlapping multiplets in the range of 6.97–7.15 ppm. The severe overlap of the resonances in both the ^1^H and ^1^H,^13^C 2D spectra makes the exact attribution of the involved groups problematic, but a tentative assignment is provided in Tables S3 and S4.† The most deshielded protons belong to the R_2_ = Ph group of the original 1,3-diketone moiety; the triplet at 7.34 ppm is assigned to the *m*-(H27,29) protons, the triplet at 7.38 ppm to the *p*-(H28) protons and the doublet at 7.58 ppm to the *o*-(H26,30) protons. The spectrum of 4s features several distinct signal shifts when compared to that of 4a that were found to be diagnostic for the *anti* and the *syn* complexes. Not surprisingly, they involve the groups in the vicinity of the bridging C–C bond. The CH (H8) and CH_3_C (H24) protons in 4s are upfield shifted to 6.46 and 2.03 ppm, respectively. On the other hand, the R_2_ = Ph *o*-(H26,30) protons are downfield shifted to 7.75 ppm. These effects can be rationalized after a detailed inspection of the 3D structures of 4a and 4s (Fig. 1 and 2). In the *anti*- or *meso*-isomer, the CH_3_C (H24) group is found underneath the N10–N11 bond and experiences magnetic deshielding of the π-electrons from the neighbouring C9=N10 and N11=C12 double bonds. In the *syn*-(*R*,*R*/*S*,*S*)-enantiomer, the two macrocycles are twisted and the CH_3_C (H24) groups are pulled away from under the macrocycle, resulting in a decrease of the deshielding. The same geometric rearrangement exerts an inverse effect on the *o*-protons-(H26,30) of the second R_2_ = Ph substituent.

Similar changes in the ^1^H NMR spectrum were found for the 5a, 5s pair (Fig. 7, see Tables S3 and S4† for detail resonance assignment). The signals of the CH (H8) and CH_3_C (H24) protons are found at 6.75 ppm and 2.57 ppm for 5a and at 6.09 ppm and 2.07 ppm for 5s in line with their *anti*/*syn* assignments. The shift in the CH (H8) signal is less pronounced in the spectra of the 6a/6s diastereomers, moving from 6.20 ppm in 6a to 6.08 ppm in 6s. However, the deshielding/shielding effect of the macrocycle π-electrons is readily observed on the R_1,2_ methyl protons, shifting the R_1_ CH_3_C (H24) resonance from 2.32 ppm in *anti*-complex to 1.95 ppm in the *syn*-isomer, and *vice versa*, the R_2_ CH_3_C (H25) singlet from 2.42 ppm in 6a to 2.85 ppm in 6s. The distinct NMR spectra and the correlations between the experimentally determined and DFT-predicted ^1^H and ^13^C chemical shifts (displayed in Fig. S3 and S4†) provide evidence for the presence of the 5a and 6s isomers, while SC-XRD structural confirmation is not available.

### Isomerization (epimerization) kinetics

**Fig. 8:**
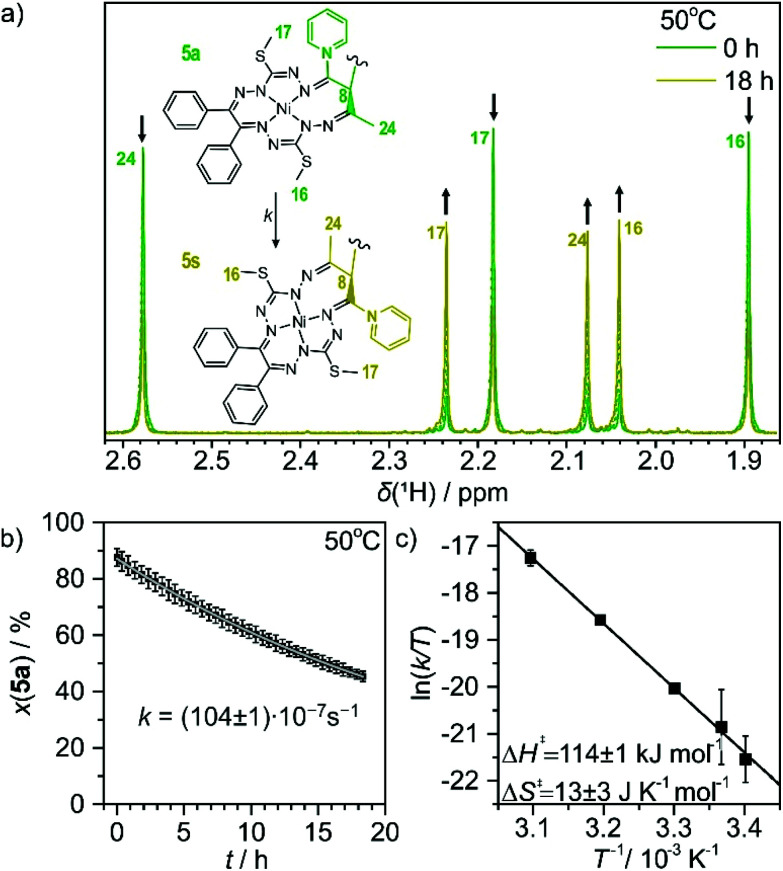
(a) The epimerization of 5a to 5s in CDCl_3_ at 50 °C followed by ^1^H NMR for 18 h. The assignment of relevant resonances is provided in the inset. (b) The decrease of the molar ratio of 5a (black squares) and the first-order rate law fit (gray line) providing the rate constant displayed. (c) Eyring analysis of the temperature dependence of the first-order rate constant and the activation parameters obtained.

Geometrical isomerization in mononuclear Werner-type metal complexes is a well-documented transformation,^58,59^ which can be also induced by electron transfer.^60–62^ During the preparation of pyridyl-bearing compound 5, we observed that the ratio of the *anti*- and *syn*-isomers changed over several weeks at room temperature with a preference for the *syn*-isomer formation. The assignment of all the proton resonances in the available isomers 5a and 5s allowed for the investigation of the kinetics of *anti*–*syn* isomerization (Fig. 8). Structurally, this reaction involves the inversion of chirality at the carbon atom C8 of an individual macrocyclic complex and thus represents an epimerization between the diastereomers.

The transformation rate was monitored by ^1^H NMR spectroscopy in CDCl_3_ at 294, 297, 303, 313 and 323 K at various time intervals. The molar ratios of the decaying 5a and generating 5s (based on resonance signal integration) were plotted against time. The kinetic data are presented in Fig. 8b, Fig. S5 and Table S5.† The recorded ^1^H NMR spectra showed a clean conversion of 5a into 5s without an identifiable intermediate. The process followed the first-order kinetics and the rate constants changed progressively from (1.30 ± 0.03) × 10^−7^ s^−1^ at 294 K to (104 ± 1) × 10^−7^ s^−1^ at 323 K. Eyring analysis of the temperature dependence of the rate constant provided an activation enthalpy of 114 ± 1 kJ mol^−1^ and an activation entropy of 13 ± 3 J K^−1^ mol^−1^ for the isomerization of 5a into 5s. Arrhenius analysis delivered an activation barrier (*E*_a_) of 117 ± 1 kJ mol^−1^ and a frequency factor (*A*) of 8 ± 2 kJ mol^−1^. Essentially identical rate constants and activation parameters were also obtained from an alternative evaluation of the kinetic data, involving the model of a reaction approaching an equilibrium (
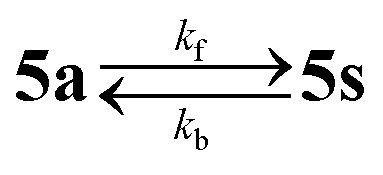
, where *k*_f_ and *k*_b_ denote the forward and backward rate constants; see the ESI†). The activation parameters of isomerization in similar dimeric complexes to the best of our knowledge are absent in the literature. However, the values found here compare well with the activation barrier (*E*_a_) of epimerization in some green tea catechins, which feature some structural similarity to complex 5, namely an aliphatic ring sigma bound to a bulky polyphenolic group.^63^

To verify whether the epimerization reaction is specific solely for the 5a/5s couple, we have also followed the changes in the ^1^H NMR spectra for 6s/6a at 303 K. Interestingly, in this case, the 6s → 6a transformation was observed, indicating that the *anti*-isomer is thermodynamically more stable. The conversion rate is roughly five times slower than that for the inverse process in 5a/5s. Despite the simple kinetics, the epimerization mechanism could be a multistep process. The change from *anti*- or *meso*(*R*,*S*/*S*,*R*) isomer to *syn*-(*R*,*R*/*S*,*S*)-enantiomer requires a switch of chirality at one end of the dimeric bond and likely involves an intermediate with an sp^2^-hydridized carbon atom C8. Such structure could potentially be achieved through deprotonation of the C8H group. Alternatively, a tautomerization within the macrocycle was considered as well (see the ESI section on DFT calculations†).

The starting complexes 1–3 showed quite interesting redox non-innocent electrochemical (EC) and spectroelectrochemical (SEC) behavior.^22,23^ Therefore, it was of interest to study the electrochemical properties of dinickel(ii) complexes 4–6 in comparison to the previously investigated 1–3.

### Electrochemistry and spectroelectrochemistry

The redox behavior of complexes 4–6 in CH_2_Cl_2_/*n*-Bu_4_NPF_6_ was studied by cyclic voltammetry (CV) and square-wave voltammetry (SWV) and compared to that of complexes 1–3 probed under analogous conditions. Electrochemical data and degree of reversibility for each redox event of 1–6 in CH_2_Cl_2_/*n*-Bu_4_NPF_6_ derived from cyclic voltammetry with a Pt working electrode and a scan rate of 100 mV s^−1^ are summarized in Table 1. SWV peak potentials for selected complexes are summarized in Table S6.†

Electrochemical potential data for 1–6 in CH_2_Cl_2_/*n*-Bu_4_NPF_6_*vs*. Fc^+^/Fc^a^

**Table:** 

Monomeric complex	*E* ^ox1^_pa_	*E* ^ox2^_pa_	*E* ^red1^_pc_
1	0.61^r^	0.99^r^	–1.84^r^
2	0.73^r^	1.10^r^	–1.79^r^
3	0.62^r^	1.00^r^	–1.90^r^

**Table:** 

Dimeric complex	*E* ^ox1^_pa_	*E* ^ox2^_pa_	*E* ^ox3^_pa_	*E* ^ox4^_pa_	*E* ^red1,2^_pc_
4a	0.70^q^	0.83^i^	1.22^i^	1.29^i^	–1.71^i^
4s	0.67^i^	0.74^i^	1.24^q^	1.24^q^	–1.77^i^
5s	0.75^i^	0.75^i^	1.24^q^	1.24^q^	–1.49^i^
6a	0.65^i^	0.65^i^	1.18^q^	1.18^q^	–1.78^i^

a Electrochemical data in volts (*E*^ox1^_pa_ – the first anodic peak potential, *E*^ox2^_pa_ – the second anodic peak potential, *E*^ox3^_pa_ – the third anodic peak potential, *E*^ox4^_pa_ – the fourth anodic peak potential, *E*^red1^_pc_ – the first cathodic peak potential, and *E*^red1,2^_pc_ – nearly two electron transfer cathodic event; r – reversible, q – quasireversible or i – irreversible electrochemical process). Pt working electrode, scan rate 100 mV s^−1^.

**Fig. 9:**
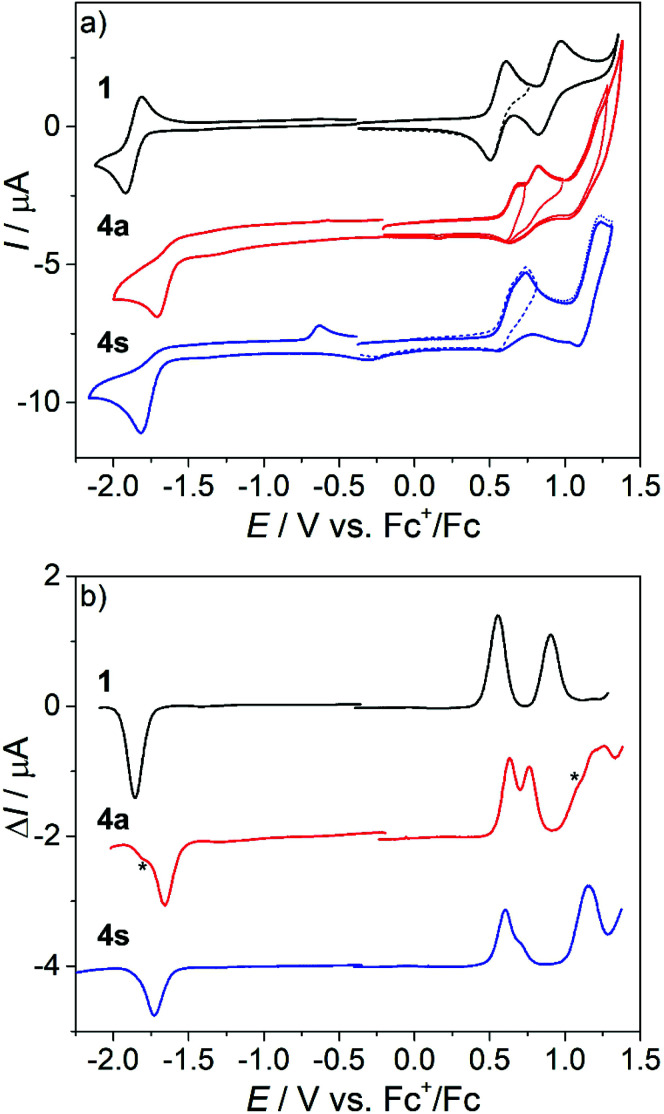
Cyclic voltammograms (CVs) of (a) 1 (black lines), 4a (red lines) and 4s (blue lines) at the scan rate of 100 mV s^−1^. (b) Square-wave voltammograms (SWVs) of 1, 4a and 4s (recorded with 50 mV step amplitude, 5 mV step increment and 40 ms pulse duration) using a Pt disk working electrode in CH_2_Cl_2_/*n*-Bu_4_NPF_6_. The signals marked with an asterisk (*) are due to minor contaminations of the isomer 4a with isomer 4s.

The differences in the redox behavior of the mononuclear compound 1 and the corresponding dinuclear complexes 4a and 4s are shown in Fig. 9a. In contrast to 1, where two reversible oxidation peaks were observed (Fig. 9, black traces), the first anodic peak of the corresponding dimer 4a is shifted to a higher value and its electrochemical reversibility is lower (Fig. 9, red traces). The second, third and fourth oxidation events are irreversible. In the CV of the *syn*-dimer 4s, the first two oxidations are irreversible and their signals appear at mutually closer spaced peak potentials. The third and the fourth oxidations are quasireversible two-electron events. As already confirmed in our previous studies on mononuclear species 1–3, the first anodic oxidation step was much less dependent on the macrocycle substitution pattern suggesting a contribution of the Ni(ii) ion to the redox behavior.^22^ The second oxidation peak was more dependent on the substitution pattern indicating a ligand-centered second electron transfer in the case of mononuclear complexes and, therefore, this can be also applied to the third and the fourth anodic redox events of the corresponding dimeric complexes. Note that since the dinickel(ii) complexes are built up from two equivalent nickel(ii) octaazamacrocyclic units, the first and the second anodic peaks each correspond to a single-electron transfer process at each individual macrocycle. This was confirmed by the cyclic voltammetry of mononuclear and dinuclear complexes with identical molar concentrations in the presence of ferrocene as an internal standard (Fig. S6†). The potential separation between the first two oxidation peaks (130 mV in 4a and 70 mV in 4s) can be considered as a measure of the electron interaction in the oxidized macrocycles.^64^ This is clearly stronger in the *anti*4a complex configuration, while in 4s the individual mononuclear complexes can be regarded as almost independent units (note that even if there is no interaction between two equivalent redox centres in a dimer, their redox potentials will still differ due to the statistical factor by 35.6 mV).^65^

**Fig. 10:**
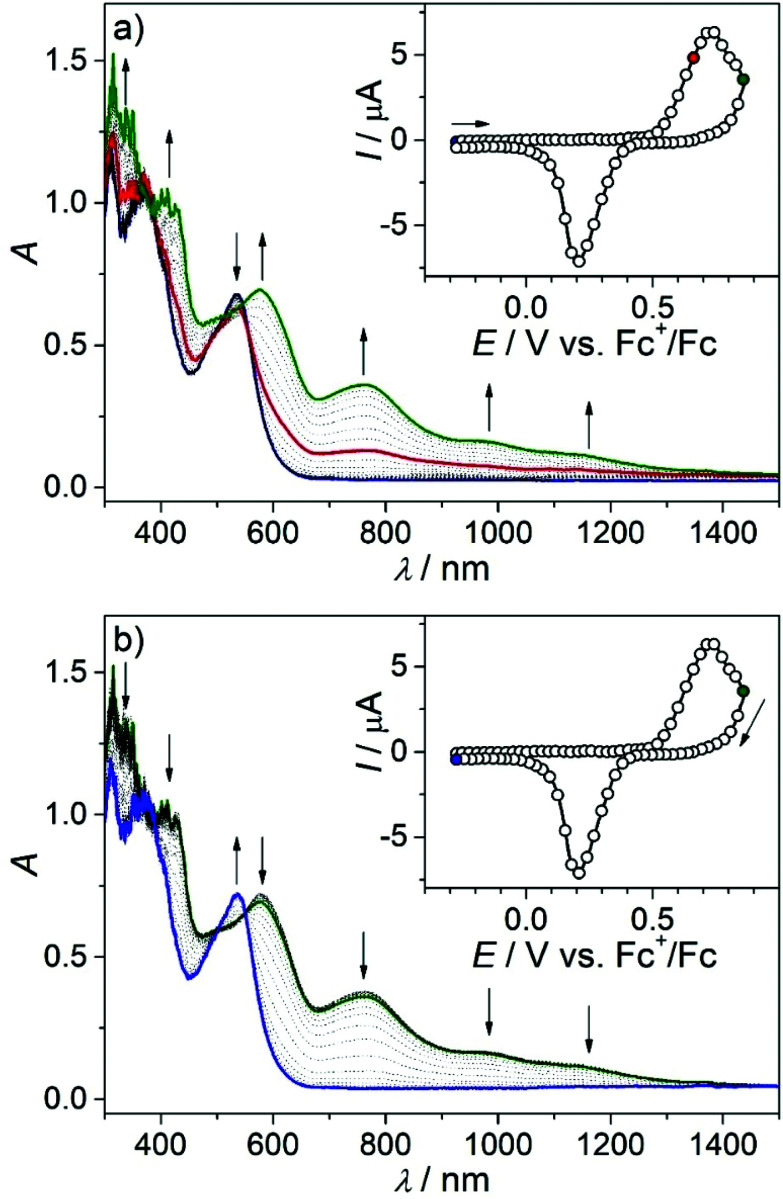
UV–vis–NIR spectra measured in the region of the first and second anodic peaks during the (a) forward and (b) reverse scans of 4a in CH_2_Cl_2_/*n*-Bu_4_NPF_6_ under an argon atmosphere using a Pt working electrode and a 3 mV s^−1^ scan rate. Insets show the corresponding CV and colored mark potential at which the highlighted spectra were taken.

Upon scan reversal in the CV covering the first and/or the first and the second oxidations, a new strongly shifted cathodic peak arises at *ca*. 0.19 V *vs*. Fc^+^/Fc for 4a and at −0.3 V *vs*. Fc^+^/Fc for 4s. A large peak-to-peak separation suggests a significant structural difference between the initial and oxidized states as a result of chemical follow-up reactions. Interestingly, a second consecutive CV scan is very similar to the first, indicating the chemical reversibility of the follow-up events. The redox behavior involving this reversibility can be explained by the electrochemical eCeC square scheme.^64^ The electrochemical behavior of 4a upon the abstraction of the first two electrons is shown in Fig. S7,† along with the corresponding digitally simulated voltammograms. Briefly, the one-electron oxidized 4a^+^ undergoes conversion into a new more thermodynamically stable form 4a*^+^ (involving yet unidentified structural changes) that is chemically reversible upon the back scan reduction (4a*^+^ + e^−^ → 4a* → 4a). The 4a* form features a less positive oxidation potential giving rise to the cathodically shifted re-reduction peak at 0.19 V *vs*. Fc^+^/Fc. The intensity of this peak increases at low scan rates (see Fig. S7b† and the inset in Fig. 10), when the rate of 4a^+^ reduction in the CV back scan becomes competitive or slower than that of the chemical transformation. The second oxidation peak, corresponding to the removal of the next electron from 4a^+^, is also scan rate dependent and its intensity diminishes at slow scan rates following the same reasoning. An analogous mechanism can be likely proposed for 4s with appropriate adjustments of a few parameters (redox potentials and rates of chemical transformations).

The redox behavior of dinickel(ii) complexes 5s and 6a in the anodic part of the CV was very similar to that of 4s (Fig. S8†). The same holds for the reduction in the cathodic region, where one irreversible two-electron transfer peak is seen at the scan rate of 100 mV s^−1^. A strongly shifted reoxidation peak appears at around −0.6 V *vs*. Fc^+^/Fc for all investigated dinickel(ii) complexes both in CH_2_Cl_2_ and CH_3_CN (see Fig. S6 and S8b†), again indicating a strong structural reorganization upon the 2e-reduction. Note that this was not the case for the mononuclear nickel(ii) complexes 1–3. As in the 1–3 series, the most negative reduction potential was observed for 6a (Chart 1) with R_1_ = R_2_ = Me, while more positive reduction potential was found for 5s with R_1_ = Me and R_2_ = Py (Chart 1). An opposite trend was noticed in the anodic part with the lowest oxidation potential being observed for 6a. Note that although the redox processes in the region of the first electron transfer are electrochemically irreversible, the first and the second consecutive scans are almost identical (see Fig. S6 and S8, ESI†). This is consistent with the reversible chemical changes upon redox cycling accompanied by a strong structural change of differently charged states.

The *in situ* UV–vis–NIR SEC of 4a in the region of the first oxidation event showed a simultaneous decrease in the initial optical band at 534 nm and the evolution of a new absorption band at 770 nm with shoulder absorptions reaching the NIR range (Fig. 10a, red trace). Upon extending the scan to the potential range including the second oxidation, new bands at 419 nm, 576 nm, 763 nm (shoulders at 980 nm and 1150 nm) were observed (Fig. 10a green trace).

Note that due to the larger IR drop in the spectroelectrochemical cell, the two closely spaced oxidations are hardly distinguishable in the corresponding CV (Fig. 10a inset) and the optical bands of the oxidation products might be similar. Upon scan reversal at 3 mV s^−1^, a shifted reduction peak is observed indicating that the accompanying peak has to be assigned to the product of the chemical transformation following the oxidation of 4a (the 4a*^+^ species in Fig. S7a†). An almost full recovery of the initial optical spectrum is seen for 4a in a thin-layer cell (Fig. 10b), confirming the chemical reversibility of the reactions upon reduction of the oxidized complex. A similar spectroelectrochemical response was found for 4s and 6a at the first anodic two-electron oxidation peak (Fig. S9†), while for 5s, a markedly less reversible behavior was observed (Fig. S10†).

Our recent studies of 1–3 confirmed that the interaction of the CH_2_ group in the macrocycle with dimethylformamide (DMF) results in the deprotonation of the macrocycle in 1–3.^23^ The optical spectra of the dinickel(ii) complex isomers 4a and 4s are very similar and together with 5s and 6a resemble the spectra of the corresponding mononuclear species 1–3 in CH_2_Cl_2_ and CH_3_CN. The absorption intensity is roughly doubled (when comparing the spectra of dimers to monomers at the same concentration) as expected for complexes carrying two identical weakly interacting chromophores (Fig. S11†).

Note that the monomers show different spectra in DMF than in CH_2_Cl_2_ and CH_3_CN because of the deprotonation in DMF as illustrated for 4a and 1 in Fig. S12.† This might be due to the presence of a small amount of a strong base NHMe_2_ as a decomposition product of DMF. The differences are most apparent in diluted solution (see the blue trace in Fig. S12†). For the dinickel(ii) complexes, the spectra were similar in all solvents used.

In order to get a deeper insight into the described geometric isomerism and rationalize the spectroelectrochemical behavior of the dinickel(ii) complexes, as well as to identify the locus of the reported electron-transfer events, theoretical DFT calculations were performed.

### Theoretical studies

In this section, we first focus on the *syn*/*anti* isomer thermodynamic preference of the studied compounds. In the following, the single and two electron oxidized species of 4 are investigated (including a frontier orbitals comparison) and their electronic transitions are compared to the experimentally obtained UV–vis spectra. Thereafter, several structural variants of the neutral species of 4 are mentioned in relation to isomerization.

The optimized geometries of 4s, 4a, 5s and 6a compare well with the structures established by SC-XRD (see Table S1†). The total B3LYP/6-311G* energies of the relevant *syn* and *anti*-species are compiled in Table S7.† The initial neutral forms are found to be closed-shell singlets. It should be noted that the *syn*-isomer 4s is the energetically preferred one (the Boltzmann ratio is 11.3% of 4a and 88.7% of 4s at 298.15 K and the energy difference is 5.1 kJ mol^−1^) but the actual energetics concerning oxidation appear very similar when comparing the *syn* and *anti*-forms of the dinickel(ii) complex 4 (see Table S7†). In the case of 5, 5s is also the energetically preferred structure over 5a, by 4.1 kJ mol^−1^ (see Table S7†) and hence the respective 298.15 K Boltzmann ratios are 84 and 16% and in accord with the experimental findings. On the other hand (in contrast with 4a/4s and 5a/5s), 6a is lower in energy than 6s by 2.4 kJ mol^−1^ (see Table S7†) and the respective 298.15 K Boltzmann ratio is 72.5 and 27.5%. This is also in line with the ^1^H NMR isomerization experiment described previously.

**Fig. 11:**
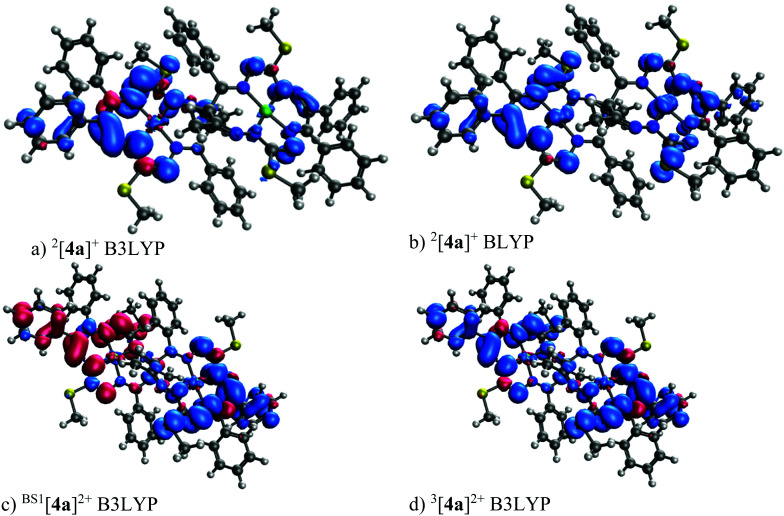
DFT 6–311G* spin density (isovalue 0.001 e bohr^−3^) of ^2^[4a]^+^ and [4a]^2+^.

The 1e-oxidized and 1e-reduced species ^2^[4a]^+^ and ^2^[4a]^−^ are doublets. The 2e-oxidized and 2e-reduced species prefer the broken symmetry singlet spin states, with one unpaired electron on each macrocyclic unit. The *J*-coupling values of [4a]^2+^ and [4a]^2−^ are 0.87 and 4.62 cm^−1^, respectively (an antiparallel spin orientation on the individual macrocycles). The *J*-coupling value of [4s]^2+^ is 1.10 cm^−1^. The spin density distribution calculated for the primarily formed ^2^[4a]^+^ is shown in Fig. 11. The B3LYP spin populations (0.008 and 0.000) on the Ni atom are very low (see Table S7†) as can be assumed from the spin density (see Fig. 11a). A larger spin population (0.122 and 0.082) on Ni is found for the pure-GGA (general gradient approximation) BLYP functional (see Fig. 11b). An even larger spin population (0.151 and 0.145) is found on the central atom for the OPBE functional. The broken symmetry singlet ^1^[4a]^2+^ and triplet ^3^[4a]^2+^ spin distributions are shown in Fig. 11c and d, respectively. These spin densities agree with that of [4a]^2+^ shown in Fig. 11a. The B3LYP/6-311G* frontier orbital diagram of ^1^[4a]^0^ is shown in Fig. S13a.† Importantly, the frontier orbitals of ^1^[4a]^0^ are almost exclusively double degenerate—once in phase and once in anti-phase with respect to the inversion center (compare HOMO−1 to HOMO and LUMO to LUMO+1 of ^1^[4a]^0^ in Fig. S13a†). In contrast, the B3LYP/6-311G* frontier orbital diagram of ^2^[4a]^+^ (Fig. S13b†) indicates that the α and β HOMO−2, HOMO−1 and HOMO are the paired orbitals of ^2^[4a]^+^, while α HOMO−3 is the open-shell (SOMO) that closely resembles the shape of the β LUMO and spin density (see Fig. 11a).

The TD transitions of ^1^[4a]^0^ and ^1^[4s]^0^ as well as the one- and two-electron oxidized species are shown in Fig. S14.† The oxidized species ^2^[4]^+^ shows an absorption edge shift to larger wavelengths when compared to the neutral one ^1^[4]^0^, *i.e.*, the oxidized species shows several transitions in the range of 500–800 nm. The two electron oxidized species [4]^2+^ has these 500–800 nm range transitions that are more intense than those in line with the measured UV–vis spectra (compare to Fig. 10).

Additional structures related to the redox transformations, rotational isomers and tautomerization of 4, see *e.g.* Fig. S15, S16 and Table S8, are discussed in the ESI section on DFT calculations.†

### Catalytic studies

**Scheme 1:**
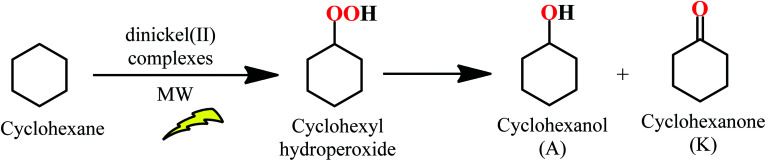
MW-assisted oxidation of cyclohexane to cyclohexyl hydroperoxide, cyclohexanol and cyclohexanone with aqueous TBHP catalyzed by dinickel(ii) complexes 4a, 4s, 5s and 6a/6s under solvent-free conditions.

The catalytic activities of Ni(ii) complexes 4a, 4s, 5s and 6a/6s were investigated towards the neat MW-assisted cyclohexane oxidation under mild conditions using *tert*-butyl hydroperoxide (TBHP, 70% aq. solution) as the oxidant (Scheme 1). Microwave (MW) irradiation was used as an alternative energy source over the conventional heating method. This source of energy has proved to significantly enhance the catalytic activity of these systems, promoting the product yield.^66,67^

**Fig. 12:**
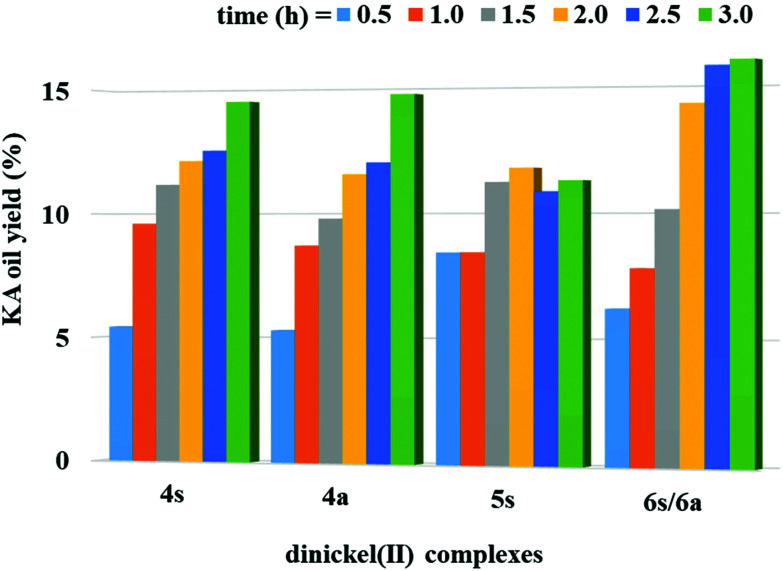
Total yield of cyclohexanol and cyclohexanone (KA oil) obtained by MW-assisted oxidation of cyclohexane with THBP catalyzed by the dinickel(ii) complexes 4s, 4a, 5s and 6a/6s (10 μmol) at 100 °C. Reaction conditions: cyclohexane (2.5 mmol), 70% aq. TBHP (5.0 mmol), 10.0 μmol of the catalyst [4a, 4s, 5s and 6 (as a mixture of 6s and 6a)] under homogeneous and solvent-free conditions. Total (cyclohexanol + cyclohexanone) turnover number was calculated as the moles of products (KA oil) per mol catalyst (dinuclear complex) determined by GC analysis (upon treatment with PPh_3_).

The studied dinickel(ii) complexes performed effectively as catalysts for the above reaction, leading to cyclohexanol and cyclohexanone (KA oil). The selected reaction conditions were in line with those reported in our previous study for the complexes 1–3^22^ [100 °C and 3 h of low power (25 W) MW irradiation] under solvent-free conditions. For the dinickel(ii) complexes, yields of KA oil in the range of 11–16% were obtained, upon using a low catalyst load (Fig. 12 and Table 2), with moderate turnover number (TON) values ranging from 28 to a maximum of 40 for compound 6. Blank experiments, in the absence of 4a, 4s, 5s and 6a/6s, were performed under the optimized reaction conditions but no cyclohexane conversion was detected. Experiments with NiCl_2_·6H_2_O were performed using the optimized reaction conditions found for 4–6. The obtained KA oil yield under such conditions was 0.8%.

**Table:** Selected data for the MW-assisted catalytic peroxidative oxidation of cyclohexane in MeCN catalyzed by the dinickel(ii) complexes^a^

Catalyst	TON^b^	Yield^c^ (%)		
		A	K	Total
4s	36	10.2	4.3	14.5
4a	37	9.8	5.0	14.8
5s	28	7.3	4.0	11.3
6	40	9.7	6.3	16.0

a Reaction conditions: cyclohexane (2.5 mmol), 70% aq. TBHP (5.0 mmol), 10.0 μmol of the catalyst under homogeneous and solvent-free conditions, 100 °C, 3 h of reaction.

b Total (cyclohexanol (A) + cyclohexanone (K)) turnover number: moles of products per mol catalyst (dinuclear complex) determined by GC analysis (upon treatment with PPh_3_).

c Moles of product/100 moles of cyclohexane.

**Scheme 2:**
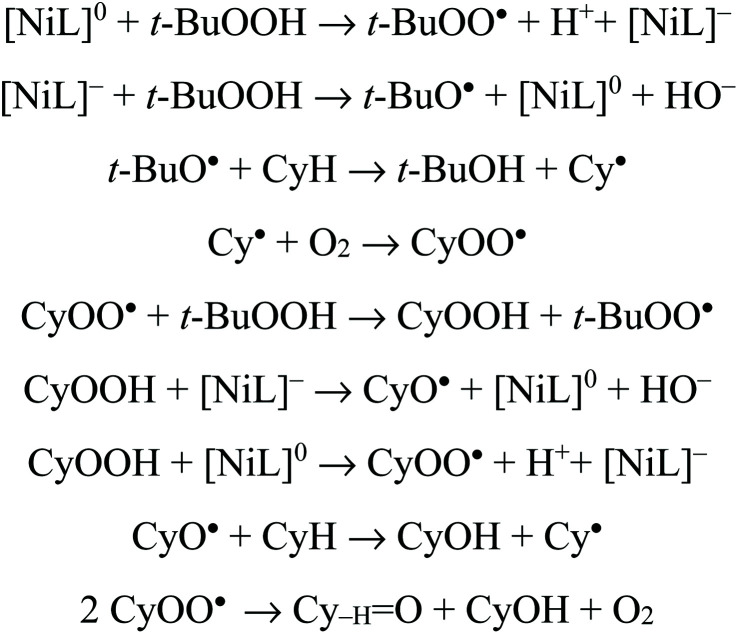
Proposed reaction mechanism for the oxidation of cyclohexane catalyzed by complexes 4a, 4s, 5s and 6a/6s, where NiL formally represents one half of the corresponding molecule.

High selectivity was observed towards the formation of KA oil (cyclohexane and cyclohexanone mixture) with these complexes as cyclohexanol and cyclohexanone were the only products detected under these assayed conditions, thus revealing a selective oxidation system. After 3 h of reaction, the increase in the oxidation products was considered negligible. The limiting factor is catalyst deactivation as TBHP is still detected at the end (after 3 h) of the catalytic assays. The obtained yields are slightly lower than those previously reported^22^ for the corresponding monomeric Ni(ii) complexes that ranged from 7 to 23% for KA oil yield under the same MW-assisted reaction conditions. The experimental results allowed us to confirm the formation of cyclohexyl hydroperoxide as a primary product, as observed in other systems,^22^ following a method proposed by Shul'pin,^68–71^ with a significant increase in the detected cyclohexanol amount after the addition of triphenylphosphine to the reaction mixture (due to the reduction of CyOOH to CyOH by PPh_3_) and a decrease in the amount of cyclohexanone. It is assumed that the reaction should proceed through a free radical mechanism, as previously discussed in ref. 22 (see Scheme 2). The non-innocent reduction and oxidation characters of the ligand are believed to assist the redox processes involved with TBHP in cooperation with the metal that in such a way only needs to undergo a shorter variation in electron density.[NiL]^0^ + *t*-BuOOH → *t*-BuOO˙ + H^+^ + [NiL]^−^[NiL]^−^ + *t*-BuOOH → *t*-BuO˙ + [NiL]^0^ + HO^−^*t*-BuO˙ + CyH → *t*-BuOH + Cy˙Cy˙ + O_2_ → CyOO˙CyOO˙ + *t*-BuOOH → CyOOH + *t*-BuOO˙CyOOH + [NiL]^−^ → CyO˙ + [NiL]^0^ + HO^−^CyOOH + [NiL]^0^ → CyOO˙ + H^+^ + [NiL]^−^CyO˙ + CyH → CyOH + Cy˙2CyOO˙ → Cy_–H_=O + CyOH + O_2_

The lower performances presented by the dinuclear complexes 4s, 4a, 5s and 6a/6s in comparison with those found for their monomers are in disagreement with the anodic shifts of the reduction potentials observed by cyclic voltammetry for the dimeric species (see above) relative to the corresponding mononuclear complexes 1–3.^22^ It appears that the higher steric effects of the dimeric compounds in comparison with the monomeric ones are responsible for the lower catalytic activity of the former. In fact, the steric hindrance of the catalysts (dinuclear species) may affect their interaction with TBHP (also a bulky molecule), which is believed to be the first step of the abovementioned radical mechanism: the Ni-catalysed formation of the *t*-BuOO˙ and *t*-BuO˙ radicals. This is a key step for the occurrence of C–H abstraction from the cyclohexane, leading to the formation of cyclohexyl radicals and the beginning of cyclohexane conversion. However, the lower activity of the dimeric complexes is consistent with the anodic shifts of the oxidation potential in comparison with the mononuclear analogues, thus being more resistant to oxidation than the latter.

The present yields are higher than (or comparable to) those reached in the industrial process (*ca*. 8%, to assure a good selectivity),^46,48^ although lower than other previously reported yields.^72–74^ Nevertheless, our catalysts compare favorably to another recently reported system based on the dinickel(ii) complex [Ni_2_(L^1^)_2_(μ_2_-N_3_)_2_(CH_3_OH)_2_]·CH_3_OH, where HL^1^ is 1-((2-hydroxyethylimino)methyl)naphthalen-2-ol, for the oxidation of cyclohexane, as it was found to be inactive in this case.^75^ Moreover, our results are quite similar to those obtained by a set of related Ni(ii) complexes of N-heterocyclic carbene ligands (NHC), *trans*-[X_2_Ni(NHC)_2_] (X = Cl and I), achieving product yields of about 15% for cyclohexane oxidation, although our system proceeds under milder conditions.^76^

## Conclusions

This work provides a synthetic entry to unprecedented diastereomeric dinickel(ii) bis(octaazamacrocyclic) complexes consisting of two redox non-innocent 15-membered octaazamacrocyclic nickel(ii) complexes with a chelate ring size pattern 7–5–6–5 joined *via* a carbon–carbon single bond. The use of 1,3-diketones for the template ring-closure reaction and the assembly of mononuclear prochiral nickel(ii) complexes 1–3, as well as the presence of a low-symmetry seven-membered chelate ring are the main prerequisites for the discovered stereoisomerism in dinickel(ii) complexes 4–6. The formation and isolation of three pairs of diastereomers were confirmed by ^1^H NMR spectroscopy, ESI mass spectrometry and SC-XRD measurements. The complexes underwent slow isomerization in chloroform at room temperature and this transformation was monitored by ^1^H NMR spectroscopy. The conversion of 5a into 5s with an activation enthalpy of 114 ± 1 kJ mol^−1^ and activation entropy of 13 ± 3 J K^−1^ mol^−1^ has been confirmed experimentally. We believe that low symmetry nickel(ii) complexes with pentane-2,4-dione bis(thiosemicarbazones) with a 4–7–5 chelate ring sequence, which were found to be inert to linkage isomerization,^36^ may also be suitable for the discovery of geometric isomerism by following the same kind of oxidation chemistry, but in this case with chiral open-chain dinucleating ligands.

The cyclic voltammetry of the mononuclear and dinuclear nickel(ii) complexes confirmed that the first and the second anodic peaks correspond to the single-electron transfer processes on separate macrocycles and are followed by reversible chemical follow-up transformations. The potential separation between the first two oxidation peaks found for the isomers 4a/4s implies electronic interaction between the oxidized macrocycles. The interaction between the macrocyclic units was evident for the *anti*-isomers, while the *syn*-isomer individual mononuclear complexes can be regarded as almost independent moieties. The absorption intensity in the optical spectra of 4–6 is roughly doubled, compared to that of monomers, as expected for complexes carrying two identical weakly interacting chromophores.

As for mononuclear complexes 1–3, DFT calculations on 4a have shown that the one-electron oxidized and one-electron reduced species are doublets with a low spin population on both Ni atoms and a dominant spin population on the corresponding macrocyclic ligand. Furthermore, one-electron oxidation and one-electron reduction produce species with one unpaired electron on each macrocyclic unit which are coupled antiferromagnetically with calculated *J* values of 0.87 and 4.62 cm^−1^ for [4a]^2+^ and [4a]^2−^, correspondingly. The *anti*/*syn* preference for 4–6 is well reproduced in the DFT calculations. Isomerization can be activated when the bridging carbon atom (C8,C8^*i*^) becomes deprotonated.

The studied compounds performed as catalysts for the MW-assisted and solvent-free oxidation of cyclohexane, affording selectively cyclohexanol and cyclohexanone in moderate yields (up to 16%). Even though favorable anodic shifts of the reduction potential were observed for 4–6 when compared to 1–3, the lower catalytic efficacy of the dinickel(ii) complexes is likely due to the increase in the steric effects. In comparison with the industrial process, the current system operates under milder conditions (lower temperature) using a cleaner heat source and can achieve higher yields with the preservation of high selectivity. However, the oxidant (*tert*-butyl hydroperoxide) is not so environmentally friendly as dioxygen which is used in the industrial process.

## Experimental

Complexes 1–3 were synthesized as described in the literature.^22^

### Synthesis of complexes

#### [Ni^II^(L^1^–L^1^)Ni^II^] (4a and 4s)

To a solution of 1 (400 mg, 0.68 mmol) in CHCl_3_ (20 mL) was added a solution of FeCl_3_·6H_2_O (400 mg, 1.5 mmol) in ethanol (4 mL) and pyridine (0.5 mL, 6.3 mmol). The reaction mixture was stirred at room temperature for 72 h. Then the volatiles were removed under reduced pressure and the residue was thoroughly washed with water and ethanol and dried *in vacuo* overnight. Yield: 340 mg, 85%. Anal. Calcd for C_56_H_50_Ni_2_N_16_S_4_ (*M*_r_ = 1192.75), %: C, 56.39; H, 4.23; N, 18.79; S, 10.75. Found, %: C, 56.05; H, 4.16; N, 18.55; S, 10.65%. The 4a/4s isomer ratio in the reaction mixture was estimated to be 45/55 ± 3% according to the ^1^H NMR spectra (see below). The product was separated into isomers on silica by using CHCl_3_ and CHCl_3_ containing 1% ethanol v/v as eluent. Two main red fractions were collected. The first isomer 4a (*R*_f_*ca*. 0.8) was isolated as red microcrystals, washed with ethanol and dried *in vacuo*. Yield: 150 mg, 37%. The second isomer 4s (*R*_f_*ca*. 0.6) was also isolated as red microcrystalline product. Yield: 190 mg, 48%. ^1^H NMR (500 MHz,CDCl_3_), *δ*, ppm for 4a: 1.87 (s, 6H; SCH_3_), 2.19 (s, 6H; SCH_3_), 2.51 (s, 6H; CCH_3_), 6.93 (s, 2H; CH), 7.01–7.40 (m, 26H; Ph), 7.58 (d, *J* = 7.1 Hz, 4H; Ph); ^13^C NMR (125 MHz, CDCl_3_), *δ*, ppm: 13.92 (2SC̲H_3_), 14.57 (2SC̲H_3_), 28.11 (2CC̲H_3_), 50.88 (2C̲H), 126.90 (2C4 of Ph), 127.60 (8C3 of Ph), 127.71 (4s3 of Ph), 127.78 (2C4 of Ph), 129.30 (4s2 of Ph), 129.60 (4s2 of Ph), 129.75 (2C4 of Ph), 129.86 (4s2 of Ph), 136.74 (2C1 of Ph), 138.56 (2C̲Ph), 140.82 (2C̲CH_3_), 140.93 (2C̲Ph), 167.47 (2C̲Ph), 170.37 (2C̲SCH_3_), 174.67 (2C̲SCH_3_)—two quaternary C were not found due to the poor signal-to-noise ratio. ^1^H NMR (500 MHz,CDCl_3_), *δ*, ppm for 4s: 2.04 (s, 6H; SCH_3_), 2.11 (s, 6H; CCH_3_), 2.23 (s, 6H; SCH_3_), 6.47 (s, 2H; CH), 7.00–7.28 (m, 22H; Ph), 7.35 (t, *J* = 7.7 Hz, 4H; Ph) 7.75 (d, *J* = 8.3 Hz, 4H; Ph); ^13^C NMR (125 MHz, CDCl_3_), *δ*, ppm: 13.57 (2SC̲H_3_), 14.54 (2SC̲H_3_), 26.79 (2CC̲H_3_), 48.07 (2C̲H), 126.90 (2C4 of Ph), 127.30 (8C3 of Ph), 127.45 (2C4 of Ph), 127.70 (4s3 of Ph), 129.30 (4s2 of Ph), 129.45 (4s2 of Ph), 129.55 (4s2 of Ph), 129.90 (2C4 of Ph), 138.16 (2C1 of Ph), 138.20 (2C1 of Ph), 138.37 (2C̲Ph), 139.38 (2C̲Ph), 144.25 (2C̲CH_3_), 162.73 (2C̲Ph), 168.90 (2C̲SCH_3_), 174.00 (2C̲SCH_3_)—one quaternary C was not found due to the poor signal-to-noise ratio. The positive ion ESI MS for 4a and 4s: *m*/*z* 1193 [M + H]^+^, 1215 [M + Na]^+^.

#### [Ni^II^(L^2^–L^2^)Ni^II^]·H_2_O (5s·H_2_O)

To a solution of 2 (400 mg, 0.68 mmol) in CHCl_3_ (20 mL) was added a solution of FeCl_3_·6H_2_O (400 mg, 1.5 mmol) in ethanol (4 mL) and pyridine (0.5 mL, 6.3 mmol). The mixture was stirred at room temperature for 48 h. Then the volatiles were removed under reduced pressure, the residue was thoroughly washed with water and ethanol and dried *in vacuo* overnight. Yield: 350 mg, 87.5%. Anal. Calcd for C_54_H_48_Ni_2_N_18_S_4_·H_2_O (*M*_r_ = 1212.75): C, 53.48; H, 4.16; N, 20.79; S, 10.58. Found, %: C, 53.42; H, 4.03; N, 20.68; S, 10.48. The 5a/5s isomer ratio in the reaction mixture was estimated to be 8/92 ± 1% according to the ^1^H NMR spectra (see below). The product was separated on silica column by using as eluent CHCl_3_ and CHCl_3_ containing 10% ethyl acetate v/v to give two dark-red species: the main complex 5s (*R*_f_*ca*. 0.1) (340 mg, 85%) and the second one 5a (*R*_f_*ca*. 0.12) (10 mg, 2.5%). ^1^H NMR (500 MHz, CDCl_3_), *δ*, ppm for 5a: 1.89 (s, 6H; SCH_3_), 2.17 (s, 6H; CCH_3_), 2.57 (s, 6H; SCH_3_), 6.75 (s, 2H; CH), 7.00–7.15 (m, 20H; Ph), 7.37 (d, *J* = 1.5 Hz, 4H; Py), 8.64 (d, *J* = 1.5 Hz, 4H; Py); ^13^C NMR (125 MHz, CDCl_3_), *δ*, ppm: 13.82 (2SC̲H_3_), 14.47 (2SC̲H_3_), 28.33 (2CC̲H_3_), 49.44 (2C̲H), 122.37 (4s3 of Py), 126.97 (2C4 Ph), 127.46 (4s3 of Ph), 127.97 (2C4 of Ph), 129.62 (4s2 of Ph), 138.01 (2C̲Ph), 139.00 (2C̲CH_3_), 139.96 (2C4 of Py), 141.68 (2C̲Ph), 149.61 (4s2 of Py), 162.85 (2C̲Py), 171.66 (2C̲SCH_3_), 176.07 (2C̲SCH_3_)—two quaternary C were not found due to the poor signal-to-noise ratio. ^1^H NMR (500 MHz, CDCl_3_), *δ*, ppm for 5s: 2.04 (s, 6H; SCH_3_), 2.07 (s, 6H; CCH_3_), 2.22 (s, 6H; SCH_3_), 6.09 (s, 2H; CH), 7.00–7.13 (m, 20H; Ph), 7.48 (d, *J* = 3.5 Hz, 4H; Py), 8.64 (d, *J* = 3.5 Hz, 4H; Py); ^13^C NMR (125 MHz, CDCl_3_), *δ*, ppm: 13.98 (2SC̲H_3_), 14.79 (2SC̲H_3_), 27.20 (2CC̲H_3_), 46.74 (2C̲H), 123.06 (4s3 of Py), 126.97 (2C4 Ph), 127.01 (4s3 of Ph), 127.37 (4s2 of Ph), 127.76 (2C4 of Ph), 130.11 (4s2 of Ph), 134.89 (2C1 of Ph), 138.27 (2C̲Ph), 141.54 (2C̲Ph), 142.10 (2C1 of Ph), 143.21 (2C̲CH_3_), 146.56 (2C4 of Py), 149.28 (4s2 of Py), 159.60 (2C̲Py), 170.23 (2C̲SCH_3_), 176.24 (2C̲SCH_3_). Positive ion ESI MS for 5a and 5s: *m*/*z* 1195 [M + H]^+^, 1217 [M + Na]^+^.

#### [Ni^II^(L^3^–L^3^)Ni^II^]·2H_2_O (6·2H_2_O)

To a solution of 3 (200 mg, 0.38 mmol) in CHCl_3_ (15 mL) was added a solution of FeCl_3_·6H_2_O (220 mg, 0.83 mmol) in ethanol (2.2 mL) and pyridine (0.3 mL, 0.34 mmol). The mixture was stirred at room temperature for 72 h. Then the volatiles were removed under reduced pressure, the residue was thoroughly washed with water and ethanol and then dried *in vacuo* overnight. Yield: 175 mg, 85%. Anal. Calcd for C_46_H_46_Ni_2_N_16_S_4_·2H_2_O (*M*_r_ = 1104.65), %: C, 50.02; H, 4.56; N, 20.29; S, 11.61. Found: C, 49.97; H, 4.21; N, 19.95; S, 11.30. The 6a/6s isomer ratio in the reaction mixture was estimated to be 28/72 ± 2% according to the ^1^H NMR spectra. Further separation performed by column chromatography (SiO_2_, eluent CHCl_3_, CHCl_3_-3% ethyl acetate v/v) resulted in two red products 6a (118 mg, 62%) and 6s (47 mg, 23%). ^1^H NMR (500 MHz, CDCl_3_), *δ*, ppm for 6a: 2.03 (s, 6H; SCH_3_), 2.32 (s, 6H; CCH_3_), 2.40 (s, 6H; SCH_3_), 2.42 (s, 6H; CCH_3_), 6.20 (s, 2H; CH), 7.0–7.24 (m, 20H; Ph); ^13^C NMR (125 MHz, CDCl_3_), *δ*, ppm: 14.09 (2SC̲H_3_), 14.73 (2SC̲H_3_), 25.73 (2CC̲H_3_), 28.69 (2SC̲H_3_), 50.98 (2C̲H), 126.69 (2C4 Ph), 127.05 (4s3 Ph), 127.31 (4s3 Ph), 127.68 (2C4 Ph), 129.98 (4s2 Ph), 130.34 (4s2 Ph), 135.57 (2C1 Ph), 138.61 (2C̲Ph), 140.06 (2C̲Ph), 142.17 (2C1 Ph), 143.14 (2C̲CH_3_), 170.18 (2C̲SCH_3_), 170.65 (2C̲CH_3_), 174.22 (2C̲SCH_3_). ^1^H NMR (500 MHz, CDCl_3_), *δ*, ppm for 6s: 1.95 (s, 6H; CCH_3_), 2.04 (s, 6H; SCH_3_), 2.39 (s, 6H; SCH_3_), 2.85 (s, 6H; CCH_3_), 6.08 (s, 2H; CH), 7.0–7.23 (m, 20H; Ph); ^13^C NMR (125 MHz, CDCl_3_), *δ*, ppm: 14.12 (2SC̲H_3_), 14.79 (2SC̲H_3_), 27.18 (2CC̲H_3_), 27.29 (2CC̲H_3_), 51.03 (2C̲H), 126.73 (2C4 Ph), 127.06 (4s3 Ph), 127.32 (4s3 Ph), 127.70 (2C4 Ph), 129.96 (4s2 Ph), 130.33 (4s2 Ph), 135.48 (2C1 Ph), 138.59 (2C̲Ph), 140.38 (2C̲Ph), 142.05 (2C1 Ph), 144.16 (2C̲CH_3_), 169.87 (2C̲CH_3_), 170.73 (2C̲SCH_3_), 174.47 (2C̲SCH_3_). Positive ion ESI MS for 6a and 6s: *m*/*z* 1069.20 [M + H]^+^.

#### Crystallographic structure determination

SC-XRD measurements for 4a, 4s and 6a were carried out by using a Bruker D8 Venture diffractometer. Single crystals were positioned at 25, 30 and 30 mm from the detector, and 479, 3985 and 3187 frames were measured, each for 60, 20 and 20 s over scan widths of 1, 0.36 and 0.36° for 4a, 4s and 6a, respectively. Data collection for 5s was performed by using an XRD2 beamline, Sincrotrone Elettra Trieste SCpA. A superconducting wiggler acted as a light source for the beamline with a dual crystal Si(111) monochromator providing wavelength selection in the 8–30 keV range. The beamline was equipped with an Arinax MD2S high-throughput diffractometer, a Pilatus 6 M detector and an open flow nitrogen cryostat. Five distinct crystals were measured using a simple 360° omega-scan, 1°/frame, to check sample homogeneity. Data processing and frame integration were performed using the XDS package^77^ as implemented in the XRD4Elettra interface, and space group confirmation was provided by Pointless from the CCP4 suite. The sample presented a low crystallinity in general with maximum resolution up to 1 Å. In the case of the best dataset presented herein, the data were cut at 0.85 Å to obtain reasonable *R*_int_ values. This weak scattering behavior can be attributed to the porosity of the structure and the high mosaicity of the crystal. The structures were solved using intrinsic phasing methods of the SHELXT program and refined by full-matrix least-squares on *F*^2^ with SHELXL^78^ by using the instrumentality of the Olex2^79^ graphical interface. Non-H atoms were refined with anisotropic displacement parameters. The H atoms were inserted at the calculated positions and refined using a riding model. For 4a and 5s, the mask option available in Olex2 was used to model the contribution of the disordered solvent molecules. Crystal data, data collection parameters, and structure refinement details are given in Table S9,† while the geometric parameters are listed in Table S1.†

#### Electrochemistry and spectroelectrochemistry

The cyclic voltammetry (CV) and square-wave voltammetry (SWV) studies were performed by using a homemade miniature electrochemical cell using a platinum-disk as the working electrode (from Ionode, Australia), a platinum wire as the counter electrode, and a silver wire as the pseudoreference electrode. All chemicals were purchased from Sigma-Aldrich at the highest available purity. The 0.1 M tetrabutylammonium hexafluorophosphate (*n*-Bu_4_NPF_6_) in CH_2_Cl_2_ (or CH_2_Cl_2_/CH_3_CN mixtures if solubility issues occurred) was used as the supporting electrolyte. Ferrocene (Fc) served as the internal potential standard and the potentials were determined against a ferricenium/ferrocene couple. A Heka PG310USB (Lambrecht, Germany) potentiostat with the PotMaster 2.73 software package was used for the cyclic voltammetric and spectroelectrochemical studies. *In situ* spectroelectrochemical measurements were performed by using a spectrometer (Avantes, Model AvaSpec-2048x14-USB2) under an argon atmosphere in a spectroelectrochemical cell kit (AKSTCKIT3) with a Pt-microstructured honeycomb working electrode, purchased from Pine Research Instrumentation. Halogen and deuterium lamps were used as the light sources (Avantes, Model AvaLight-DH-S-BAL). The cell was positioned in the CUV-UV cuvette holder (Ocean Optics) connected to the diode-array of a UV–vis–NIR spectrometer by optical fibres. The UV–vis–NIR spectra were recorded using the AvaSoft 7.7 software package.

#### Computational details

The Standard B3LYP/6-311G*^80–86^ geometry optimization at various spin states was performed using the Gaussian09^87^ program package. For open-shell systems, unrestricted DFT formalism was used, *i.e.*, B3LYP implies UB3LYP. In addition, the BLYP and OPBE functionals^88–91^ were taken into consideration to compare the performance of the hybrid and pure GGA (generalized gradient approximation) functionals. Solvent effects on dichloromethane (CH_2_Cl_2_) were approximated by the integral equation formalism polarizable continuum model (IEFPCM),^92,93^ as implemented in Gaussian09. Time-dependent density functional theory (TD-DFT) electronic transitions^94,95^ in dichloromethane were computed for chosen geometries as implemented in Gaussian09. The 80 lowest electron excitations from the ground states were accounted for. The ^13^C and ^1^H NMR chemical shifts were calculated using the Gauge – Including Atomic Orbital (GIAO)^96^ approach as embedded in Gaussian09. TMS (^1^H, ^13^C NMR) was employed as the NMR standard to determine the theoretical chemical shifts. The IQmol package^97^ was used for the visualization of the molecular orbitals and spin densities using the Gaussian09 fchk file. *J*-coupling was evaluated in the following way:1
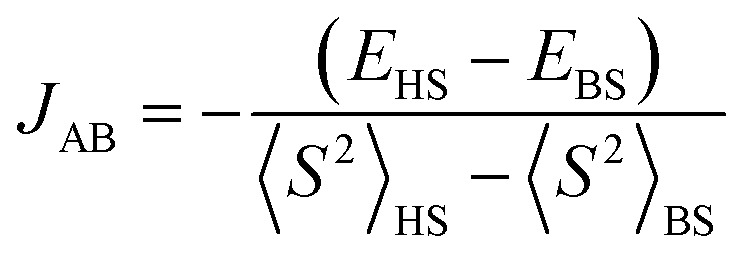
where *E*_HS_ denotes the unrestricted high spin (triplet) total energy, *E*_BS_ denotes the broken symmetry (BS) singlet state energy, and 〈*S*^2^〉_HS_ and 〈*S*^2^〉_BS_ are the particular expectation values of the spin momentum squared.

#### Catalytic studies

The catalytic tests for the peroxidative oxidation of cyclohexane under focused MW irradiation were performed by using an Anton Paar Monowave 300 microwave reactor fitted with a rotational system and an IR temperature detector using sealed cylindrical Pyrex tubes (10 mL capacity reaction vial with a 13 mm internal diameter). The oxidation of cyclohexane (CyH) was performed by the following procedure: CyH (2.5 mmol) and 70% aq. TBHP (5.0 mmol) were added to 10.0 μmol of the catalysts, 4a, 4s, 5s and 6 (as a mixture of 6s and 6a), under homogeneous and solvent-free conditions. The tube was placed in the MW reactor and the mixture was stirred (800 rpm) and irradiated (25 W) for 3 h at 100 °C. An aliquot was taken every 30 min during the reaction, after cooling to room temperature, until its completion. The collected samples were prepared for GC analysis (internal standard method) using nitromethane (50 μL) as a standard compound. An excess of triphenylphosphine was added to reduce the formed cyclohexyl hydroperoxide to the corresponding alcohol, prior to the GC analysis, following a method developed by Shul'pin.^68–71^ Chromatographic analyses were performed by using a Clarus 500 GC series gas chromatograph with a DB-624 (J&W) capillary column (DB-WAX, column length: 30 m; internal diameter: 0.32 mm), flame ionization detector (FID), using the Total Chrom software. The injection temperature was 240 °C. After the injection, the reaction temperature was maintained at 100 °C for 1 min, then increased at 10 °C min^−1^ to 160 °C and held at this temperature for 1 min. Helium was used as the carrier gas. The products were identified by comparison of their retention times with those of the known reference compounds.

## Conflicts of interest

The authors declare no competing financial interest.

## Supplementary Material

DT-051-D2DT00154C-s001

DT-051-D2DT00154C-s002
