# Supplementary material for: Diastereomeric dinickel(ii) complexes with non-innocent bis(octaazamacrocyclic) ligands: isomerization, spectroelectrochemistry, DFT calculations and use in catalytic oxidation of cyclohexane
Source: Dalton Trans. 2022 Feb 28;51(13):5151–67. doi: 10.1039/d2dt00154c (PMC8962992; doi:10.1039/d2dt00154c)
Supplement: DT-051-D2DT00154C-s001 [file DT-051-D2DT00154C-s001.pdf]

Electronic Supplementary Material (ESI) for Dalton Transactions.
This journal is © The Royal Society of Chemistry 2022

**Electronic Supplementary Information**

**for**

# Diastereomeric dinickel(II) complexes with non-innocent bis(octaazamacrocyclic) ligands: isomerization, spectroelectrochemistry, DFT calculations and use in catalytic oxidation of cyclohexane

Anatolie Dobrov,[†,Δ] Denisa Darvasiová,[#] Michal Zalibera,[#,*] Lukáš Bučinský,[#] Ingrid Jelemenská,[#,◊] Peter Rapta,[#] Sergiu Shova,[‡] Dan G. Dumitrescu,[♦] Marta A. Andrade,[§] Luísa M. D. R. S. Martins,[§,*] Armando J. L. Pombeiro,[§,&] Vladimir B. Arion[†,*]

[†]*University of Vienna, Institute of Inorganic Chemistry, Währinger Strasse 42, A-1090 Vienna, Austria*

[Δ]*Universität Wien, Fakultät für Chemie, Institut für Biophysikalische Chemie, 1090 Wien, Austria*

[#]*Institute of Physical Chemistry and Chemical Physics, Faculty of Chemical and Food Technology, Slovak University of Technology in Bratislava, Radlinského 9, SK-81237 Bratislava, Slovak Republic*

[◊]*Department of Chemistry, Faculty of Natural Sciences, Constantine the Philosopher University in Nitra, 949 74 Nitra, Slovak Republic*

[‡]*Inorganic Polymers Department, "Petru Poni" Institute of Macromolecular Chemistry, Aleea Gr. Ghica Voda 41 A, Iasi 700487, Romania*

[♦]*Elettra - Sincrotrone Trieste S.C.p.A., Strada Statale 14 - km 163,5 in AREA Science Park 34149 Basovizza, Trieste, Italy*

[§]*Centro de Química Estrutural, Institute of Molecular Sciences, Universidade de Lisboa, Av. Rovisco Pais, 1049-001 Lisboa, Portugal*

[&]*Peoples' Friendship University of Russia (RUDN University), Research Institute of Chemistry, 6 Miklukho-Maklaya Street, Moscow 117198, Russian Federation*

**Table of Contents**

Crystallography ........ S3

IR spectroscopy ........ S17

NMR spectroscopy ........ S18

Electrochemistry and spectroelectrochemistry ........ S25

UV–vis–NIR spectroscopy ........ S31

DFT calculations ........ S32

# Crystallography

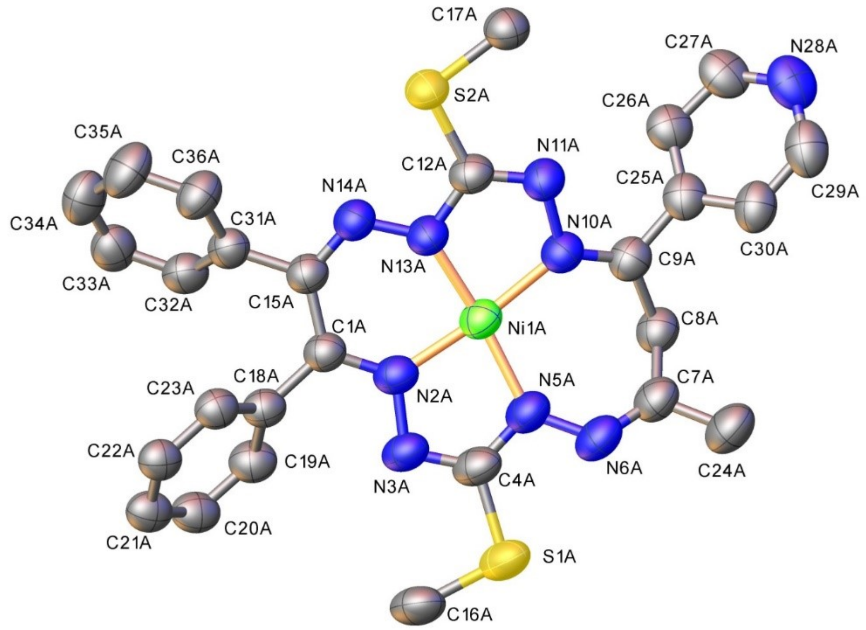


**Figure S1**. View of the asymmetric unit in the crystal structure of **5s** with atom labelling and thermal ellipsoids at 40% probability level. Only a half-moiety of dimeric complex (denoted as **A** part) is shown. Co-crystallized molecules of ethanol are not shown.

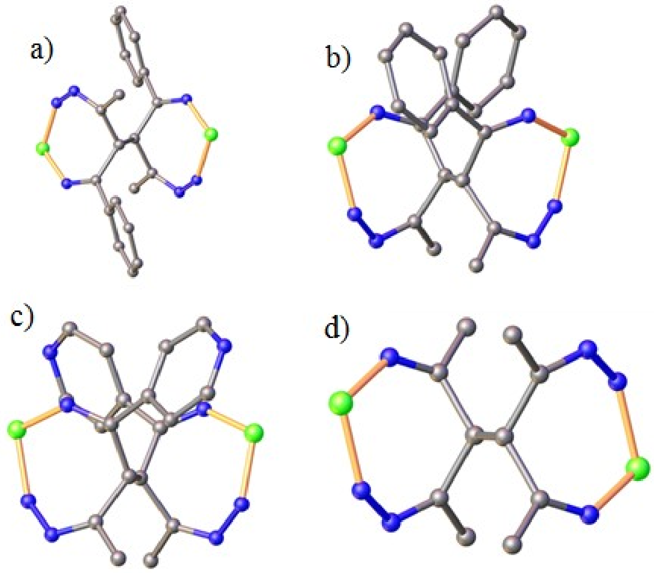


**Figure S2**. Relative arrangements of Ph, Me and Py substituents and N–N backbones at/in 7-membered chelate rings in a) **4a** (*S*,*R*) b) **4s (***S*,*S***)** c) **5s** (*R*,*R*) and d) **6a** (*S*,*R*).

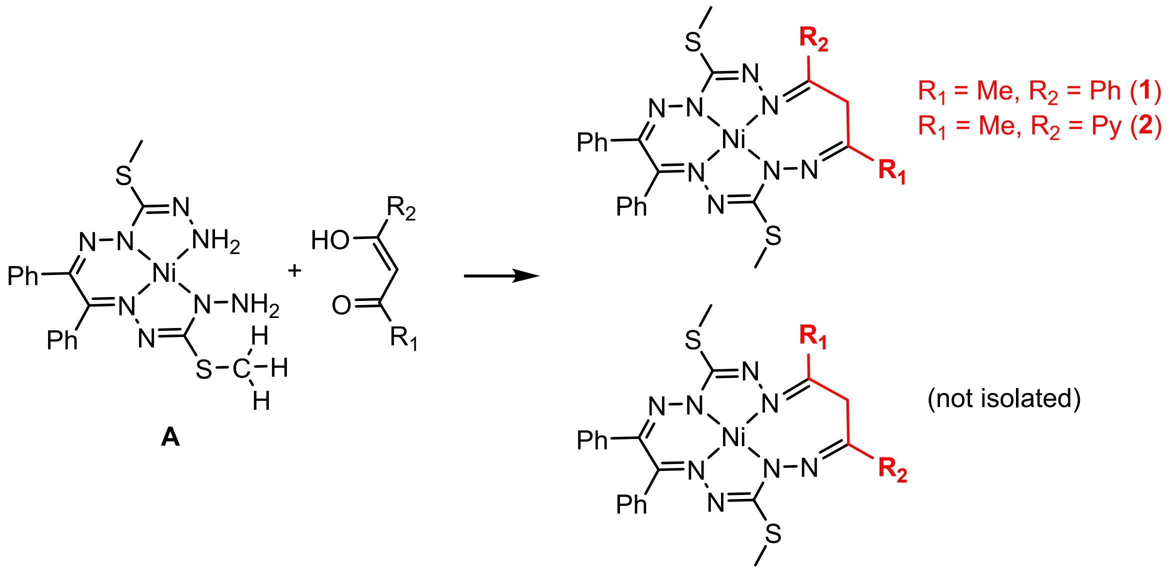


**Scheme S1**. Synthesis of **1**–**2**. Expected products on the bottom have not been isolated.

**Table S1**. Bond distances (Å) and angles (°).

Compound **4a**

| Distances | exp. | DFT | Distances | exp. | DFT |
|---|---|---|---|---|---|
| Ni1-N2 | 1.832(3) | 1.864 | C7-C24 | 1.511(5) | 1.508 |
| Ni1-N5 | 1.864(3) | 1.891 | C8-C8[1] | 1.573(6) | 1.583 |
| Ni1-N10 | 1.889(3) | 1.912 | C8-C9 | 1.518(4) | 1.527 |
| Ni1-N13 | 1.809(3) | 1.832 | C9-C25 | 1.476(4) | 1.490 |
| S1-C4 | 1.747(3) | 1.773 | C15-C31 | 1.499(5) | 1.496 |
| S1-C16 | 1.801(4) | 1.827 | C18-C19 | 1.381(5) | 1.400 |
| S2-C12 | 1.736(3) | 1.764 | C18-C23 | 1.393(5) | 1.398 |
| S2-C17 | 1.803(3) | 1.826 | C19-C20 | 1.384(5) | 1.392 |
| N2-N3 | 1.391(4) | 1.375 | C20-C21 | 1.390(5) | 1.396 |
| N2-C1 | 1.327(4) | 1.319 | C21-C22 | 1.377(5) | 1.393 |
| N3-C4 | 1.314(4) | 1312 | C22-C23 | 1.396(5) | 1.394 |
| N5-N6 | 1.390(4) | 1.363 | C25-C26 | 1.391(5) | 1.401 |
| N5-C4 | 1.358(4) | 1.358 | C25-C30 | 1.402(5) | 1.402 |
| N6-C7 | 1.283(4) | 1.281 | C26-C27 | 1.378(5) | 1.391 |
| N10-N11 | 1.426(4) | 1.402 | C27-C28 | 1.376(6) | 1.394 |
| N10-C9 | 1.310(4) | 1.297 | C28-C29 | 1.375(6) | 1.393 |
| N11-C12 | 1.294(4) | 1.300 | C29-C30 | 1.378(5) | 1.392 |
| N13-N14 | 1.338(4) | 1.323 | C31-C32 | 1.386(5) | 1.401 |
| N13-C12 | 1.381(4) | 1.374 | C31-C36 | 1.394(5) | 1.401 |
| N14-C15 | 1.315(4) | 1.310 | C32-C33 | 1.390(6) | 1.393 |
| C1-C15 | 1.445(5) | 1.459 | C33-C34 | 1.369(6) | 1.394 |
| C1-C18 | 1.488(5) | 1.495 | C34-C35 | 1.373(6) | 1.395 |
| C7-C8 | 1.513(5) | 1.533 | C35-C36 | 1.384(6 | 1.393 |

| Angles | exp. | DFT |
|---|---|---|
| N2-Ni1-N5 | 83.52(12) | 83.4 |
| N2-Ni1-N10 | 173.73(12) | 173.9 |

| | | |
|---|---|---|
| N5-Ni1-N10 | 102.55(12) | 102.1 |
| N13-Ni1-N2 | 91.77(12) | 91.6 |
| N13-Ni1-N5 | 171.46(13) | 169.7 |
| N13-Ni1-N10 | 82.40(12) | 82.5 |
| C4-S1-C16 | 99.59(17) | 100.7 |
| C12-S2-C17 | 99.79(16) | 100.6 |
| N3-N2-Ni1 | 116.3(2) | 115.1 |
| C1-N2-Ni1 | 128.8(2) | 127.2 |
| C1-N2-N3 | 114.8(3) | 117.6 |
| C4-N3-N2 | 108.8(3) | 110.2 |
| N6-N5-Ni1 | 137.2(2) | 135.6 |
| C4-N5-Ni1 | 110.1(2) | 108.7 |
| C4-N5-N6 | 111.2(3) | 112.9 |
| C7-N6-N5 | 119.3(3) | 122.7 |
| N11-N10-Ni1 | 114.4(2) | 112.3 |
| C9-N10-Ni1 | 131.1(2) | 131.3 |
| C9-N10-N11 | 114.5(3) | 116.3 |
| C12-N11-N10 | 108.1(3) | 110.1 |
| N14-N13-Ni1 | 132.6(2) | 131.5 |
| N14-N13-C12 | 114.1(3) | 116.7 |
| C12-N13-Ni1 | 113.3(2) | 111.7 |
| C15-N14-N13 | 119.4(3) | 120.7 |
| N2-C1-C15 | 120.4(3) | 121.4 |
| N2-C1-C18 | 118.8(3) | 119.2 |
| C15-C1-C18 | 120.7(3) | 119.2 |
| N3-C4-S1 | 119.0(3) | 119.4 |
| N3-C4-N5 | 120.7(3) | 121.2 |
| N5-C4-S1 | 120.3(3) | 119.4 |
| N6-C7-C8 | 128.0(3) | 127.6 |
| N6-C7-C24 | 116.1(3) | 116.3 |

| | | |
|---|---|---|
| C24-C7-C8 | 115.8(3) | 116.1 |
| C7-C8-C8$^{1}$ | 112.4(3) | 112.7 |
| C7-C8-C9 | 116.1(3 | 115.0 |

Symmetry code: $^{1}$ 1– *x*, 2–*y*, –*z*.

Compound **4s**

| Distances | exp. | DFT | Distances | exp. | DFT |
|---|---|---|---|---|---|
| C1A-N2A | 1.322(2) | 1.319 | C20B-C21B | 1.387(5) | 1.393 |
| C1A-C15A | 1.455(3) | 1.459 | C21A-C22A | 1.387(4) | 1.396 |
| C1A-C18A | 1.494(3) | 1.496 | C21B-C22B | 1.383(5) | 1.396 |
| C1B-N2B | 1.326(3) | 1.319 | C22A-C23A | 1.395(3) | 1.392 |
| C1B-C15B | 1.458(3) | 1.459 | C22B-C23B | 1.393(3) | 1.392 |
| C1B-C18B | 1.495(3) | 1.496 | C25A-C26A | 1.398(3) | 1.403 |
| C4A-N3A | 1.316(2) | 1.312 | C25A-C30A | 1.401(3) | 1.402 |
| C4A-N5A | 1.351(2) | 1.356 | C25B-C30B | 1.403(3) | 1.403 |
| C4A-S1A | 1.752(2) | 1.773 | C25B-C26B | 1.399(3) | 1.391 |
| `C4B-N3B | 1.316(3) | 1.312 | C26A-C27A | 1.391(3) | 1.392 |
| C4B-N5B | 1.351(2) | 1.356 | C26B-C27B | 1.387(3) | 1.395 |
| C4B-S1B | 1.757(2) | 1.773 | C27A-C28A | 1.391(4) | 1.393 |
| C7A-N6A | 1.288(2) | 1.281 | C27B-C28B | 1.392(4) | 1.393 |
| C7A-C24A | 1.499(3) | 1.506 | C28A-C29A | 1.384(4) | 1.395 |
| C7A-C8A | 1.523(3) | 1.533 | C28B-C29B | 1.391(4) | 1.392 |
| C7B-N6B | 1.284(3) | 1.281 | C29A-C30A | 1.397(3) | 1.391 |
| C7B-C24B | 1.498(3) | 1.506 | C29B-C30B | 1.393(3) | 1.392 |
| C7B-C8B | 1.529(3) | 1.533 | C31A-C32A | 1.391(3) | 1.401 |
| C8A-C9A | 1.518(3) | 1.527 | C31A-C36A | 1.393(3) | 1.401 |
| C8A-C8B | 1.574(3) | 1.584 | C31B-C32B | 1.397(3) | 1.401 |
| C8B-C9B | 1.517(3) | 1.527 | C31B-C36B | 1.402(3) | 1.401 |
| C9A-N10A | 1.298(2) | 1.297 | C32A-C33A | 1.398(3) | 1.393 |
| C9A-C25A | 1.494(3) | 1.492 | C32B-C33B | 1.397(3) | 1.393 |

| | | | | | |
|---|---|---|---|---|---|
| C9B-N10B | 1.297(2) | 1.298 | C33A-C34A | 1.384(3) | 1.394 |
| C9B-C25B | 1.495(3) | 1.491 | C33B-C34B | 1.383(3) | 1.394 |
| C12A-N11A | 1.300(3) | 1.300 | C34A-C35A | 1.386(3) | 1.394 |
| C12A-N13A | 1.376(2) | 1.373 | C34B-C35B | 1.387(3) | 1.394 |
| C12A-S2A | 1.7485(19) | 1.765 | C35A-C36A | 1.393(3) | 1.393 |
| C12B-N11B | 1.303(3) | 1.300 | C35B-C36B | 1.396(3) | 1.393 |
| C12B-N13B | 1.381(2) | 1.373 | N2A-N3A | 1.394(2) | 1.376 |
| C12B-S2B | 1.747(2) | 1.765 | N2A-Ni1A | 1.850(2) | 1.867 |
| C15A-N14A | 1.314(3) | 1.310 | N2B-N3B | 1.393(2) | 1.376 |
| C15A-C31A | 1.496(3) | 1.496 | N2B-Ni1B | 1.853(2) | 1.866 |
| C15B-N14B | 1.315(3) | 1.310 | N5A-N6A | 1.390(2) | 1.369 |
| C15B-C31B | 1.498(3) | 1.496 | N5A-Ni1A | 1.868(2) | 1.889 |
| C16A-S1A | 1.803(2) | 1.827 | N5B-N6B | 1.390(2) | 1.369 |
| C16B-S1B | 1.810(2) | 1.827 | N5B-Ni1B | 1.868(2) | 1.888 |
| C17A-S2A | 1.806(2) | 1.827 | N10A-N11A | 1.417(2) | 1.402 |
| C17B-S2B | 1.805(2) | 1.826 | N10A-Ni1A | 1.906(2) | 1.919 |
| C18A-C23A | 1.396(3) | 1.399 | N10B-N11B | 1.420(2) | 1.401 |
| C18A-C19A | 1.400(3) | 1.398 | N10B-Ni1B | 1.903(2) | 1.920 |
| C18B-C19B | 1.396(3) | 1.398 | N13A-N14A | 1.339(2) | 1.323 |
| C18B-C23B | 1.398(3) | 1.399 | N13A-Ni1A | 1.806(2) | 1.831 |
| C19A-C20A | 1.388(3) | 1.394 | N13B-N14B | 1.330(2) | 1.323 |
| C19B-C20B | 1.390(3) | 1.394 | N13B-Ni1B | 1.806(2) | 1.831 |
| C20A-C21A | 1.386(4) | 1.393 | | | |

| Angles | exp. | DFT | Angles | exp. | DFT |
|---|---|---|---|---|---|
| N2A-C1A-C15A | 120.71(17) | 121.5 | C29B-C28B-C27B | 119.8(2) | 119.6 |
| N2A-C1A-C18A | 119.31(17) | 119.2 | C28A-C29A-C30A | 120.3(2) | 120.1 |
| C15A-C1A-C18A | 119.98(16) | 119.2 | C28B-C29B-C30B | 120.1(2) | 120.4 |
| N2B-C1B-C15B | 120.78(17) | 121.5 | C29A-C30A-C25A | 120.4(2) | 120.8 |
| N2B-C1B-C18B | 118.31(18) | 119.2 | C29B-C30B-C25B | 120.3(2) | 120.6 |

| | | | | | |
|---|---|---|---|---|---|
| C15B-C1B-C18B | 119.98(16) | 119.2 | C32A-C31A-C36A | 119.39(18) | 118.5 |
| N3A-C4A-N5A | 121.20(17) | 121.3 | C32A-C31A-C15A | 118.92(18) | 121.6 |
| N3A-C4A-S1A | 121.20(17) | 119.6 | C36A-C31A-C15A | 118.92(18) | 119.8 |
| N5A-C4A-S1A | 119.96(14) | 119.1 | C32B-C31B-C36B | 118.71(19) | 118.5 |
| N3B-C4B-N5B | 118.81(14) | 121.3 | C32B-C31B-C15B | 123.12(19) | 121.6 |
| N3B-C4B-S1B | 121.21(17) | 119.6 | C36B-C31B-C15B | 118.03(18) | 119.8 |
| N5B-C4B-S1B | 119.66(15) | 119.1 | C31A-C32A-C33A | 119.8(2) | 120.8 |
| N6A-C7A-C24A | 117.00(17) | 116.3 | C33B-C32B-C31B | 120.3(2) | 120.8 |
| N6A-C7A-C8A | 127.38(17) | 127.1 | C34A-C33A-C32A | 120.4(2) | 120.2 |
| C24A-C7A-C8A | 115.57(16) | 116.6 | C34B-C33B-C32B | 120.6(2) | 120.2 |
| N6B-C7B-C24B | 116.70(17) | 116.3 | C33A-C34A-C35A | 120.1(2) | 119.5 |
| N6B-C7B-C8B | 127.12(17) | 127.2 | C35B-C34B-C33B | 119.8(2) | 119.5 |
| C24B-C7B-C8B | 116.09(17) | 116.5 | C34A-C35A-C36A | 119.6(2) | 120.3 |
| C9A-C8A-C7A | 115.20(15) | 114.8 | C34B-C35B-C36B | 120.1(2) | 120.3 |
| C9A-C8A-C8B | 114.44(15) | 114.0 | C35A-C36A-C31A | 120.7(2) | 120.7 |
| C7A-C8A-C8B | 110.23(15) | 110.7 | C35B-C36B-C31B | 120.5(2) | 120.7 |
| C9B-C8B-C7B | 116.24(16) | 114.8 | C1A-N2A-N3A | 116.17(16) | 117.4 |
| C9B-C8B-C8A | 113.48(15) | 114.0 | C1A-N2A-Ni1A | 127.69(13) | 127.2 |
| C7B-C8B-C8A | 109.66(15) | 110.7 | N3A-N2A-Ni1A | 116.13(12) | 115.2 |
| N10A-C9A-C25A | 124.55(17) | 123.3 | C1B-N2B-N3B | 116.25(16) | 117.5 |
| N10A-C9A-C8A | 120.03(16) | 120.3 | C1B-N2B-Ni1B | 128.31(14) | 127.2 |
| C25A-C9A-C8A | 115.35(16) | 116.3 | N3B-N2B-Ni1B | 115.39(13) | 115.3 |
| N10B-C9B-C25B | 123.62(17) | 123.3 | C4A-N3A-N2A | 108.62(15) | 110.1 |
| N10B-C9B-C8B | 120.66(17) | 120.3 | C4B-N3B-N2B | 109.29(16) | 110.1 |
| C25B-C9B-C8B | 115.65(16) | 116.4 | C4A-N5A-N6A | 113.74(15) | 113.4 |
| N11A-C12A-N13A | 120.29(17) | 120.1 | C4A-N5A-Ni1A | 110.51(12) | 109.2 |
| N11A-C12A-S2A | 122.69(15) | 121.4 | N6A-N5A-Ni1A | 133.37(13) | 133.9 |
| N13A-C12A-S2A | 117.01(14) | 118.6 | C4B-N5B-N6B | 113.21(16) | 113.5 |
| N11B-C12B-N13B | 119.89(18) | 120.0 | C4B-N5B-Ni1B | 110.30(13) | 109.2 |
| N11B-C12B-S2B | 122.78(15) | 121.3 | N6B-N5B-Ni1B | 134.17(13) | 133.6 |

| | | | | | |
|---|---|---|---|---|---|
| N13B-C12B-S2B | 117.32(15) | 118.6 | C7A-N6A-N5A | 118.40(16) | 121.7 |
| N14A-C15A-C1A | 126.28(17) | 125.7 | C7B-N6B-N5B | 118.97(16) | 121.6 |
| N14A-C15A-C31A | 112.52(16) | 113.7 | C9A-N10A-N11A | 115.49(16) | 116.2 |
| C1A-C15A-C31A | 120.99(17) | 120.5 | C9A-N10A-Ni1A | 130.31(13) | 131.3 |
| N14B-C15B-C1B | 125.58(18) | 125.7 | N11A-N10A-Ni1A | 113.87(12) | 112.5 |
| N14B-C15B-C31B | 111.15(17) | 113.6 | C9B-N10B-N11B | 114.96(16) | 116.2 |
| C1B-C15B-C31B | 111.15(17) | 120.6 | C9B-N10B-Ni1B | 130.55(14) | 131.3 |
| C23A-C18A-C19A | 119.12(19) | 119.2 | N11B-N10B-Ni1B | 113.94(12) | 112.4 |
| C23A-C18A-C1A | 122.30(18) | 120.6 | C12A-N11A-N10A | 108.48(15) | 110.2 |
| C19A-C18A-C1A | 118.57(18) | 120.2 | C12B-N11B-N10B | 108.44(16) | 110.2 |
| C19B-C18B-C23B | 119.3(2) | 119.2 | N14A-N13A-C12A | 114.51(16) | 116.5 |
| C19B-C18B-C1B | 119.9(2) | 120.6 | N14A-N13A-Ni1A | 132.31(13) | 131.6 |
| C23B-C18B-C1B | 120.8(2) | 120.6 | C12A-N13A-Ni1A | 113.06(13) | 111.8 |
| C20A-C19A-C18A | 120.5(2) | 120.4 | N14B-N13B-C12B | 114.17(16) | 116.5 |
| C20B-C19B-C18B | 120.1(3) | 120.4 | N14B-N13B-Ni1B | 132.58(14) | 131.6 |
| C19A-C20A-C21A | 120.0(2) | 120.2 | C12B-N13B-Ni1B | 113.23(13) | 111.8 |
| C21B-C20B-C19B | 120.3(3) | 120.2 | C15A-N14A-N13A | 119.75(16) | 120.8 |
| C22A-C21A-C20A | 120.1(2) | 119.7 | C15B-N14B-N13B | 120.60(17) | 120.8 |
| C22B-C21B-C20B | 119.9(2) | 119.7 | N13A-Ni1A-N2A | 92.27(7) | 91.9 |
| C21A-C22A-C23A | 120.2(2) | 120.2 | N13A-Ni1A-N5A | 169.01(8) | 169.8 |
| C21B-C22B-C23B | 120.4(3) | 120.2 | N2A-Ni1A-N5A | 83.24(7) | 83.3 |
| C22A-C23A-C18A | 120.0(2) | 120.4 | N13A-Ni1A-N10A | 82.50(7) | 82.6 |
| C22B-C23B-C18B | 120.0(3) | 120.4 | N2A-Ni1A-N10A | 172.32(7) | 174.5 |
| C26A-C25A-C30A | 118.72(18) | 118.5 | N5A-Ni1A-N10A | 102.88(7) | 102.2 |
| C26A-C25A-C9A | 121.83(18) | 122.3 | N13B-Ni1B-N2B | 91.84(7) | 91.9 |
| C30A-C25A-C9A | 119.22(17) | 119.2 | N13B-Ni1B-N5B | 170.96(8) | 169.8 |
| C30B-C25B-C26B | 119.15(19) | 118.5 | N2B-Ni1B-N5B | 83.71(7) | 83.3 |
| C30B-C25B-C9B | 119.61(18) | 119.2 | N13B-Ni1B-N10B | 82.50(7) | 82.6 |
| C26B-C25B-C9B | 121.07(18) | 122.3 | N2B-Ni1B-N10B | 173.11(7) | 174.4 |
| C27A-C26A-C25A | 120.5(2) | 120.6 | N5B-Ni1B-N10B | 102.44(7) | 102.3 |

| | | | | | |
|---|---|---|---|---|---|
| C27B-C26B-C25B | 120.2(2) | 120.8 | C4A-S1A-C16A | 101.30(10) | 100.9 |
| C26A-C27A-C28A | 120.3(2) | 120.4 | C4B-S1B-C16B | 101.43(10) | 100.8 |
| C26B-C27B-C28B | 120.3(2) | 120.1 | C12A-S2A-C17A | 100.65(10) | 100.7 |
| C29A-C28A-C27A | 119.7(2) | 119.6 | C12B-S2B-C17B | 100.16(10) | 100.6 |

Compound **5s**

| Distances | exp. | DFT | Distances | exp. | DFT |
|---|---|---|---|---|---|
| C1A-C15A | 1.449(5) | 1.462 | N2A-Ni1A | 1.844(3) | 1.864 |
| C1A-C18A | 1.484(5) | 1.495 | N5A-N6A | 1.390(5) | 1.370 |
| C1A-N2A | 1.333(5) | 1.318 | N5A-Ni1A | 1.875(3) | 1.893 |
| C4A-N3A | 1.305(5) | 1.313 | N10A-N11A | 1.419(4) | 1.399 |
| C4A-N5A | 1.355(5) | 1.357 | N10A-Ni1A | 1.896(3) | 1.917 |
| C4A-S1A | 1.752(4) | 1.770 | N13A-N14A | 1.350(4) | 1.326 |
| C7A-C8A | 1.512(6) | 1.535 | N13A-Ni1A | 1.807(3) | 1.831 |
| C7A-C24A | 1.499(6) | 1.504 | C1B-C15B | 1.448(6) | 1.462 |
| C7A-N6A | 1.293(6) | 1.278 | C1B-N2B | 1.330(5) | 1.318 |
| C8A-C9A | 1.523(5) | 1.524 | C4B-N3B | 1.339(6) | 1.313 |
| C8A-C8B | 1.570(5) | 1.578 | C4B-N5B | 1.286(5) | 1.357 |
| C9A-C25A | 1.488(5) | 1.488 | C4B-S1B | 1.747(5) | 1.770 |
| C9A-N10A | 1.293(5) | 1.298 | C7B-C8B | 1.531(5) | 1.535 |
| C12A-N11A | 1.311(5) | 1.304 | C7B-C24B | 1.516(6) | 1.504 |
| C12A-N13A | 1.373(4) | 1.373 | C7B-N6B | 1.274(5) | 1.280 |
| C12A-S2A | 1.738(3) | 1.761 | C8B-C9B | 1.505(6) | 1.524 |
| C15A-C31A | 1.492(5) | 1.496 | C9B-C25B | 1.495(6) | 1.489 |
| C15A-N14A | 1.311(4) | 1.309 | C9B-N10B | 1.283(5) | 1.298 |
| C16A-S1A | 1.784(5) | 1.827 | C12B-N11B | 1.312(5) | 1.304 |
| C17A-S2A | 1.796(4) | 1.827 | C12B-N13B | 1.389(5) | 1.373 |
| C18A-C19A | 1.405(5) | 1.398 | C12B-S2B | 1.728(4) | 1.761 |
| C18A-C23A | 1.387(5) | 1.399 | C15B-N14B | 1.290(5) | 1.309 |
| C19A-C20A | 1.383(6) | 1.394 | C16B-S1B | 1.799(6) | 1.827 |

| | | | | | |
|---|---|---|---|---|---|
| C20A-C21A | 1.373(6) | 1.393 | C17B-S2B | 1.789(5) | 1.827 |
| C21A-C22A | 1.377(5) | 1.396 | C25B-C26B | 1.388(6) | 1.397 |
| C22A-C23A | 1.381(5) | 1.391 | C25B-C30B | 1.372(6) | 1.401 |
| C25A-C26A | 1.393(6) | 1.397 | C26B-C27B | 1.380(6) | 1.393 |
| C25A-C30A | 1.388(6) | 1.401 | C27B-N28B | 1.307(6) | 1.336 |
| C26A-C27A | 1.374(6) | 1.393 | C29B-C30B | 1.389(7) | 1.389 |
| C27A-N28A | 1.331(6) | 1.336 | C29B-N28B | 1.346(6) | 1.340 |
| C29A-C30A | 1.400(6) | 1.389 | N2B-N3B | 1.393(5) | 1.375 |
| C29A-N28A | 1.331(6) | 1.340 | N2B-Ni1B | 1.839(3) | 1.864 |
| C31A-C32A | 1.373(5) | 1.401 | N5B-N6B | 1.396(5) | 1.370 |
| C31A-C36A | 1.387(5) | 1.401 | N5B-Ni1B | 1.882(4) | 1.862 |
| C32A-C33A | 1.396(6) | 1.393 | N10B-N11B | 1.423(4) | 1.400 |
| C33A-C34A | 1.355(7) | 1.394 | N10B-Ni1B | 1.892(3) | 1.917 |
| C34A-C35A | 1.360(7) | 1.394 | N13B-N14B | 1.333(5) | 1.326 |
| C35A-C36A | 1.378(6) | 1.393 | N13B-Ni1B | 1.793(4) | 1.831 |
| N2A-N3A | 1.392(4) | 1.375 | | | |

| Angles | exp. | DFT | Angles | exp. | DFT |
|---|---|---|---|---|---|
| C15A-C1A-C18A | 119.4(3) | 119.1 | C27A-N28A-C29A | 115.9(4) | 116.9 |
| N2A-C1A-C15A | 120.8(3) | 121.4 | N2A-Ni1A-N5A | 83.42(14) | 83.4 |
| N2A-C1A-C18A | 119.7(3) | 119.3 | N2A-Ni1A-N10A | 173.19(13) | 174.5 |
| N3A-C4A-N5A | 121.6(3) | 121.2 | N5A-Ni1A-N10A | 102.37(14) | 102.0 |
| N3A-C4A-S1A | 118.6(3) | 119.5 | N13A-Ni1A-N2A | 91.98(13) | 92.1 |
| N5A-C4A-S1A | 119.7(3) | 119.2 | N13A-Ni1A-N5A | 171.17(14) | 170.5 |
| C24A-C7A-C8A | 116.0(4) | 116.1 | N13A-Ni1A-N10A | 82.74(13) | 82.4 |
| N6A-C7A-C8A | 126.8(4) | 126.7 | C4A-S1A-C16A | 100.1(2) | 100.8 |
| N6A-C7A-C24A | 116.9(4) | 117.3 | C12A-S2A-C17A | 100.82(18) | 100.7 |
| C7A-C8A-C8B | 115.6(3) | 115.4 | C15B-C1B-C18B | 125.1(7) | 119.1 |
| C9A-C8A-C7A | 119.4(3) | 109.4 | N2B-C1B-C15B | 120.5(4) | 121.4 |
| C9A-C8A-C8B | 114.1(3) | 113.5 | N2B-C1B-C18B | 114.4(7) | 119.3 |

| | | | | | |
|---|---|---|---|---|---|
| C25A-C9A-C8A | 115.6(3) | 117.9 | N3B-C4B-S1B | 118.2(3) | 119.6 |
| N10A-C9A-C8A | 121.2(3) | 119.7 | N5B-C4B-N3B | 122.0(4) | 121.2 |
| N10A-C9A-C25A | 123.1(3) | 122.3 | N5B-C4B-S1B | 119.7(4) | 119.2 |
| N11A-C12A-N13A | 119.9(3) | 119.9 | C24B-C7B-C8B | 115.0(4) | 116.1 |
| N11A-C12A-S2A | 121.8(3) | 121.2 | N6B-C7B-C8B | 126.9(4) | 126.7 |
| N13A-C12A-S2A | 118.4(3) | 118.9 | N6B-C7B-C24B | 118.2(4) | 117.2 |
| C1A-C15A-C31A | 120.0(3) | 120.5 | C7B-C8B-C8A | 109.3(3) | 115.4 |
| N14A-C15A-C1A | 126.0(3) | 125.8 | C9B-C8B-C8A | 114.3(3) | 113.5 |
| N14A-C15A-C31A | 114.0(3) | 113.7 | C9B-C8B-C7B | 115.2(3) | 109.4 |
| C19A-C18A-C1A | 122.7(3) | 120.2 | C25B-C9B-C8B | 115.4(4) | 118.0 |
| C23A-C18A-C1A | 119.0(3) | 120.6 | N10B-C9B-C8B | 121.4(4) | 119.8 |
| C23A-C18A-C19A | 118.2(3) | 119.2 | N10B-C9B-C25B | 123.1(4) | 122.2 |
| C20A-C19A-C18A | 120.0(4) | 120.4 | N11B-C12B-N13B | 118.8(4) | 119.9 |
| C21A-C20A-C19A | 120.8(3) | 120.2 | N11B-C12B-S2B | 121.5(3) | 121.1 |
| C22A-C21A-C20A | 119.7(4) | 119.7 | N13B-C12B-S2B | 119.7(3) | 118.9 |
| C21A-C22A-C23A | 120.3(4) | 120.2 | C26B-C25B-C9B | 122.4(3) | 122.2 |
| C22A-C23A-C18A | 121.0(3) | 120.4 | C26B-C25B-C30B | 117.8(4) | 117.4 |
| C26A-C25A-C9A | 122.5(4) | 120.0 | C30B-C25B-C9B | 119.5(4) | 120.3 |
| C30A-C25A-C9A | 120.1(4) | 120.5 | C27B-C26B-C25B | 119.1(4) | 119.0 |
| C30A-C25A-C26A | 117.2(4) | 117.6 | N28B-C27B-C26B | 123.9(4) | 123.8 |
| C27A-C26A-C25A | 119.5(4) | 119.1 | N28B-C29B-C30B | 123.0(4) | 123.8 |
| N28A-C27A-C26A | 124.5(5) | 123.8 | C29B-C30B-C25B | 119.0(4) | 119.1 |
| N28A-C29A-C30A | 124.4(4) | 123.9 | C1B-N2B-N3B | 115.8(3) | 117.7 |
| C25A-C30A-C29A | 114.1(3) | 119.0 | C1B-N2B-Ni1B | 128.5(3) | 127.0 |
| C32A-C31A-C15A | 115.6(3) | 121.4 | N3B-N2B-Ni1B | 115.6(3) | 115.3 |
| C32A-C31A-C36A | 121.2(3) | 118.6 | C4B-N3B-N2B | 108.0(3) | 110.2 |
| C36A-C31A-C15A | 123.1(3) | 119.9 | C4B-N5B-N6B | 114.8(4) | 113.3 |
| C31A-C32A-C33A | 119.9(3) | 120.7 | C4B-N5B-Ni1B | 110.9(3) | 109.0 |
| C34A-C33A-C32A | 121.8(3) | 120.3 | N6B-N5B-Ni1B | 131.8(3) | 133.8 |
| C33A-C34A-C35A | 119.8(4) | 119.6 | C7B-N6B-N5B | 121.6(3) | 121.6 |

| | | | | | |
|---|---|---|---|---|---|
| C34A-C35A-C36A | 120.5(5) | 120.2 | C9B-N10B-N11B | 113.8(3) | 116.5 |
| C35A-C36A-C31A | 120.6(4) | 120.7 | C9B-N10B-Ni1B | 131.7(3) | 130.3 |
| C1A-N2A-N3A | 115.4(3) | 117.8 | N11B-N10B-Ni1B | 114.2(2) | 113.0 |
| C1A-N2A-Ni1A | 128.2(2) | 127.0 | C12B-N11B-N10B | 108.5(3) | 109.9 |
| N3A-N2A-Ni1A | 116.0(2) | 115.3 | C12B-N13B-Ni1B | 114.0(3) | 112.2 |
| C4A-N3A-N2A | 108.9(3) | 110.2 | N14B-N13B-C12B | 113.3(4) | 116.6 |
| C4A-N5A-N6A | 113.0(3) | 113.4 | N14B-N13B-Ni1B | 132.7(3) | 131.3 |
| C4A-N5A-Ni1A | 109.7(2) | 109.0 | C15B-N14B-N13B | 120.8(4) | 120.8 |
| N6A-N5A-Ni1A | 134.3(3) | 133.8 | C27B-N28B-C29B | 117.2(4) | 116.9 |
| C7A-N6A-N5A | 119.1(3) | 121.6 | N2B-Ni1B-N5B | 83.05(16) | 83.4 |
| C9A-N10A-N11A | 114.1(3) | 116.6 | N2B-Ni1B-N10B | 173.28(14) | 174.5 |
| C9A-N10A-Ni1A | 130.9(3) | 130.2 | N5B-Ni1B-N10B | 103.16(15) | 101.9 |
| N11A-N10A-Ni1A | 114.5(2) | 112.8 | N13B-Ni1B-N2B | 91.32(16) | 92.1 |
| C12A-N11A-N10A | 108.2(3) | 109.9 | N13B-Ni1B-N5B | 171.65(14) | 170.3 |
| C12A-N13A-Ni1A | 113.6(2) | 112.1 | N13B-Ni1B-N10B | 82.78(15) | 82.4 |
| N14A-N13A-C12A | 113.7(3) | 116.6 | C4B-S1B-C16B | 101.0(3) | 101.0 |
| N14A-N13A-Ni1A | 132.2(2) | 131.3 | C12B-S2B-C17B | 100.9(2) | 100.6 |
| C15A-N14A-N13A | 119.8(3) | 120.8 | | | |

Compound **6a**

| Distances | exp. | DFT | Distances | exp. | DFT |
|---|---|---|---|---|---|
| Ni1-N13 | 1.818(4) | 1.831 | C15-C1 | 1.461(8) | 1.459 |
| Ni1-N2 | 1.848(5) | 1.864 | C15-C31 | 1.488(8) | 1.496 |
| Ni1-N10 | 1.882(5) | 1.912 | C8-C7 | 1.521(8) | 1.530 |
| Ni1-N5 | 1.884(4) | 1.894 | C8-C8[1] | 1.570(11) | 1.574 |
| S2-C12 | 1.733(6) | 1.765 | N11-C12 | 1.302(8) | 1.299 |
| S2-C17 | 1.797(7) | 1.826 | C1-C18 | 1.503(9) | 1.496 |
| S1-C4 | 1.759(5) | 1.774 | N6-C7 | 1.288(8) | 1.281 |
| S1-C16 | 1.793(7) | 1.827 | C18-C19 | 1.384(9) | 1.398 |
| N5-C4 | 1.297(8) | 1.356 | C18-C23 | 1.392(9) | 1.399 |

| | | | | | |
|---|---|---|---|---|---|
| N5-N6 | 1.400(7) | 1.372 | C22-C21 | 1.320(10) | 1.396 |
| N10-C9 | 1.299(8) | 1.293 | C22-C23 | 1.410(9) | 1.392 |
| N10-N11 | 1.431(6) | 1.406 | C34-C35 | 1.380(10) | 1.394 |
| N3-C4 | 1.326(8) | 1.312 | C34-C33 | 1.383(10) | 1.394 |
| N3-N2 | 1.378(6) | 1.376 | C31-C32 | 1.395(9) | 1.401 |
| N13-N14 | 1.328(7) | 1.323 | C31-C36 | 1.399(9) | 1.401 |
| N13-C12 | 1.385(8) | 1.377 | C24-C7 | 1.505(8) | 1.505 |
| N14-C15 | 1.317(8) | 1.310 | C33-C32 | 1.390(9) | 1.393 |
| N2-C1 | 1.321(8) | 1.319 | C19-C20 | 1.381(9) | 1.394 |
| C9-C25 | 1.493(8) | 1.498 | C36-C35 | 1.406(9) | 1.393 |
| C9-C8 | 1.512(8) | 1.519 | C21-C20 | 1.422(11) | 1.393 |

| Angles | exp. | DFT | Angles | exp. | DFT |
|---|---|---|---|---|---|
| N13-Ni1-N2 | 91.3(2) | 92.1 | C9-C8-C7 | 117.2(5) | 113.2 |
| N13-Ni1-N10 | 83.2(2) | 82.6 | C9-C8-C8$^{1}$ | 110.2(5) | 112.6 |
| N2-Ni1-N10 | 174.1(2) | 174.7 | C7-C8-C8$^{1}$ | 111.3(6) | 113.1 |
| N13-Ni1-N5 | 172.6(2) | 170.1 | C12-N11-N10 | 108.5(5) | 109.9 |
| N2-Ni1-N5 | 83.0(2) | 83.3 | N11-C12-N13 | 120.0(5) | 120.0 |
| N10-Ni1-N5 | 102.7(2) | 102.0 | N11-C12-S2 | 122.2(5) | 121.4 |
| C12-S2-C17 | 98.9(3) | 100.4 | N13-C12-S2 | 117.8(4) | 118.6 |
| C4-S1-C16 | 101.1(3) | 100.9 | N2-C1-C15 | 121.9(6) | 121.4 |
| C4-N5-N6 | 113.2(4) | 113.3 | N2-C1-C18 | 118.0(5) | 119.2 |
| C4-N5-Ni1 | 110.4(4) | 109.1 | C15-C1-C18 | 120.1(5) | 119.2 |
| N6-N5-Ni1 | 135.3(4) | 134.4 | C7-N6-N5 | 122.2(5) | 121.4 |
| C9-N10-N11 | 112.7(5) | 115.7 | C19-C18-C23 | 119.2(6) | 119.2 |
| C9-N10-Ni1 | 133.1(4) | 131.2 | C19-C18-C1 | 122.2(6) | 120.2 |
| N11-N10-Ni1 | 114.2(4) | 113.1 | C23-C18-C1 | 118.6(6) | 120.7 |
| C4-N3-N2 | 108.8(5) | 110.2 | C21-C22-C23 | 121.1(7) | 120.2 |
| N14-N13-C12 | 114.6(4) | 116.4 | C35-C34-C33 | 120.8(6) | 119.5 |
| N14-N13-Ni1 | 132.7(4) | 131.4 | C18-C23-C22 | 119.8(6) | 120.4 |

| | | | | | |
|---|---|---|---|---|---|
| C12-N13-Ni1 | 112.7(4) | 112.2 | C32-C31-C36 | 117.8(6) | 118.5 |
| C15-N14-N13 | 121.1(5) | 121.0 | C32-C31-C15 | 124.3(6) | 121.6 |
| C1-N2-N3 | 116.2(5) | 117.4 | C36-C31-C15 | 117.7(6) | 119.8 |
| C1-N2-Ni1 | 128.2(4) | 127.2 | N6-C7-C24 | 117.1(5) | 117.2 |
| N3-N2-Ni1 | 115.4(4) | 115.3 | N6-C7-C8 | 127.0(5) | 127.0 |
| N10-C9-C25 | 122.4(5) | 122.9 | C24-C7-C8 | 115.7(5) | 115.8 |
| N10-C9-C8 | 120.4(5) | 120.4 | C34-C33-C32 | 119.7(6) | 120.3 |
| C25-C9-C8 | 116.8(5) | 116.7 | C33-C32-C31 | 121.4(6) | 120.7 |
| N5-C4-N3 | 122.2(5) | 121.4 | C20-C19-C18 | 120.1(6) | 120.4 |
| N5-C4-S1 | 119.5(5) | 119.1 | C31-C36-C35 | 121.2(6) | 120.8 |
| N3-C4-S1 | 118.3(5) | 119.5 | C34-C35-C36 | 119.1(6) | 120.2 |
| N14-C15-C1 | 124.1(6) | 125.8 | C22-C21-C20 | 119.7(7) | 120.2 |
| N14-C15-C31 | 112.3(5) | 113.6 | C19-C20-C21 | 120.1(7) | 120.2 |
| C1-C15-C31 | 123.6(6) | 120.6 | | | |

Symmetry code: [1] 1– *x*, –*y*, 2–*z*.

**Table S2**. Deviations (Å) from the mean least-squares plane in the seven-membered chelate ring(s).

| Atom | **4a** | **4s** | | **5s** | | **6a** |
|---|---|---|---|---|---|---|
| | | Part A | Part B | Part A | Part B | |
| Ni1* | 0.075 | –0.031 | –0.028 | –0.023 | 0.019 | 0.044 |
| N5* | 0.094 | –0.182 | –0.181 | –0.177 | 0.172 | 0.108 |
| N6* | –0.200 | 0.203 | 0.191 | 0.199 | –0.191 | –0.176 |
| C7* | 0.113 | –0.041 | –0.030 | –0.044 | 0.039 | 0.082 |
| C9* | 0.083 | –0.178 | –0.184 | –0.158 | 0.157 | 0.083 |
| N10* | –0.165 | 0.228 | 0.231 | 0.204 | –0.197 | –0.141 |
| C8 | 0.854(5) | 0.877(2) | –0.856(2) | –0.846(6) | –0.856(6) | 0.807(7) |
| RMS deviation of fitted atoms | 0.130 | 0.164 | 0.162 | 0.153 | 0.148 | 0.114 |

*indicates atom used to define plane

## IR spectroscopy

Most characteristic bands ($cm^{-1}$) for **4a**: 3053w, 2920m, 1597w, 1576w, 1529w, 1489vs, 1462s, 1441s, 1399vs, 1338m, 1319m, 1300vs, 1212vs, 1137s, 1074s, 941vs, 758s, 722m, 707m, 692vs, 592s, 565s and **4s**: 3056m, 3024m, 2924m, 1588m, 1573w, 1527m, 1486vs, 1456s, 1441s, 1399s, 1336s, 1325s, 1298vs, 1247s, 1213vs, 1187m, 1174s, 1140s, 940vs, 916s, 759s, 732s, 693vs, 672s, 592s, 568s.

Most characteristic bands ($cm^{-1}$) for **5s**: 3052w, 3021w 2997w, 2924m, 1585m, 1541m, 1482vs, 1442s, 1405s, 1321s, 1296vs, 1250m, 1209vs, 1137s, 1071s, 1026s, 1003s, 938vs, 918vs, 702vs, 675s, 622s, 591s, 564s, 517m, 480m.

Most characteristic bands ($cm^{-1}$) for **6s**: 3058w, 3020w 2995w, 2926m, 1606m, 1530s, 1499s, 1491s, 1461s, 1444m, 1395s, 1301vs, 1257s, 1248s, 1207vs, 1131s, 1077m, 949vs, 910s, 754s, 730s, 694vs, 665s, 621s, 605s, 572s, 463m, 414m. for **6a**: 3057w, 3022w 2985w, 2923m, 1602m, 1529m, 1490vs, 1458s, 1444m, 1394s, 1298vs, 1248s, 1202vs, 1131s, 1078m, 949vs, 907s, 754s, 732s, 699vs, 666s, 621s, 606s, 566s, 416m.

## NMR spectroscopy

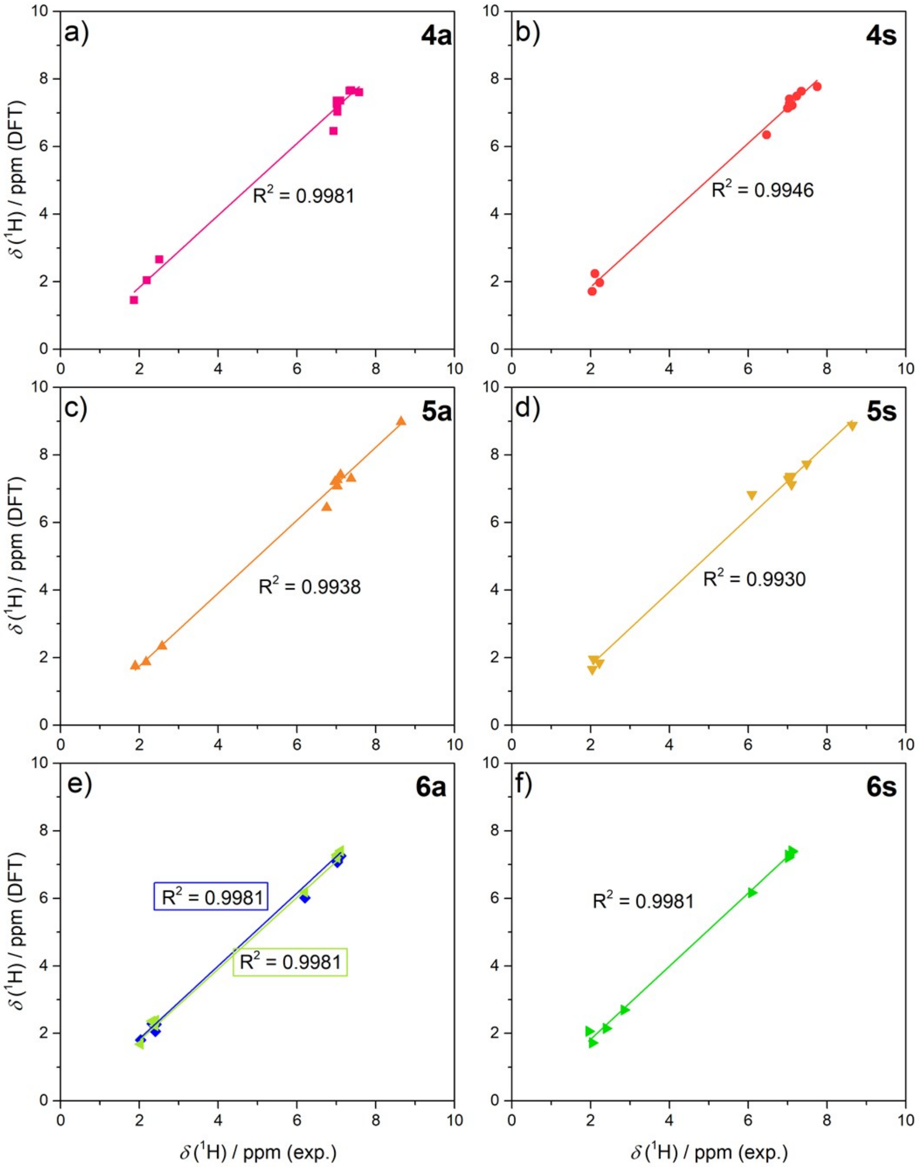


**Figure S3.** The correlation of the experimental and the B3LYP/6-311G* DFT predicted $^1$H chemical shifts of a) **4a**, b) **4s**, c) **5a**, d) **5s**, e) **6a**, f) **6s**. DFT predicted shifts for magnetically equivalent $CH_3$, Ph and pyridyl protons were averaged, and the data are compared for the asymmetric part of the dimer (corresponding to the mononuclear $Ni^{II}$ complex). e) DFT calculations were performed for two geometries of **6a**. Blue diamonds correspond to the **6a** optimized geometry based on SC-XRD structure; green triangles correspond to the **6a** optimized geometry with terminal phenyls rotation akin to the **4a** SC-XRD structure. The correlation coefficient $R^2$ from the linear regression analysis is displayed in each panel.

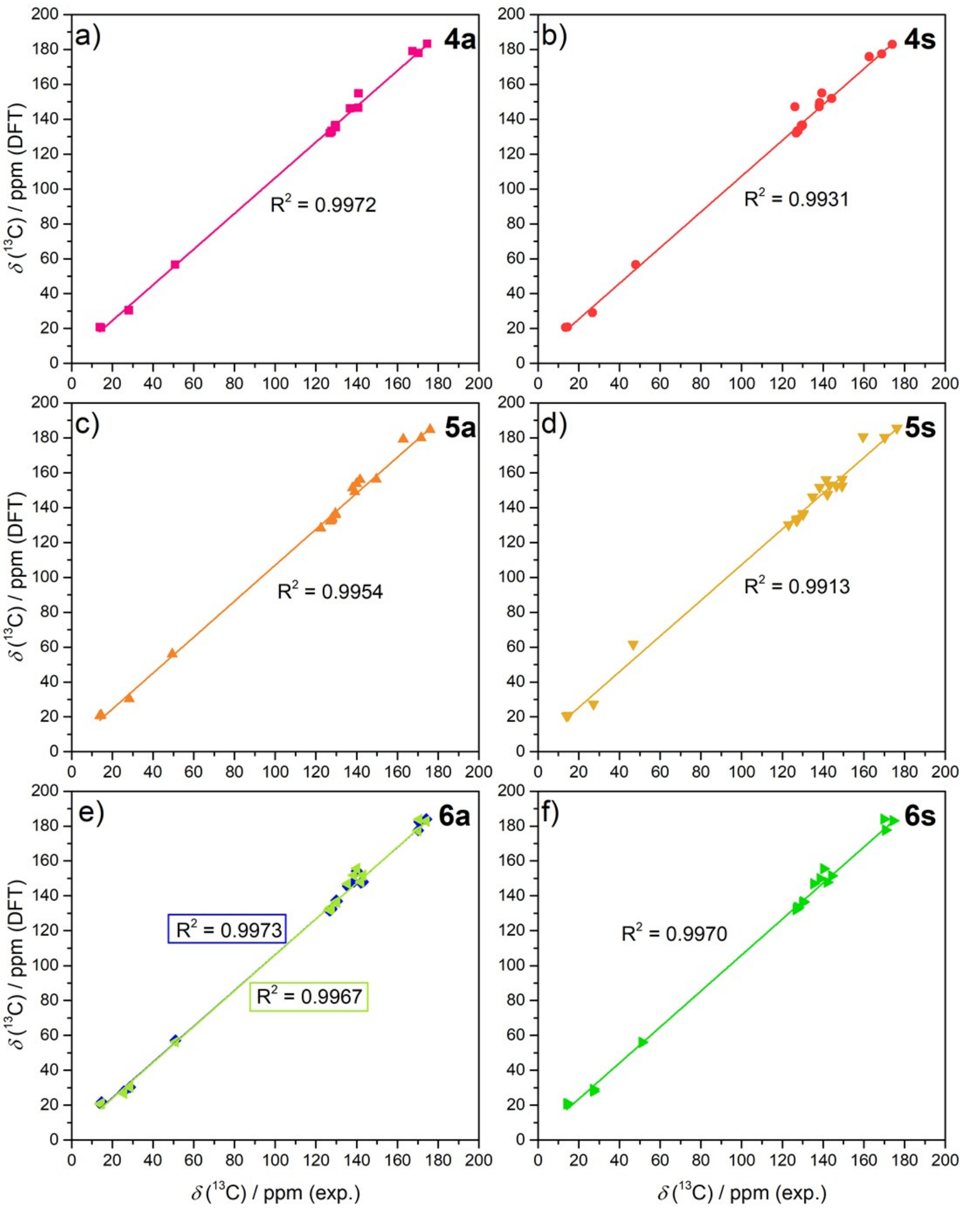


**Figure S4.** The correlation of the experimental and the B3LYP/6-311G* DFT predicted $^{13}C$ chemical shifts of a) **4a**, b) **4s**, c) **5a**, d) **5s**, e) **6a**, f) **6s**. DFT predicted shifts for magnetically equivalent $CH_3$ and Ph protons were averaged, and the data are compared for the asymmetric part of the dimer (corresponding to the mononuclear $Ni^{II}$ complex). e) DFT calculations were performed for two geometries of **6a**. Blue diamonds correspond to the **6a** optimized geometry based on SC-XRD structure; green triangles correspond to the **6a** optimized geometry with terminal phenyls rotation akin to the **4a** SC-XRD structure. The correlation coefficient $R^2$ from the linear regression analysis is displayed in each panel.

**Table S3.** The assignment of the $^1$H NMR chemical shifts, comparison of the experimental and the B3LYP/6-311G* DFT predicted NMR shifts.

| | **4a** | | **4s** | | **5a** | | **5s** | | **6a** | | | **6s** | |
|---|---|---|---|---|---|---|---|---|---|---|---|---|---|
| | $\delta$ ($^1$H)/ ppm | | | | | | | | | | | | |
| [a] | exp. | DFT | exp. | DFT | exp. | DFT | exp. | DFT | exp. | DFT[b] | DFT'[c] | exp. | DFT |
| H8 | 6.93 | 6.46 | 6.47 | 6.35 | 6.75 | 6.28 | 6.09 | 6.65 | 6.2 | 6.01 | 6.21 | 6.08 | 6.10 |
| H16 | 1.87 | 1.45 | 2.04 | 1.71 | 1.89 | 1.71 | 2.04 | 1.62 | 2.03 | 1.80 | 1.64 | 2.04 | 1.68 |
| H17 | 2.19 | 2.03 | 2.23 | 1.97 | 2.17 | 1.84 | 2.22 | 1.74 | 2.4 | 2.06 | 2.14 | 2.39 | 2.04 |
| H19 | 7.03 | 7.03 | 7.00 | 7.13 | 7.02 | 7.03 | 7.1 | 7.09 | 7.02 | 7.19 | 7.12 | 7.02 | 7.13 |
| H20 | 7.01 | 7.36 | 7.05 | 7.41 | 7.10 | 7.26 | 7.06 | 7.22 | 7.11 | 7.25 | 7.24 | 7.11 | 7.24 |
| H21 | 7.10 | 7.36 | 7.12 | 7.22 | 7.10 | 7.25 | 7.09 | 7.22 | 7.11 | 7.25 | 7.24 | 7.11 | 7.23 |
| H22 | 7.01 | 7.36 | 7.05 | 7.41 | 7.10 | 7.26 | 7.06 | 7.22 | 7.11 | 7.25 | 7.24 | 7.11 | 7.25 |
| H23 | 7.03 | 7.03 | 7.00 | 7.13 | 7.02 | 7.03 | 7.1 | 7.09 | 7.02 | 7.19 | 7.12 | 7.02 | 7.24 |
| H24 | 2.51 | 2.66 | 2.11 | 2.24 | 2.57 | 2.34 | 2.07 | 1.85 | 2.32 | 2.29 | 2.30 | 1.95 | 2.05 |
| H25 | – | – | – | – | – | – | – | – | 2.42 | 2.26 | 2.37 | 2.85 | 2.64 |
| H26 | 7.58 | 7.60 | 7.75 | 7.78 | 7.37 | 7.20 | 7.48 | 8.12 | – | – | – | – | – |
| H27 | 7.34 | 7.65 | 7.35 | 7.64 | 8.64 | 8.92 | 8.64 | 8.87 | – | – | – | – | – |
| H28 | 7.38 | 7.67 | 7.23 | 7.50 | – | – | – | – | – | – | – | – | – |
| H29 | 7.34 | 7.65 | 7.35 | 7.64 | 8.64 | 8.92 | 8.64 | 8.87 | – | – | – | – | – |
| H30 | 7.58 | 7.60 | 7.75 | 7.78 | 7.37 | 7.20 | 7.48 | 8.12 | – | – | – | – | – |
| H32 | 7.03 | 7.25 | 7.05 | 7.19 | 6.96 | 7.14 | 7.02 | 7.25 | 7.02 | 7.07 | 7.18 | 7.02 | 7.17 |
| H33 | 7.01 | 7.28 | 7.06 | 7.29 | 7.02 | 7.11 | 7.05 | 7.19 | 7.02 | 7.10 | 7.13 | 7.02 | 7.11 |
| H34 | 7.01 | 7.26 | 7.05 | 7.28 | 7.02 | 7.11 | 7.05 | 7.19 | 7.02 | 7.13 | 7.12 | 7.02 | 7.11 |
| H35 | 7.01 | 7.28 | 7.06 | 7.29 | 7.02 | 7.11 | 7.05 | 7.19 | 7.02 | 7.10 | 7.13 | 7.02 | 7.11 |
| H36 | 7.03 | 7.25 | 7.05 | 7.19 | 6.96 | 7.14 | 7.02 | 7.25 | 7.02 | 7.07 | 7.18 | 7.02 | 7.17 |

[a] Atom numbering according to the SC-XRD structures in Figs. 1-4, S1 and Table S1. DFT predicted shifts for magnetically equivalent $CH_3$, Ph and pyridyl protons were averaged. [b] Chemical shifts for the DFT-optimized geometry based on the **6a** SC-XRD structure. [c] Chemical shifts for the DFT-optimized geometry of **6a** with terminal phenyls rotation akin to the **4a** SC-XRD structure.

**Table S4.** The assignment of the $^{13}C$ NMR chemical shifts, comparison of the experimental and the B3LYP/6-311G* DFT predicted NMR shifts.

| | **4a** | | **4s** | | **5a** | | **5s** | | **6a** | | | **6s** | |
|---|---|---|---|---|---|---|---|---|---|---|---|---|---|
| | δ ($^{13}C$)/ ppm | | | | | | | | | | | | |
| [a] | exp.[b] | DFT | exp.[b] | DFT | exp. | DFT | exp. | DFT | exp. | DFT[c] | DFT'[d] | exp. | DFT |
| C1 | 140.93 | 154.81 | 139.38 | 154.98 | 141.68 | 155.09 | 141.54 | 154.84 | 140.06 | 154.24 | 154.64 | 140.38 | 154.29 |
| C4 | 170.37 | 177.94 | 168.90 | 177.38 | 171.66 | 180.40 | 170.23 | 181.42 | 170.18 | 177.59 | 178.55 | 170.73 | 179.55 |
| C7 | 140.82 | 146.59 | 144.25 | 151.94 | 139.00 | 145.26 | 143.21 | 149.16 | 143.14 | 148.05 | 147.85 | 144.16 | 147.24 |
| C8 | 50.88 | 56.65 | 48.07 | 56.56 | 49.44 | 56.84 | 46.74 | 58.83 | 50.98 | 57.12 | 56.87 | 51.03 | 55.60 |
| C9 | 167.47 | 178.99 | 162.73 | 175.83 | 162.85 | 177.64 | 159.60 | 179.84 | 170.65 | 182.41 | 182.00 | 169.87 | 181.84 |
| C12 | 174.67 | 183.34 | 174.00 | 182.92 | 176.07 | 185.38 | 176.24 | 185.56 | 174.22 | 184.08 | 183.74 | 174.47 | 183.94 |
| C15 | 138.56 | 150.60 | 138.37 | 149.45 | 138.01 | 150.64 | 138.27 | 151.14 | 138.61 | 148.05 | 147.85 | 138.59 | 149.53 |
| C16 | 14.57 | 20.49 | 14.54 | 20.82 | 14.47 | 21.34 | 14.79 | 21.17 | 14.73 | 21.75 | 21.20 | 14.79 | 21.22 |
| C17 | 13.92 | 20.73 | 13.57 | 20.59 | 13.82 | 20.85 | 13.98 | 20.72 | 14.09 | 20.83 | 20.90 | 14.12 | 20.89 |
| C18 | * | 146.68 | * | 147.02 | * | 145.57 | 134.89 | 145.88 | 135.57 | 145.54 | 146.55 | 135.48 | 146.58 |
| C19 | 129.60 | 136.62 | 129.55 | 136.41 | 129.62 | 137.40 | 130.11 | 137.18 | 129.98 | 137.44 | 137.20 | 130.33 | 137.25 |
| C20 | 127.60 | 133.07 | 127.30 | 133.17 | 127.46 | 132.32 | 127.01 | 132.26 | 127.31 | 131.90 | 132.22 | 127.06 | 132.19 |
| C21 | 127.78 | 133.39 | 127.45 | 133.27 | 127.97 | 133.06 | 127.76 | 132.94 | 127.68 | 132.51 | 132.61 | 127.70 | 132.59 |
| C22 | 127.60 | 133.07 | 127.30 | 133.17 | 127.46 | 132.32 | 127.01 | 132.26 | 127.31 | 131.90 | 132.22 | 127.06 | 132.19 |
| C23 | 129.60 | 136.62 | 129.55 | 136.41 | 129.62 | 137.40 | 130.11 | 137.18 | 129.98 | 137.44 | 137.20 | 130.33 | 137.25 |
| C24 | 28.11 | 30.46 | 26.79 | 29.04 | 28.33 | 30.17 | 27.20 | 27.52 | 28.69 | 30.32 | 30.31 | 27.18 | 29.29 |
| C25 | 136.74 | 146.17 | 138.16 | 147.02 | 139.96 | 152.97 | 146.56 | 151.46 | 25.73 | 27.91 | 27.26 | 27.29 | 27.97 |
| C26 | 129.86 | 135.33 | 129.45 | 136.42 | 122.37 | 128.08 | 123.06 | 129.86 | – | – | – | – | – |
| C27 | 127.71 | 133.06 | 127.70 | 133.24 | 149.61 | 155.66 | 149.28 | 155.80 | – | – | – | – | – |
| C28 | 129.75 | 135.46 | 129.90 | 136.57 | – | – | – | – | – | – | – | – | – |
| C29 | 127.71 | 133.06 | 127.70 | 133.24 | 149.61 | 155.66 | 149.28 | 155.80 | – | – | – | – | – |
| C30 | 129.86 | 135.33 | 129.45 | 136.42 | 122.37 | 128.08 | 123.06 | 129.86 | – | – | – | – | – |
| C31 | * | 147.75 | 138.20 | 147.89 | * | 146.81 | 142.10 | 146.90 | 142.17 | 147.55 | 147.33 | 142.05 | 147.29 |
| C32 | 129.30 | 135.92 | 129.30 | 136.00 | 129.62 | 135.85 | 130.11 | 135.96 | 130.34 | 136.97 | 136.15 | 129.96 | 136.07 |
| C33 | 127.60 | 132.79 | 127.30 | 132.75 | 127.46 | 132.07 | 127.37 | 132.31 | 127.05 | 131.70 | 132.02 | 127.32 | 132.02 |
| C34 | 126.90 | 132.02 | 126.90 | 131.98 | 126.97 | 131.47 | 126.97 | 131.85 | 126.69 | 131.48 | 131.24 | 126.73 | 131.31 |
| C35 | 127.60 | 132.79 | 127.30 | 132.75 | 127.46 | 132.07 | 127.37 | 132.31 | 127.05 | 131.70 | 132.02 | 127.32 | 132.02 |
| C36 | 129.30 | 135.92 | 129.30 | 136.00 | 129.62 | 135.85 | 130.11 | 135.96 | 130.34 | 136.97 | 136.15 | 129.96 | 136.07 |

[a] Atom numbering according to the SC-XRD structures in Figs. 1-4, S1 and Table S1. DFT predicted shifts for magnetically equivalent $CH_3$, Ph and pyridyl protons were averaged. [b] $^{13}C$ experimental chemical shifts are obtained from the correlation peaks in the 2D $^{1}H$–$^{13}C$ HSQC, HMBC spectra. [c] Shifts for the DFT-optimized geometry based on the **6a** SC-XRD structure. [d] Shifts for the DFT-optimized geometry of **6a** with terminal phenyls rotation akin to the **4a** SC-XRD structure. * labels atoms where the experimental assignments are missing due to the limited signal-to-noise ratio in the NMR spectra.

# Kinetics of isomerization (epimerization)

The kinetics of the **5a→5s** and **6s→6a** epimerization reaction was followed by $^1$H NMR in $CD_3Cl$. The molar ratio at the given reaction time point was evaluated via integration of the signals of (at least) five resonances for each isomer. The experimental kinetic traces were analyzed using two kinetic models (Figure S5):

1. First-order kinetics was assumed, and the experimental data were fitted with integrated rate law equation (example for the **5a→5s** reaction)

$$x(\mathbf{5a}) = e^{-kt}$$

where *x* is the molar ratio of the decaying isomer, *t is* the reaction time, and *k is* the first order rate constant.

2. A model for reaction approaching equilibrium (e.g. $\mathbf{5a} \underset{k_\mathrm{b}}{\overset{k_\mathrm{f}}{\rightleftharpoons}} \mathbf{5s}$) was employed by fitting the data with integrated rate law of the form

$$x(\mathbf{5a}) = x_0(\mathbf{5a}) - \frac{k_\mathrm{f} x_0(\mathbf{5a}) - k_\mathrm{b}\left(1 - x_0(\mathbf{5a})\right)}{k_\mathrm{f} + k_\mathrm{b}}\left(1 - e^{-(k_\mathrm{f}+k_\mathrm{b})t}\right), \quad K = \frac{k_\mathrm{f}}{k_\mathrm{b}}$$

where *x* is the molar ratio of the decaying isomer, the $x_0$ molar ratio in the initial solution, *t is* the reaction time and $k_\mathrm{f}$ *is* the first-order rate constant for the forward reaction, $k_\mathrm{b}$ the first order rate constant for the backward reaction, and *K* is the equilibrium constant. According to the recommendation in A. Genaev, et al., *Org. Biomol. Chem.* 2019, 17, 3781-3789, $k_\mathrm{f}$ and *K* were optimized as independent parameters in the fit.

The rate constants and the *K* values obtained are summarized in Table S5.

Note that, since we haven't followed the **5** epimerization up to the point where equilibrium could have been reached, the estimated *K*(s) might be inaccurate. Nevertheless, the relatively invariable value of $K \approx 9$ estimated by the equilibrium model fit, at all investigated temperatures, is consistent with the **5s**/**5a** molar ratio of 92/8 found in an aged reaction mixture sample.

The activation parameters of epimerization **5** were extracted from the temperature dependence of *k* and $k_\mathrm{f}$ using Eyring,

$$\ln\left(\frac{k}{T}\right) = -\frac{\Delta H^{\ddagger}}{RT} + \frac{\Delta S^{\ddagger}}{R} + \ln\left(\frac{k_\mathrm{B}}{h}\right)$$

and Arrhenius equations

$$\ln(k) = Ae^{-\frac{E_\mathrm{a}}{RT}}$$

where, $T$ stands for the temperature, $R$, $k_B$, $h$ are the Gas, Boltzmann and Planck constants, respectively, and the $\Delta H^{\ddagger}$ and $\Delta S^{\ddagger}$ are the activation enthalpy and entropy, $E_a$ is the activation energy and $A$ the frequency factor. The activation parameters are displayed in the insets of Figure S5a,c.

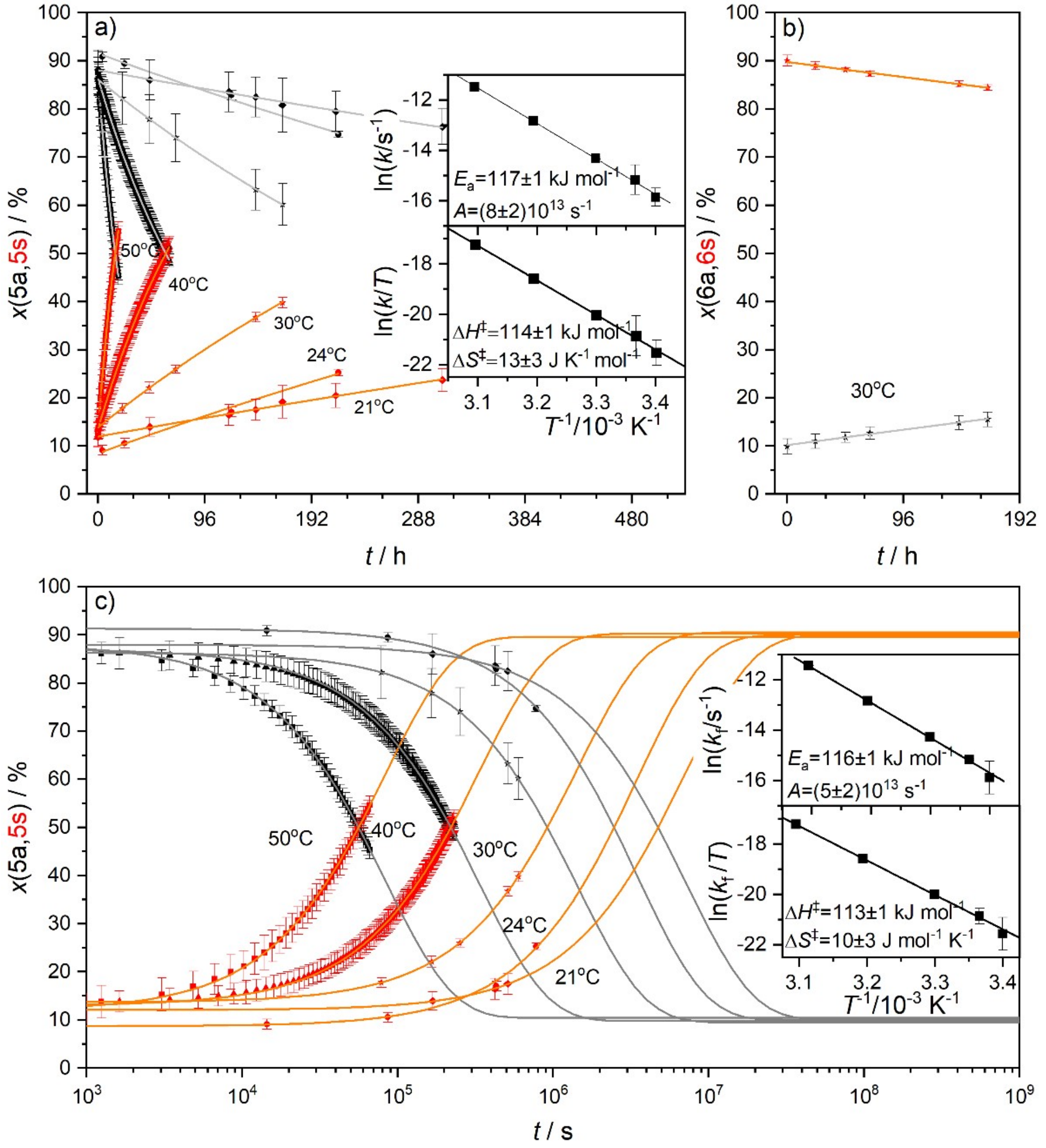


**Figure S5.** a) The kinetics of the **5a** to **5s** epimerization in $CDCl_3$ followed by $^1H$ NMR in the 21-50°C range. The circles correspond to the **5a** (black) and **5s** (red) molar ratio at a given reaction timepoint. Error bars reflect the deviation within two independent runs, and involve the integration errors of (at least) five resonances in the $^1H$ spectrum used for quantification of isomer concentration. Gray and orange lines represent the fits of the first order kinetic equation. Insets shows the analysis of rate constant temperature dependence using Arrhenius and Eyring equations, and the activation parameters. b) The kinetics of the **6s** to **6a** isomerization at 30°C. The rate of the conversion was found to be roughly five times slower than the isomerization of **5** (see Table S5). c) Evaluation of **5a** to **5s** epimerization kinetics with the model of

a reaction approaching equilibrium involving forward and backward reactions. Inset: Arrhenius and Eyring analysis of the temperature dependence of the forward reaction rate constant with corresponding activation parameters.

**Table S5**. The first-order rate constants of isomerization (epimerization) of **5a→5s** and **6s→6a** in $CD_3Cl$ and the parameters the $\mathbf{5a} \underset{k_b}{\overset{k_f}{\rightleftharpoons}} \mathbf{5s}$ kinetic model

| | **5a→5s** | **6s→6a** | $\mathbf{5a} \underset{k_b}{\overset{k_f}{\rightleftharpoons}} \mathbf{5s}$ | |
|---|---|---|---|---|
| $T$ /K | $k$ / $10^{-7}s^{-1}$ | | $k_f$ / $10^{-7}s^{-1}$ | $K$ |
| 294 | 1.30 ± 0.03 | – | 1.3 ± 0.8 | 9 ± 300 |
| 297 | 2.6 ± 0.1 | – | 2.58 ± 0.08 | 9 ± 24 |
| 303 | 6.03 ± 0.02 | 1.04 ± 0.04 | 6.30 ± 0.08 | 9.5 ± 3.1 |
| 313 | 26.7 ± 0.1 | – | 26.80 ± 0.08 | 9.2 ± 0.5 |
| 323 | 104 ± 1 | – | 107.5 ± 0.9 | 8.6 ± 1.2 |

## Electrochemistry and spectroelectrochemistry

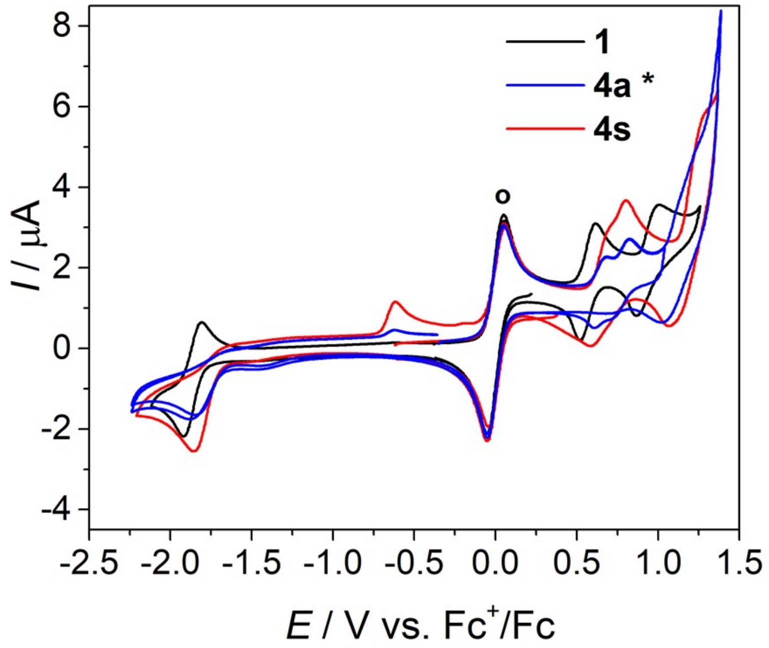


**Figure S6.** Cyclic voltammograms of 0.5 mM **1** (black trace), **4s** (red trace) in the presence of 0.5 mM **ferrocene** (o, Fc) (two scans, scan rate 200 mV $s^{-1}$) in $CH_2Cl_2$ /$n$$Bu_4NPF_6$. CV of complex **4a,** with limited solubility in $CH_2Cl_2$ at saturated concentration about 0.2 mM (*), in the presence of 0.5 mM Fc, is shown as the blue trace. Note that due to the smaller size of the Fc molecule its diffusion coefficient is higher than for the mono or dinuclear Ni(II) macrocyclic complexes and results in larger peak currents at identical molar concentration.

**Table S6**. Electrochemical data for oxidation of selected mononuclear (**1**) and dinuclear (**4a**, **4s**) Ni(II) complexes derived from the square-wave voltammetry in $CH_2Cl_2$/$n$-$Bu_4NPF_6$ solutions using Pt working electrode. Anodic peak potentials ($E_{pa}$) for the first (ox1), second (ox2), third (ox3) and fourth (ox4) electron transfer respectively, are in volts vs $Fc^+$/Fc.

| complex | $E_{pa}^{ox1}$ | | $E_{pa}^{ox2}$ | |
|---|---|---|---|---|
| **1** | 0.56 | | 0.93 | |
| | $E_{pa}^{ox1}$ | $E_{pa}^{ox2}$ | $E_{pa}^{ox3}$ | $E_{pa}^{ox4}$ |
| **4a** | 0.63 | 0.76 | 1.17 | 1.25 |
| **4s** | 0.60 | 0.69 | 1.17 | 1.17 |

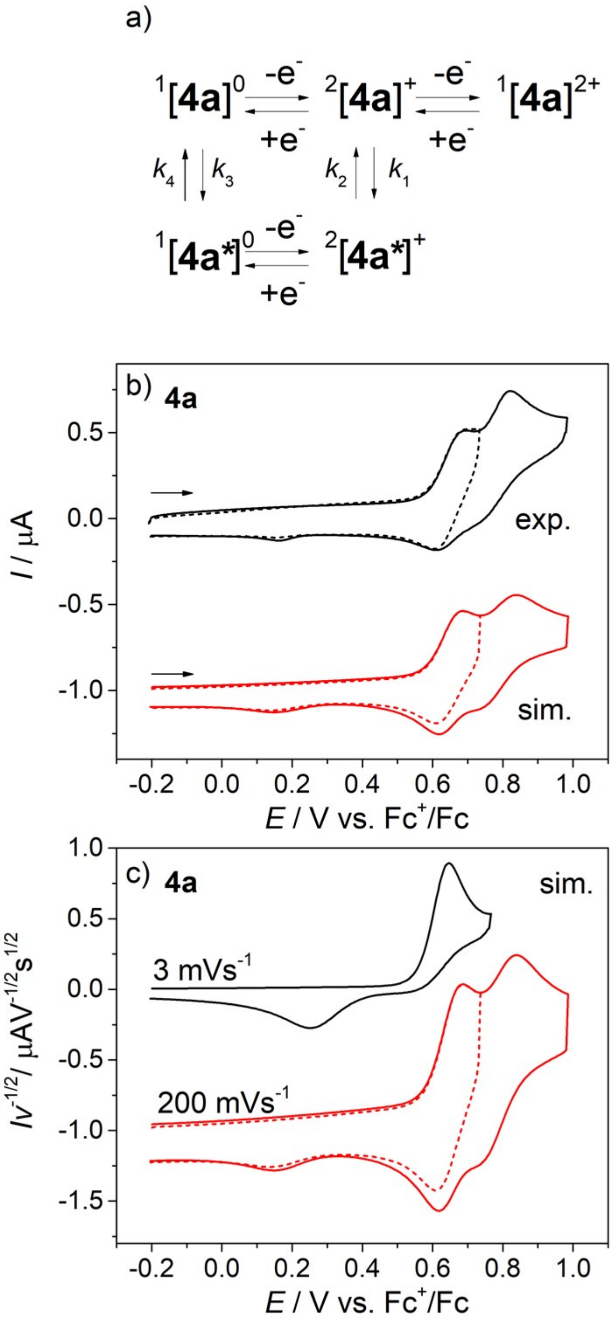


**Figure S7.** a) The mechanism proposed for the electro-chemical transformations of **4a** during the removal of the first two electrons; b) experimental CV (black) and digital simulations of CV (red - DigiElch Professional software from Gamry Instruments (USA), version Digielch8, was used for simulations) of **4a** at 200 mVs$^{-1}$ according to the mechanism in a). Parameters: $E^{o}{}_{,ox1}$ = +0.65 V, $E^{o}{}_{,ox2}$ = +0.79 V for **4a** and $E$ $^{o}{}_{,ox1}$ = +0.27 V for **4a*** vs Fc$^+$/Fc, $k_s$ = 0.05 cm s$^{-1}$, $\alpha$ = 0.5, $D$ = 10$^{-5}$ cm$^2$ s$^{-1}$, $k_1$ = 0.6 s$^{-1}$, $k_2$ = 0.006 s$^{-1}$, $k_3$ = 1.6·10$^{-8}$ s$^{-1}$, $k_4$ = 6·10$^8$ s$^{-1}$. c) digital simulations at 3 mVs$^{-1}$ (black, for comparison with Fig. 10, inset) and 200 mVs$^{-1}$ (red)

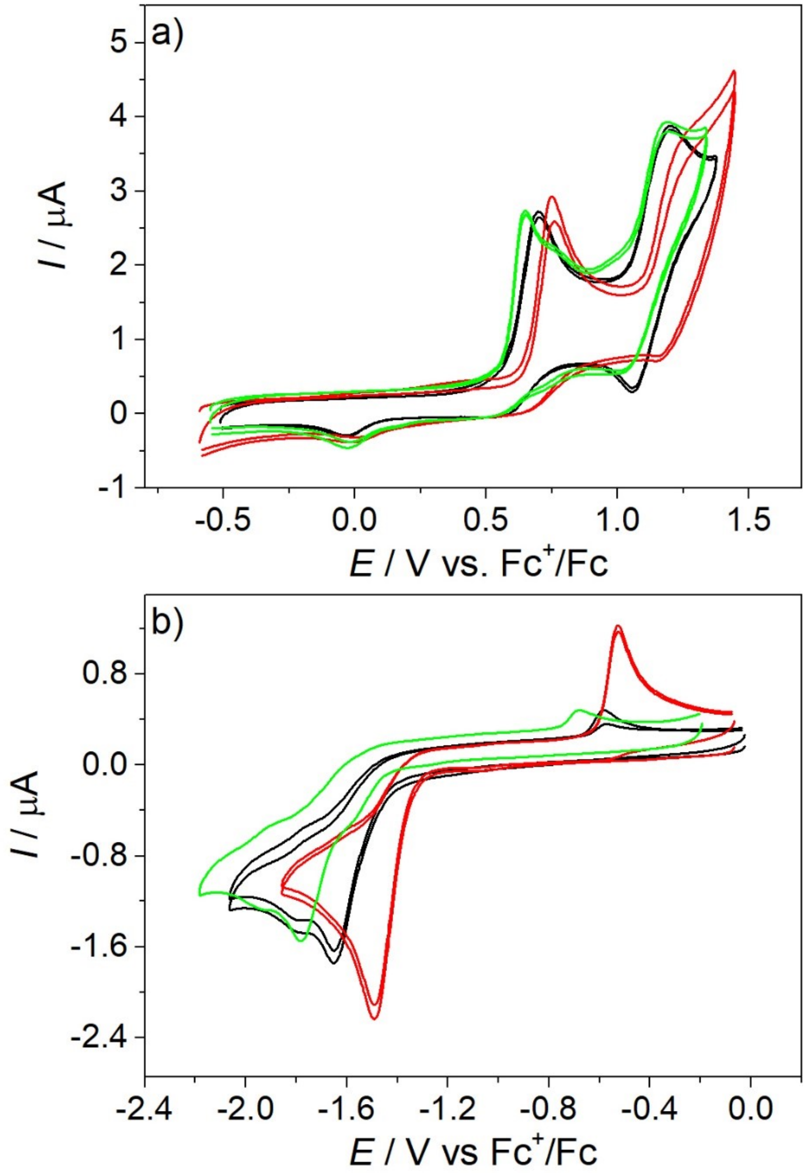


**Figure S8.** Cyclic voltammograms of **4s** (black traces), **5s** (red traces) and **6a** (green traces) in $CH_2Cl_2$-$CH_3CN$/$n$$Bu_4NPF_6$ (two scans, scan rate 100 mV $s^{-1}$).

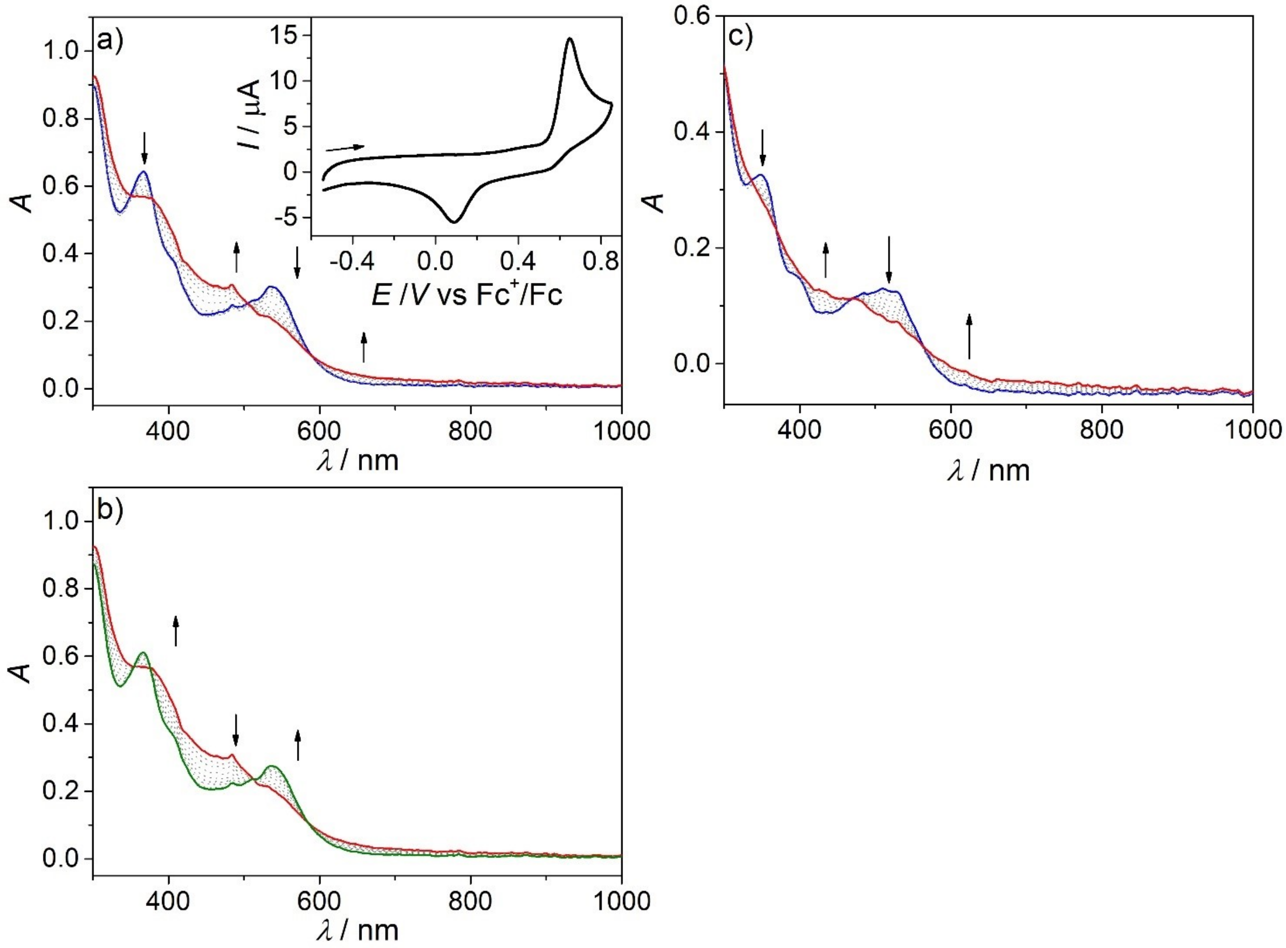


**Figure S9.** UV–vis–NIR spectra measured in the region of the first anodic 2-electron oxidation peak upon a) forward scan and b) during the backward scan of **4s** in $CH_2Cl_2/CH_3CN$ 1:1/*n*$Bu_4NPF_6$ under an argon atmosphere. Inset in a): in situ cyclic voltammogram (Pt-honeycomb working electrode, scan rate 10 mV $s^{-1}$). c) UV–vis–NIR spectra measured in the region of the first anodic double-peak upon forward scan of **6a** in $CH_2Cl_2/CH_3CN$ 1:1/*n*$Bu_4NPF_6$ under an argon atmosphere.

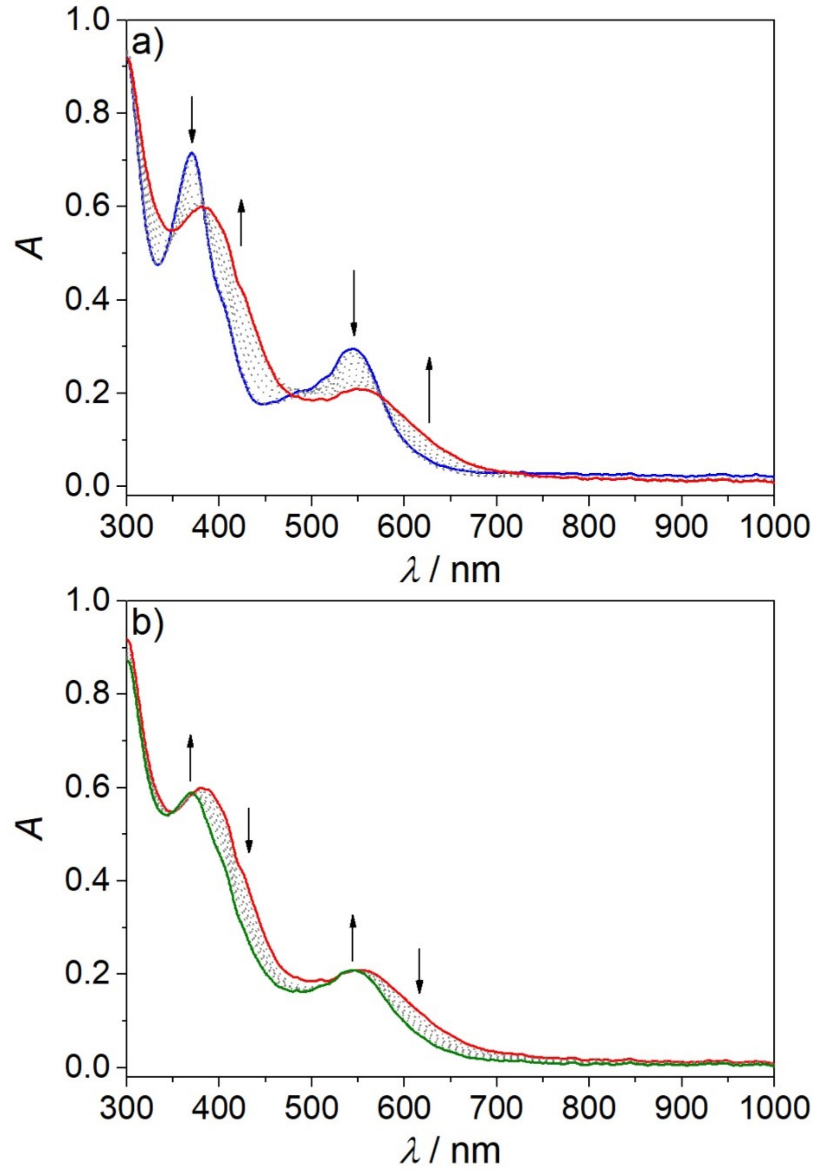


**Figure S10.** UV–vis–NIR spectra measured in the region of the first anodic peak upon (a) forward scan and (b) during the backward scan of **5s** in $CH_2Cl_2$/$CH_3CN$ 1:1/$n$$Bu_4NPF_6$ under an argon atmosphere (Pt-honeycomb working electrode, scan rate 10 mV $s^{-1}$).

## UV–vis–NIR spectroscopy

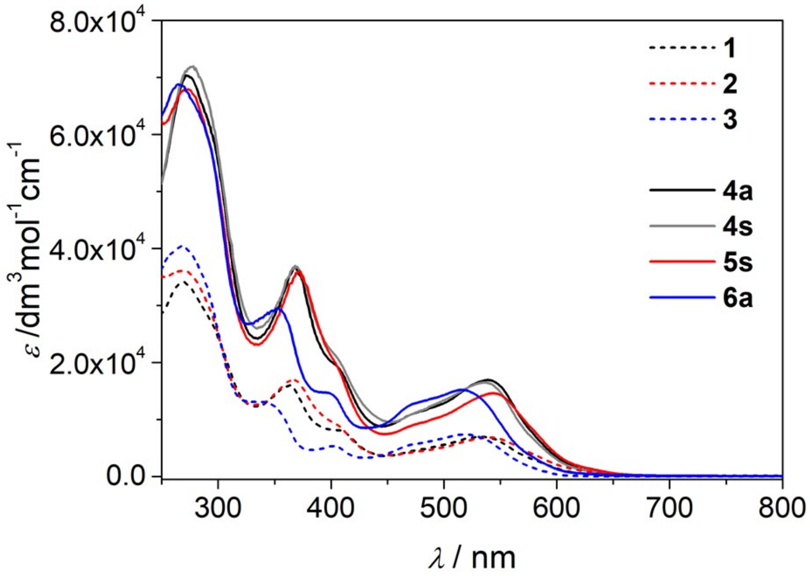


**Figure S11.** UV–vis spectra of (a) dimeric complexes **4a**, **4s**, **5s** and **6a** (solid lines) and (b) their corresponding monomeric precursors **1**, **2**, **3** in $CH_2Cl_2$.

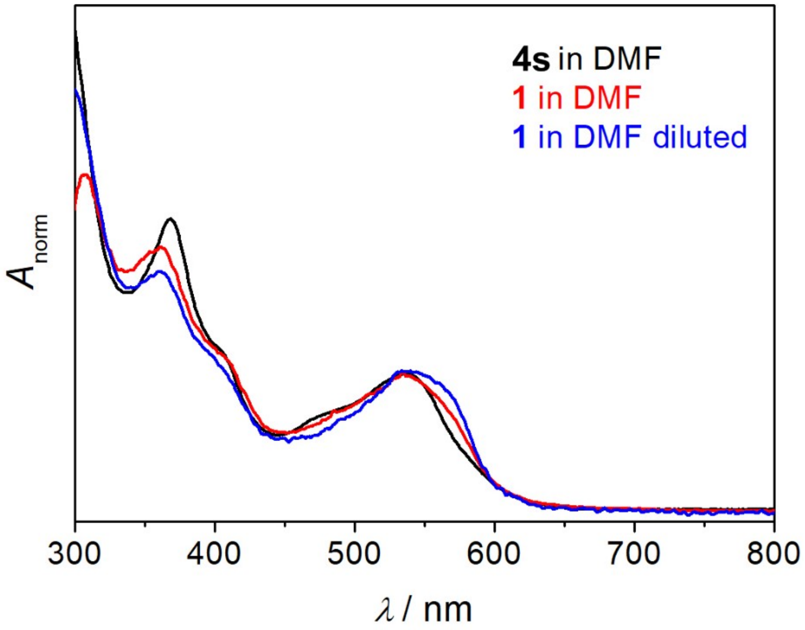


**Figure S12.** Normalized UV–vis spectra of dimeric complex **4s** (black trace) and of the corresponding monomer **1** (red trace) in DMF (blue trace – diluted monomer **1** in DMF). All the spectral traces are normalized to the absorption at 530 nm for comparison purposes.

# DFT calculations

Considering the CV experiment as well as the *anti*/*syn* isomerization, it is worthwhile to provide some detail about the tautomeric and other forms of **4a** showing rich conformational variability of the compounds studied (both the initial neutral ones, as well as the follow up oxidation products). First of all, it is worth noting that, we have found a geometry with the Ni-C8-C8$^{i}$-Ni$^{i}$ dihedral angle of 75.8° (denoted as $^{1}$[**4a$^{rot90}$**]$^{0}$ see Figure S14a) and this geometry is 38.9 kJ·mol$^{-1}$ above the experimental **4a** geometry with the 180° Ni-C8-C8$^{i}$-Ni$^{i}$ dihedral angle. In the case of $^{1}$[**4s$^{rot90}$**]$^{0}$, the difference to **4s** is 46.5 kJ·mol$^{-1}$ and the Ni-C8-C8$^{i}$-Ni$^{i}$ dihedral angle is 97.3°. A relaxed geometry with Ni-C8-C8$^{i}$-Ni$^{i}$ dihedral angle frozen to 0° (denoted as $^{1}$[**4a$^{rot180}$**]$^{0}$, see Figure S14b) is by 96.6 kJ·mol$^{-1}$ above **4a** (this geometry contains one imaginary frequency). Another option for a structural change of **4** is a proton transfer from the bridge carbon atoms to the nitrogen atoms of the macrocycle resulting in the species denoted $^{1}$[**4a**$_{H4}$]$^{0}$ (with the lowest energy structure being 123.6 kJ·mol$^{-1}$ above the reference **4a** one, although there are 8 such isomers for both C-H groups). In the case of the oxidized form, the energy difference between $^{2}$[**4a**$_{H4}$]$^{+}$ and $^{2}$[**4a**]$^{+}$ is of 73.5 kJ·mol$^{-1}$. Interestingly, the structure with twofold intramolecular proton transfer $^{2}$[**4a**$_{2xH4}$]$^{+}$ is shifted by 61.2 kJ·mol$^{-1}$ from $^{2}$[**4a**]$^{+}$. Furthermore, we could also find the $^{2}$[**4a**]$^{+}$ structure with the Ni-C8-C8$^{i}$-Ni$^{i}$ dihedral angle rotated to 81.5° and this geometry is by means of 33.4 kJ·mol$^{-1}$ above $^{2}$[**4a**]$^{+}$. In addition, several other structures have been found with respect to the structure variability and/or follow up products upon oxidation, one is related to bridging the two monomeric units via the C–N bond [**4a**$_{CN}$], a change of the orientation for the bridging C–C bond (*i.e.*, a change of orientation of the macrocycle and hydrogen on the particular sp$^{3}$ bridging carbon atom, a change similar to chair-boat conformations in cyclohexane) [**4a**$_{CH\text{-}CH}$] or a change of the macrocycle shape with respect to the nitrogen atom which is bonded to the central atom [**4a**$_{1\text{-}1'}$], see Figure S14 and Table S8. The [**4a**$_{1\text{-}1'}$] and [**4a**$_{CH\text{-}CH}$] structures are the closest in energy to the original [**4a**] structure when considering the neutral species (49.3 and 12.5 kJ·mol$^{-1}$, respectively) and oxidation (21.5 and 10.7 kJ·mol$^{-1}$, respectively). In the structures of $^{1}$[**4a**$_{CN}$]$^{0}$ and $^{2}$[**4a**$_{CN}$]$^{+}$, the energy difference to the **4a** references are 63.3 and 53.3 kJ·mol$^{-1}$, respectively. For completeness, neutral ($^{1}$[**4a**$_{h}$]$^{-}$ and $^{1}$[**4s**]$^{-}$) and oxidized ($^{2}$[**4a**$_{h}$]$^{0}$, $^{2}$[**4a**$_{h'}$]$^{0}$ or two $^{2}$[**4a**$_{hh'}$]$^{-}$) structures with one (h and h') and both (hh') C–H protons removed from [**4a**] are also compiled in Table S8.

In an attempt to explain the *anti-syn* isomerization of **4a** to **4s**, the option of an intramolecular proton transfer $^{1}$[**4a$_{H4}$**]$^{0}$ (see Supporting Information section DFT calculations) has been considered. A graphical representation of the energetics for several geometries of $^{1}$[**4'$_{H4}$**]$^{0}$ is summarized in Figure S16. Figure S16 shows that after the $^{1}$[**4a$_{H4}$**]$^{0}$ initiation, which can be based on a $^{1}$[**4a$^{rot90}$**]$^{0}$ or $^{1}$[**4a$^{rot180}$**]$^{0}$ intermediate,

several species exist which have a considerably perturbed geometry and can be considered as $^1$[**4'$_{H4}$**]$^0$ *syn-anti* intermediates. Still, one can anticipate a 100 kJ·mol$^{-1}$ barrier, that has to be overcome within the isomerization scheme itself: $^1$[**4a$_{H4}$**]$^0$ → $^1$[**4'$_{H4}$**]$^0$ → $^1$[**4s$_{H4}$**]$^0$. However, the energy difference between $^1$[**4a**]$^0$ and $^1$[**4a$_{H4}$**]$^0$ structures (123.6 kJ·mol$^{-1}$) is already comparable to the experimentally estimated energy barrier (114±1 kJ·mol$^{-1}$). Hence, the $^1$[**4'$_{H4}$**]$^0$ structure is above an intermediate geometry for the $^1$[**4a$_{H4}$**]$^0$ to $^1$[**4a$_{H4}$**]$^0$ isomerization. In addition, the deprotonation of **4a** can be considered as an alternative,[64] assuming that the flexibility of the deprotonated carbon atoms is nearly the same as for $^1$[**4'$_{H4}$**]$^0$. Still, we do not target the way how the **4a** system deprotonates, except of assuming that the bridging sp$^3$ C8 carbons can be considered a weak acid. We also note that the addition of excess triethylamine, as an organic base, to the solution of **4a** in $CH_2Cl_2$ did not lead to the change of the UV–vis spectra of the complex. Further details of the isomerization mechanism are currently under investigation in our lab and will be reported in due course.

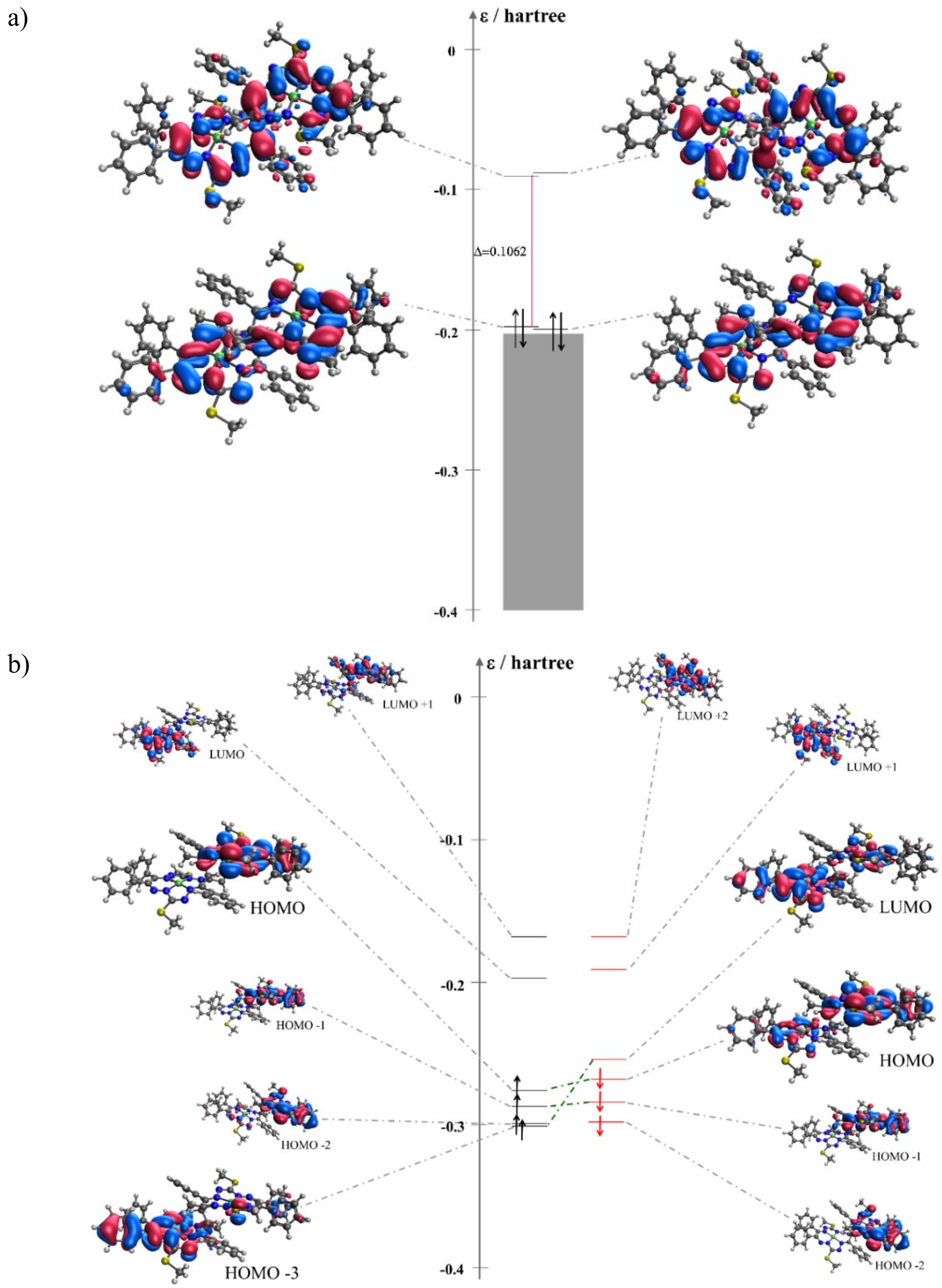


**Figure S13.** B3LYP/6-311G* frontier orbitals of a) $^1$[**4a**]$^0$ and b) $^2$[**4a**]$^+$ (isovalue 0.02 e.bohr$^{-3/2}$).

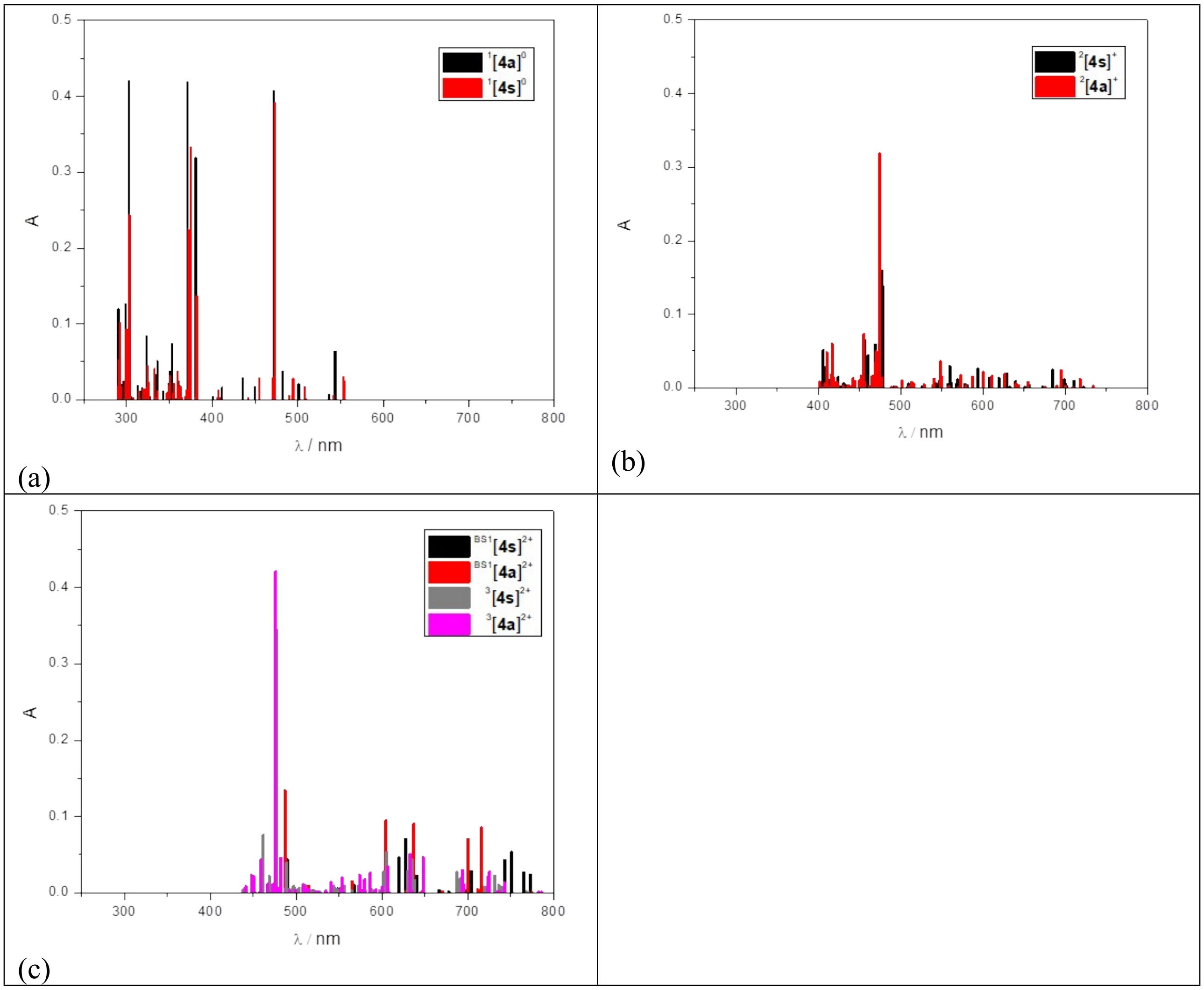


**Figure S14**. B3LYP/6-311G* TD transitions of (a) $^{1}$[**4s**]$^{0}$ and $^{1}$[**4a**]$^{0}$, (b) 1e-oxidized species of $^{2}$[**4s**]$^{+}$ and $^{2}$[**4a**]$^{+}$ and (c) 2e-oxidized species of [**4s**]$^{2+}$ and [**4a**]$^{2+}$.

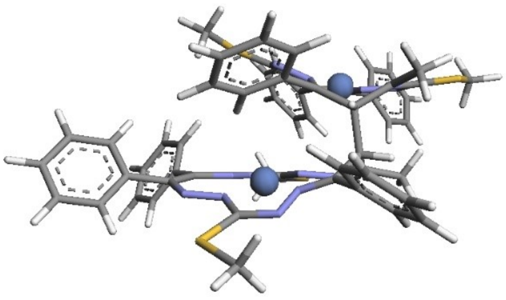

(a) ${}^{1}[\mathbf{4a}^{\mathrm{rot90}}]^{0}$

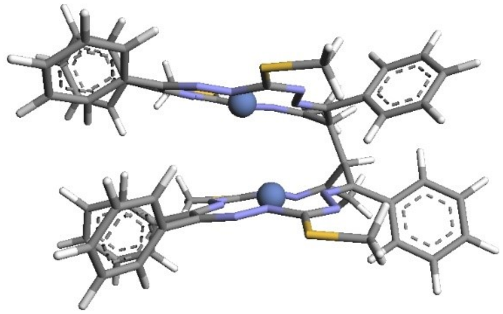

(b) ${}^{1}[\mathbf{4a}^{\mathrm{rot180}}]^{0}$

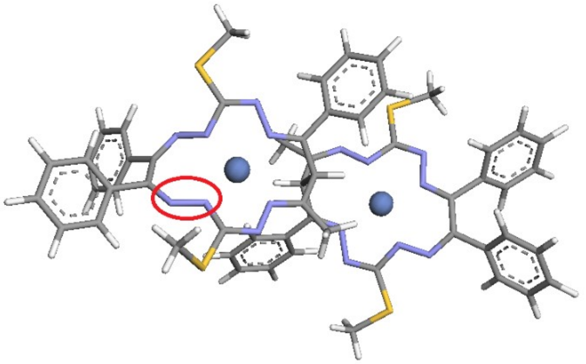

(c) ${}^{2}[\mathbf{4a}_{1\text{-}1'}]^{+}$

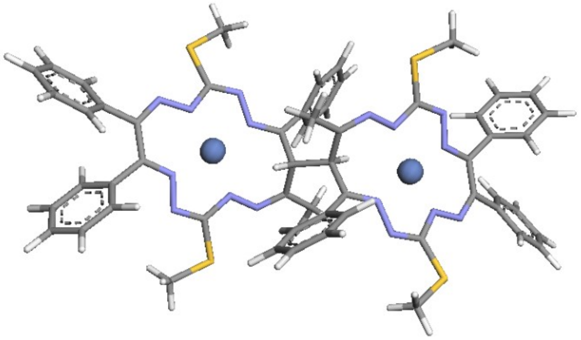

(d) ${}^{2}[\mathbf{4a}_{\mathrm{CH\text{-}CH}}]^{+}$

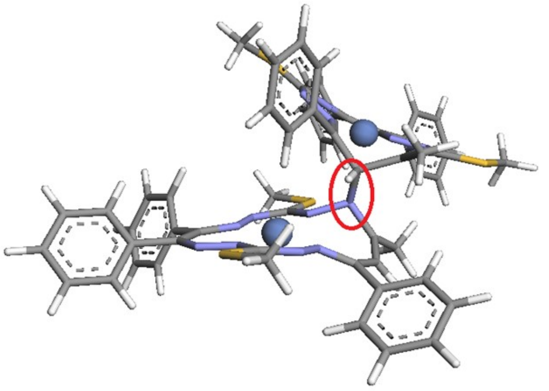

(e) ${}^{2}[\mathbf{4a}_{\mathrm{CN}}]^{+}$

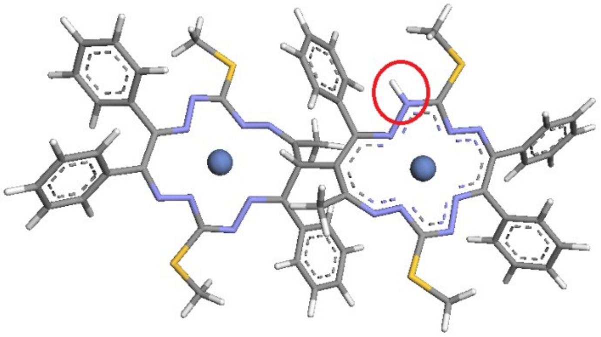

(f) ${}^{2}[\mathbf{4a}_{\mathrm{H4}}]^{+}$

**Figure S15.** Optimized structure of ${}^{1}[\mathbf{4a}^{\mathrm{rot90}}]^{0}$ with the 75.8° Ni-C8-C8$^{i}$-Ni$^{i}$ dihedral angle (a), the relaxed structure ${}^{1}[\mathbf{4a}^{\mathrm{rot180}}]^{0}$ with the 0° Ni-C8-C8$^{i}$-Ni$^{i}$ dihedral angle frozen and of optimized structures of oxidized species ${}^{2}[\mathbf{4a}_{1\text{-}1'}]^{+}$ (c), ${}^{2}[\mathbf{4a}_{\mathrm{CH\text{-}CH}}]^{+}$ (d), ${}^{2}[\mathbf{4a}_{\mathrm{CN}}]^{+}$ (e), ${}^{2}[\mathbf{4a}_{\mathrm{CH\text{-}C,H4}}]^{+}$ (f)

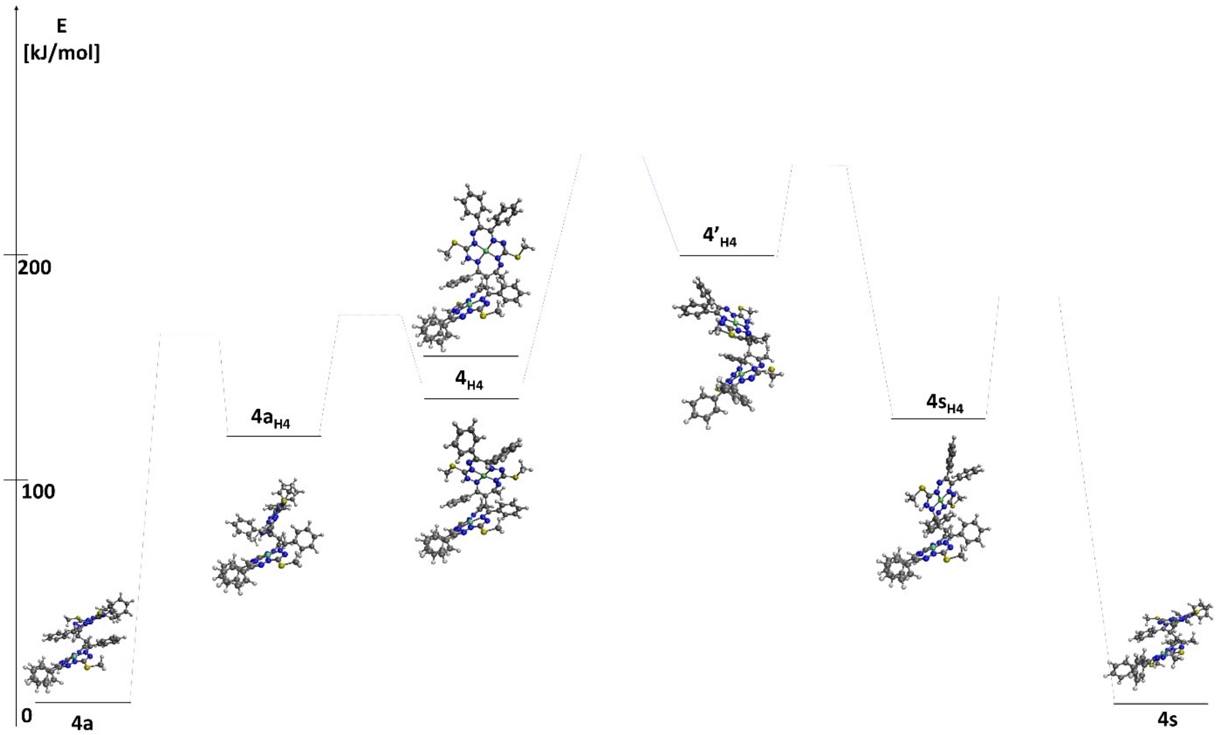


**Figure S16**. B3LYP/6-311G* energetics (free energies in kJ·mol$^{-1}$) of intermediate geometries of the isomerization for $^1$[$\mathbf{4_{H4}}$]$^0$ species (intramolecular proton transfer)

**Table S7**. B3LYP/6-311G* energies of different isomers of [**4**], [**5**] and [**6**] and different spin and charged species of [**4**].

| | $E_{opt}$ [hartree] | $S^2$ | q(Ni1) | q(Ni2) | spin(Ni1) | spin(Ni2) |
|---|---|---|---|---|---|---|
| $^{1}$[**4s**]$^{0}$ | -7650.163002 | 0 | 1.027 | 1.026 | 0 | 0 |
| $^{3}$[**4s**]$^{0}$ | -7650.129146 | 2.007 | 1.270 | 1.027 | 1.607 | 0 |
| $^{2}$[**4s**]$^{+}$ | -7649.966307 | 0.773 | 1.023 | 1.050 | 0 | 0.008 |
| $^{1}$[**4s**]$^{2+}$ | -7649.740916 | 0 | 1.069 | 1.068 | 0 | 0 |
| $^{3}$[**4s**]$^{2+}$ | -7649.760646 | 2.044 | 1.068 | 1.060 | 0.009 | 0.008 |
| $^{BS}$[**4s**]$^{2+}$ | -7649.760651 | 1.044 | 1.068 | 1.060 | 0.009 | -0.008 |
| $^{1}$[**4a**]$^{0}$ | -7650.161060 | 0 | 1.020 | 1.020 | 0 | 0 |
| $^{3}$[**4a**]$^{0}$ | -7650.085230 | 2.015 | 1.020 | 1.020 | 0.002 | 0.002 |
| $^{2}$[**4a**]$^{+}$ | -7649.964428 | 0.773 | 1.023 | 1.050 | 0.000 | 0.008 |
| $^{1}$[**4a**]$^{2+}$ | -7649.737013 | 0 | 1.055 | 1.057 | 0 | 0 |
| $^{3}$[**4a**]$^{2+}$ | -7649.758893 | 2.045 | 1.055 | 1.053 | 0.011 | 0.011 |
| $^{BS}$[**4a**]$^{2+}$ | -7649.758897 | 1.045 | 1.055 | 1.053 | 0.011 | -0.010 |
| $^{2}$[**4a**]$^{-}$ | -7650.266813 | 0.769 | 1.015 | 0.997 | 0.001 | -0.015 |
| $^{1}$[**4a**]$^{2-}$ | -7650.340318 | 0 | 0.991 | 0.992 | 0 | 0 |
| $^{3}$[**4a**]$^{2-}$ | -7650.354858 | 2.044 | 0.989 | 0.988 | -0.007 | -0.001 |
| $^{BS}$[**4a**]$^{2-}$ | -7650.354877 | 1.042 | 0.989 | 0.988 | -0.008 | -0.002 |
| $^{1}$[**5s**]$^{0}$ | -7682.247077 | 0 | 1.025 | 1.026 | 0 | 0 |
| $^{1}$[**5a**]$^{0}$ | -7682.245511 | 0 | 1.021 | 1.023 | 0 | 0 |
| $^{1}$[**6s**]$^{0}$ | -7266.627638 | 0 | 1.020 | 1.018 | 0 | 0 |
| $^{1}$[**6a**]$^{0}$ | -7266.628555 | 0 | 1.019 | 1.019 | 0 | 0 |

**Table S8**. B3LYP/6-311G* energies of chosen forms of [**4**] and [**4**]$^{+}$.

| | $E_{opt}$ [hartree] | S$^{2}$ | q(Ni1) | q(Ni2) | spin(Ni1) | spin(Ni2) |
|---|---|---|---|---|---|---|
| $^{1}[\mathbf{4s}^{\mathbf{rot90}}]^{0}$ | -7650.145281 | 0 | 1.026 | 1.026 | 0 | 0 |
| $^{1}[\mathbf{4a}^{\mathbf{rot90}}]^{0}$ | -7650.146255 | 0 | 1.032 | 1.015 | 0 | 0 |
| $^{1}[\mathbf{4a}^{\mathbf{rot180}}]^{0,a}$ | -7650.124257 | 0 | 1.006 | 1.010 | 0 | 0 |
| $^{1}[\mathbf{4a}_{\mathrm{CH\text{-}CH}}]^{0}$ | -7650.156308 | 0 | 1.015 | 1.015 | 0 | 0 |
| $^{1}[\mathbf{4a}_{1\text{-}1'}]^{0}$ | -7650.142264 | 0 | 1.022 | 1.004 | 0 | 0 |
| $^{1}[\mathbf{4a}_{\mathrm{CN}}]^{0}$ | -7650.136945 | 0 | 1.035 | 1.016 | 0 | 0 |
| $^{1}[\mathbf{4a}_{\mathrm{H4}}]^{0}$ | -7650.113973 | 0 | 1.030 | 1.026 | 0 | 0 |
| $^{2}[\mathbf{4a}^{\mathbf{rot90}}]^{+}$ | -7649.951714 | 0.772 | 1.068 | 1.020 | 0.012 | 0.000 |
| $^{2}[\mathbf{4a}^{\mathbf{rot180}}]^{+}$ | -7649.931949 | 0.765 | 1.021 | 1.020 | 0.011 | 0.010 |
| $^{2}[\mathbf{4a}_{\mathrm{CH\text{-}CH}}]^{+}$ | -7649.960343 | 0.774 | 1.018 | 1.050 | 0 | 0.004 |
| $^{2}[\mathbf{4a}_{1\text{-}1'}]^{+}$ | -7649.956253 | 0.770 | 1.024 | 1.058 | 0 | 0.070 |
| $^{2}[\mathbf{4a}_{\mathrm{CN}}]^{+}$ | -7649.944138 | 0.771 | 1.081 | 1.026 | 0.039 | 0.001 |
| $^{2}[\mathbf{4a}_{\mathrm{H4}}]^{+}$ | -7649.936417 | 0.777 | 1.052 | 1.037 | 0.022 | 0.012 |
| $^{2}[\mathbf{4a}_{\mathrm{2xH4}}]^{+}$ | -7649.941116 | 0.765 | 1.045 | 1.024 | 0.014 | 0.031 |
| $^{1}[\mathbf{4s}_{\mathrm{h}}]^{-}$ | -7649.649700 | 0 | 0.967 | 1.020 | 0 | 0 |
| $^{1}[\mathbf{4a}_{\mathrm{h}}]^{-}$ | -7649.649289 | 0 | 1.021 | 0.997 | 0 | 0 |
| $^{2}[\mathbf{4a}_{\mathrm{h}}]^{0}$ | -7649.505563 | 0.768 | 1.026 | 1.023 | 0.006 | 0.007 |
| $^{2}[\mathbf{4a}_{\mathrm{h'}}]^{0}$ | -7649.505577 | 0.770 | 1.024 | 1.026 | 0.007 | 0.006 |
| $^{2}[\mathbf{4a}_{\mathrm{hh'}}]^{-}$ | -7649.049240 | 0.760 | 0.996 | 0.995 | -0.004 | -0.002 |

[a] relaxed geometry for the frozen Ni-C8-C8$^{i}$-Ni$^{i}$ dihedral angle

**Table S9**. Crystal data and details of data collection for **4a**, **4s**, **5s** and **6a**.

| Compound | **4a** | **4s** | **5s** | **6a** |
|---|---|---|---|---|
| empirical formula | $C_{56}H_{50}N_{16}Ni_{26}S_4$ | $C_{58}H_{52}Ni_2Cl_6N_{16}S_4$ | $C_{54}H_{48}Ni_2N_{18}S_4$ | $C_{46}H_{646}Ni_2N_{16}S_4$ |
| fw | 1192.78 | 1431.51 | 1194.76 | 1068.65 |
| space group | $P2_1/n$ | $P\bar{1}$ | $I4_1/a$ | $P2_1/n$ |
| $a$ [Å] | 10.5994(11) | 11.30.32(11) | 34.235(5) | 10.6789(7) |
| $b$ [Å] | 14.0392(14) | 15.7119(8) | 34.235(5) | 18.7474(13) |
| $c$ [Å] | 21.2185(17) | 19.9582(12) | 20.889(4) | 11.7267(7) |
| $\alpha$ [°] | | 92.547(2) | | |
| $\beta$ [°] | 100.972(4) | 105.088(3) | | 91.7267(7) |
| $\gamma$ [°] | | 108.429(3) | | |
| $V$ [Å$^3$] | 3099.7(5) | 3110.2(4) | 24483(9) | 2346.6(3) |
| $Z$ | 2 | 2 | 16 | 2 |
| $\lambda$ [Å] | 0.71073 | 0.71073 | 0.71073 | 0.71073 |
| $\rho_{calcd}$ [g cm$^{-3}$] | 1.278 | 1.529 | 1.297 | 1.512 |
| crystal size [mm] | 0.04 × 0.03 × 0.02 | 0.08 × 0.04 × 0.03 | 0.05× 0.04 × 0.04 | 0.09 × 0.08 × 0.04 |
| $T$ [K] | 100(2) | 100(2) | 100(2) | 100(2) |
| $\mu$ [mm$^{-1}$] | 0.760 | 1.051 | 0.771 | 1.035 |
| $R_1$[a] | 0.0459 | 0.0426 | 0.0543 | 0.0766 |
| $wR_2$[b] | 0.1160 | 0.1057 | 0.1736 | 0.1803 |
| GOF[c] | 1.038 | 1.014 | 1.032 | 1.115 |

[a] $R_1 = \Sigma||F_o| - |F_c||/\Sigma|F_o|$. [b] $wR_2 = \{\Sigma[w(F_o^2 - F_c^2)^2]/\Sigma[w(F_o^2)^2]\}^{1/2}$. [c] $GOF = \{\Sigma[w(F_o^2 - F_c^2)^2]/(n - p)\}^{1/2}$, where $n$ is the number of reflections and $p$ is the total number of parameters refined.
